# Characterization of a Yellow Laccase from *Botrytis cinerea* 241

**DOI:** 10.3390/jof7020143

**Published:** 2021-02-17

**Authors:** Ingrida Radveikienė, Regina Vidžiūnaitė, Rita Meškienė, Rolandas Meškys, Vida Časaitė

**Affiliations:** 1 Life Sciences Center, Department of Bioanalysis, Institute of Biochemistry, Vilnius University, Sauletekio Ave. 7, 10257 Vilnius, Lithuania; regina.vidziunaite@bchi.vu.lt; 2 Life Sciences Center, Department of Molecular Microbiology and Biotechnology, Institute of Biochemistry, Vilnius University, Sauletekio Ave. 7, 10257 Vilnius, Lithuania; rita.meskiene@bchi.vu.lt (R.M.); rolandas.meskys@bchi.vu.lt (R.M.)

**Keywords:** yellow laccase, *Botrytis cinerea*, biocatalysis, dye decolorization

## Abstract

Typical laccases have four copper atoms, which form three different copper centers, of which the T1 copper is responsible for the blue color of the enzyme and gives it a characteristic absorbance around 610 nm. Several laccases have unusual spectral properties and are referred to as yellow or white laccases. Only two yellow laccases from the Ascomycota phylum have been described previously, and only one amino acid sequence of those enzymes is available. A yellow laccase Bcl1 from *Botrytis cinerea* strain 241 has been identified, purified and characterized in this work. The enzyme appears to be a dimer with a molecular mass of 186 kDa. The gene encoding the Bcl1 protein has been cloned, and the sequence analysis shows that the yellow laccase Bcl1 is phylogenetically distinct from other known yellow laccases. In addition, a comparison of amino acid sequences, and 3D modeling shows that the Bcl1 laccase lacks a conservative tyrosine, which is responsible for absorption quenching at 610 nm in another yellow asco-laccase from *Sclerotinia sclerotiorum*. High thermostability, high salt tolerance, broad substrate specificity, and the ability to decolorize dyes without the mediators suggest that the Bcl1 laccase is a potential enzyme for various industrial applications.

## 1. Introduction

Laccases (benzenediol: oxygen oxidoreductase; E.C. 1.10.3.2) oxidize a great variety of aromatic compounds [1,2]. They can be applied for wide industrial applications: organic synthesis, biosensor construction, biofuel cells development, biomass valorization, and xenobiotic biodegradation [3,4,5,6,7,8,9,10,11,12]. Typical laccases have four copper atoms, which form three different copper centers (type T1, T2 and T3) [13,14]. The T1 copper is responsible for the blue color of the enzyme and gives it a characteristic absorbance around 610 nm [15,16,17]. However, several laccases have unusual spectral properties. These enzymes are referred to as “yellow” or “white” laccases [13,18]. Some fungi, such as *Panus tigrinus*, *Phlebia radiata*, and *Phlebia tremellosa* [18], *Stropharia aeruginosa* [19], *Ganoderma fornicatum* [20], *Pleurotus ostreatus* [21], *Leucoagaricus naucinus* [22], *Daedalea flavida* [23], *Trametes* sp. F1635 [24] and *Sclerotinia sclerotiorum* [25] produces yellow laccases. The features of the T1 copper site in yellow or white laccases are missing in their spectra. Different metal ions in active site (such as iron [26] or zinc and iron [27]), incomplete oxidation state of copper [26] or altered copper coordination sphere [28] are associated with the form of white laccase.

Leontievsky and co-authors showed that yellow laccase occurs upon binding of a low molecular mass phenolic compound. Subsequently Mot and co-authors identified the binding site—the compound covalently binds to the tyrosine residue site at the T1 center and turns laccase from blue to yellow [18,29]. Yellow laccases outperform blue laccases, with improved catalytic properties as well as the ability to oxidize some substrates (e.g., nonphenolic part of lignin) without mediators, making them attractive biocatalysts [30,31]. The diversity and catalytic properties of these laccases including ones from *Botrytis cinerea* (teleomorphic form: *Botryotinia fuckeliana*) ascomycetes are still scarcely investigated. Usually, each *Botrytis* spp. strain can produce several laccase isoenzymes of rather different physico-chemical features [32,33,34,35,36,37,38]. Such abundance of laccases produced by these fungi is not surprising, as 15 genes encoding laccases have been identified in the genome of *B. cinerea* [39,40]. Laccases produced by this phytopathogen, which infects at least 1400 plant species, is presumably related to detoxification of defense metabolites produced by the plant. *B. cinerea* laccases destruct plants and impacts phenolic composition and quality of must and wine [41,42,43], however, yellow laccase from *B. cinerea* has not been known so far.

In this study, we purified a yellow laccase Bcl1 of *B. cinerea* 241, identified the Bcl1 protein encoding gene and compared the protein properties with other yellow laccases. In addition, we presented data on biochemical and catalytic properties of the Bcl1 enzyme including the de-colorization of some synthetic dyes.

## 2. Materials and Methods 

### 2.1. Materials

2,2’-azino-bis(3-ethylbenzothiazoline-6-sulfonic acid) diammonium salt (ABTS), promazine hydrochloride, syringaldazine, 1-naphthol, 2-naphthol, vanillin, galangin, kaempferol, myricetin, quercetin, fisetin, gallic, syringic, synapic, cinnamic, vanillic, chlorogenic acids, veratryl alcohol, L-3,4-dihydroxyphenylalanine (L-dopa), metal salts, and hydrogen peroxide were purchased from Sigma-Aldrich, Buchs, Switzerland. Methyl syringate purchased from Lancaster Synthesis, Ward Hill, MA, USA, was additionally recrystallized from ethanol. 2,6-dimethoxyphenol was obtained from Alfa Aesar, Kandel, Germany. Methanol, *p*-coumaric, *o*-coumaric, *m*-coumaric, ferulic, and caffeic acids were obtained from Fluka, Steinheim, Switzerland. *N,N′*-dimethylamine-4-(4-morpholine)benzene, 3-(10*H*-phenoxazin-10-yl)propanoic acid and 2-(10*H*-phenoxazin-10-yl)ethanol were prepared as described [44]. Catechol, hydroquinone, *p*-phenylenediamine, potassium hexacyanoferrate (II), and dyes were obtained from Reachim, Moscow, Russia. Molecular biology enzymes and kits were purchased from Thermo Fisher Scientific, Vilnius, Lithuania.

### 2.2. Media and Culture Conditions

The fungal strains were kept on malt extract agar media (2% of agar, 2% of malt extract and 0.4% of yeast extract). The pre-culture medium consisted of (g L^−1^): malt extract–3.5, yeast extract–2.5, MgSO_4_–0.5, glucose–8.0, KH_2_PO_4_–2.0, pH 5.0. SSL1 medium consisted of (g L^−1^): malt extract–5.0, vegetable peptone–10.0, MgSO_4_–0.1, pH 5.2. CuSO_4_ (1.5 mM) and H_3_PO_4_ (1.0 mM) were added after sterilization. SSL2 medium consisted of (g L^−1^) malt extract–3.5, yeast extract–2.5, KH_2_PO_4_–20.0, MgSO_4_–0.5, pH 5.5. CuSO_4_ (6.0 mM) was added after sterilization.

The mycelia of fungi taken from spoiled raspberries fruits were placed in sterile distilled water, mixed for 2 min and the aliquots were spread on a solid medium supplemented with ABTS (1 mM). The plates were incubated at 20 °C for five days. Microorganisms showing positive reaction were selected and purified by streaking repeatedly on the malt agar medium.

The liquid pre-cultures were prepared by adding 1 cm^2^ of fungal culture from the agar plates to the 50 mL of pre-culture medium and grown for 4 days at 20 °C with agitation on the rotary shaker. The Erlenmeyer flasks with 200 mL of medium SSL1 or SSL2 were inoculated with 8 mL of liquid fungal suspension and cultivated for 3 days on a rotary shaker at 180 rpm and 20 °C.

### 2.3. Purification of Laccases

When the laccase activity had reached its production peak, the culture liquid was collected, and fungal mycelium was removed by centrifugation at 1500× *g* and filtration. The isoenzymes were purified using different schemes. For purification of Bcl1, 10 mL of CM Toyopearl 650 M (ToyoSoda, Tokyo, Japan) was added to the 1 L of culture liquid and stirred for 4–5 h. The sorbent was precipitated by centrifugation, washed with 2 mM sodium citrate buffer, pH 5.5, poured to the column (C 10/40 Column, GE Healthcare, Chicago, IL, USA) and the adsorbed enzyme was eluted with 0–1.0 M (NH_4_)_2_SO_4_ gradient. Fractions with laccase activity were pooled, supplemented with (NH_4_)_2_SO_4_ up to 1.5 M and applied to the PHE FF (GE Healthcare, Chicago, IL, USA) column equilibrated with 2 mM sodium citrate 1.5 M (NH_4_)_2_SO_4_ buffer pH 5.5. Protein was eluted with 1.5–0 M(NH_4_)_2_SO_4_ gradient, fractions with laccase activity were collected, concentrated and dialyzed against 2 mM sodium citrate buffer pH 5.5. For purification of Bcl2, the DEAE Toyopearl 650 M (TojoSoda, Tokyo, Japan) was added to the clarified culture medium (10 mL/L), stirred for 1–2 h and removed by centrifugation. The cleared culture liquid was concentrated by tangent ultrafiltration with 10 kDa cut-off filter (Millipore, Burlington, MA, USA). Up to 2.2 M of (NH_4_)_2_SO_4_ was added to the crude laccase and solution was kept at 4 °C overnight. The precipitates were removed by centrifugation and clear supernatant was applied to a PHE-Sepharose FF column (GE Healthcare, Chicago, IL, USA), equilibrated with 10 mM sodium citrate 2.2 M(NH_4_)_2_SO_4_ buffer, pH 5.5. Retained laccase Bcl2 was eluted by linear gradient of 2.2–0 M (NH_4_)_2_SO_4_ in the 10 mM sodium citrate buffer. Fractions with laccase activity were collected, concentrated, dialyzed against the 5.0 mM sodium citrate buffer, pH 4.6 and applied on the Super Q Toyopearl 650 M column (ToyoSoda, Tokyo, Japan). The laccase was eluted by 0–0.5 M(NH_4_)_2_SO_4_ linear gradient. Fractions with laccase activity were collected, concentrated, supplemented with 2.0 M(NH_4_)_2_SO_4_ and applied on the source PHE15 (GE Healthcare, Chicago, IL, USA) column, equilibrated with 5 mM sodium citrate 2.0 M (NH_4_)_2_SO_4_ buffer, pH 5.5. Protein was eluted with 2.0–0 M (NH_4_)_2_SO_4_ gradient, fractions with laccase activity were collected, concentrated and dialyzed against 2.0 mM sodium citrate buffer pH 5.5. Both purified enzymes were stored at −20 °C.

### 2.4. Characterization of Laccases

The UV-vis absorption spectra of the purified laccases were determined at wavelengths between 260 and 800 nm at 25 °C in a sodium acetate buffer (50 mM, pH 5.5) using a Nicolet evolution 300 (Thermo Electron Corporation, Waltham, MA, USA) spectrophotometer. SDS-PAGE was performed according to the method of Laemmli in 14% polyacrylamide gels by using molecular mass standards (Pierce™ Unstained Protein Molecular Weight Marker and PageRuler™ Prestained Protein Ladder, Thermo Fisher Scientific, Vilnius, Lithuania). Protein concentration was determined by the method of Lowry using bovine serum albumin as a standard [45].

The molecular mass of the purified laccase was estimated by both SDS-PAGE and size exclusion chromatography (SEC) on a SuperdexTM200 5/150 GL (GE Healthcare, Chicago, IL, USA) analytical size exclusion column. The buffer for SEC was 50 mM Tris-HCl supplemented with 150 mM NaCl, pH 8. The molecular mass standards were apoferritin (443 kDa), β-amylase (200 kDa), alcohol dehydrogenase (150 kDa) and albumin (66 kDa), provided by Sigma, Darmstadt, Germany.

For deglycosylation of laccases, 1000 U of endoglycosidase H (New England BioLabs, Ipswich, MA, USA) was added to the purified protein (2 µg), incubated at 37 °C for 4 h and the degree of deglycosylation was assessed by SDS-PAGE.

### 2.5. MS/MS Analysis

After protein separation in SDS-PAGE gel, the laccase corresponding band was excised and subjected to *de novo* sequencing based on matrix-assisted laser desorption ionization–time of flight (MALDI-TOF)/TOF mass spectrometry (MS) and subsequent computational analysis at the Proteomics Centre of the Life Sciences Center, Vilnius University (Vilnius, Lithuania). The sample was purified and the analysis of peptides was performed as described previously [46,47]. 

### 2.6. Gene Cloning and Analysis

*B. cinerea* 241 mycelium from a culture grown in liquid medium was harvested when first laccase activity could be detected (six day of cultivation), and genomic DNA and RNA were isolated using ZR Fungal/Bacterial DNA MiniPrep kit (Zymo Research, Irvine, CA, USA) and Quick-RNA Fungal/Bacterial Miniprep Kit (Zymo Research, Irvine, CA, USA), respectively. cDNA was synthesized by the Maxima H Minus First Strand cDNA Synthesis Kit and oligo dT primer. The primer set, 241LACR (5′-TTARASACCAGARTCGGTCTTGAWAT-3′) and 241LACF (5′-TTSTTSAATATTCTACTTTTRTCTTC-3′), was designed according to *B. cinerea* hypothetical protein BofuT4_P092250.1 (CCD50266.1) and Bclcc7 (XP_001551072.1) gene sequences. The primer set and Phusion Green Hot Start High-Fidelity PCR Master Mix were used to amplify laccase genes using both the chromosomal DNA and cDNA. PCR conditions were as follows: initial denaturation step at 98 °C for 1 min, followed by 30 cycles each consisting of 98 °C for 20 s, 50 °C for 20 s, and 72 °C for 1 min, with the final extension at 72 °C for 5 min. The PCR products were purified with a PCR purification kit and cloned into pTZ57R/T vector. *E. coli* DH5α strain was used for transformation and resulted plasmid was sequenced in both orientations (Macrogene, Amsterdam, Netherlands).

18S rRNA encoding gene was amplified with the primers EukA (5′-AACCTGGTTGATCCTGCCAGT-3′) and EukB (5′-GATCCWTCTGCAGGTTCACCTAC-3′) [48]. ITS region was amplified using the primers ITS5 (5′-GGAAGTAAAAGTCGTAACAAG-3′) and ITS4 (5′-TCCTCCGCTTATTGATATGC-3′) [49]. The PCR condition were the same as for laccase gene cloning. The PCR products were purified and sequenced in both orientations. The nucleotide sequences were deposited in the GenBank under the accession numbers: MH333282, MT704558, MT707622.

BLAST program (https://blast.ncbi.nlm.nih.gov/Blast.cgi) at the National Center for Biotechnology Information (NCBI) was used for the nucleotide sequence analysis, deduction of the amino acid sequence, and database searches [50]. The phylogenetic analysis was performed using MEGA X [51]. The 3D models of laccases were constructed as previously [29] by the use of (PS)^2^-v2: Protein Structure Prediction Server (http://ps2.life.nctu.edu.tw/) [52] using the *Melanocarpus albomyces* laccase 3D structure (pdb code 2Q9O) as template by automatic selection. The 3D structures were compared and visualized by using UCSF Chimera [53].

### 2.7. Enzyme Activity Assays

Routinely, activity of laccase was determined spectrophotometrically using ABTS as a substrate. The 1 mL of reaction mixture consisted of 50 mM sodium acetate buffer, pH 3.0, 0.1 mM ABTS and 5 µL (0.01–0.1 U) of laccase. The ABTS oxidation was monitored by the increase of absorbance at 420 nm (ε = 36 000 M^−1^ cm^−1^) at 25 °C. One unit of enzyme was defined as amount of enzyme that oxidized 1 µmol of ABTS per minute.

Tyrosinase activity was estimated by following the oxidation of 2 mM of L-dopa (L-3,4-dihydroxyphenylalanine) to dopachrome (2-carboxy-2,3-dihydroindole-5,6-quinone) at 475 nm (ε = 37 000 M^−1^cm^−1^) [54].

Peroxidase activity was determined by monitoring the oxidation of ABTS in the presence of 0.1 mM H_2_O_2_ [55]. ABTS oxidation by laccase without H_2_O_2_ was used as a control.

Oxidation of veratryl alcohol (0.1 mM) to veratrylaldehyde was determined as described previously [56] by observing an increase in absorbance at 310 nm after 4, 6 and 24 h.

The oxidation of the selected substrates was monitored spectrophotometrically and spectrofluorometrically in 50 mM sodium acetate buffer solution, pH 5.5, at 25 °C. The kinetic curves were recorded at the wavelength corresponding to the maximum of absorbance. The catalytic constants were determined as described previously [57].

The decreasing fluorescence intensity during oxidation of 1-naphthol and 2-naphthol was registered at 460 nm when excitation was at 320 nm and 328 nm, respectively. The concentration of naphthols was calculated using fluorescence intensity coefficients, determined from the calibration curves.

Laccase activity at different pH value was determined in a pH-range of 3.0–9.0 using a universal 60 mM Britton-Robinson buffer [58]. ABTS, 2,6-dimethoxyphenol, catechol, *p*-phenylenediamine, syringaldazine, and myricetin were used as substrates. For determination of the pH stability, enzyme was incubated at different pH in the range of pH 3.0–7.0, at room temperature for 18 h and the residual activity was determined with ABTS as a substrate. Optimal temperature for the laccase activity was determined by measuring the activity of enzyme at 22–70 °C. For determination of the thermal stability, laccase was incubated at different temperature (30–70 °C) for 10 min and the residual activity was determined with ABTS as a substrate. The time-course of stability of laccase was determined by incubating the enzyme at 60 °C for 10–150 min and measuring the residual enzyme activity. The effect of metal salts on laccases activity was evaluated using ABTS assay and 1–25 mM of CaCl_2_, CoCl_2_, CuCl_2_, KCl, NaCl, MgCl_2_, MnCl_2_, NiCl_2_, CdI, Li_2_SO_4_, ZnSO_4_, and Pb(CH_3_COO)_2_. 

Dye decolorization was detected by measuring the decrease of absorbance of the appropriate dye. Acid blue 45 (595 nm), Indigo carmine (608 nm), Nile blue A (635 nm), Methylene blue (665 nm), Thionine (598 nm), Direct blue 90 (610 nm), Meldola blue (570 nm), Azure B (648 nm), Erythrosine (524 nm), Eriochrome black T (574 nm), and Xylenol blue (435 nm) were incubated with laccase (84 nM of Bcl1) in 50 mM sodium acetate buffer (pH 5.5) at 25 °C for 24 h. Samples without the enzyme were used as controls. Initial concentration of dye solutions was set at OD 0.4–0.8 on their maximal absorbance wavelength. The relative decolorization percentage was calculated as described [59].

## 3. Results

### 3.1. The Indentification and Purification of Laccases

A fungal strain 241 oxidizing ABTS on the solid medium was isolated from a spoiled raspberry. The fungus formed a white fuzzy mycelium, produced black resting structures (sclerotia) and branching tree-like conidiophores with conidia after growth on malt extract agar medium at 20 °C for one week. The isolate was identified as *Botrytis cinerea,* based on morphology and sequences of 18S rRNA and internal transcribed spacer (ITS).

An analysis of extracellular enzymes showed that *B. cinerea* 241 secreted two different laccases into the media. The enzymes were assigned as Bcl1 and Bcl2. Both laccases were produced irrespective of the composition of used media, but in different quantities. Bcl1 was most efficiently produced in the medium SSL1 without additional salts and at lower copper concentration (1.5 mM). On the contrary, production of Bcl2 was increased in the medium SSL2 supplemented with potassium phosphate (20 g L^−1^) and copper (6 mM). The highest laccase activity was detected after seven days of growth (four days of pre-culture and three days of culture) and then decreased in both media studied. The laccases Bcl1 and Bcl2 in SSL1 and SSL2 media reached a maximum activity of 168 U L^−1^ and 4333 U L^−1^, respectively.

Two different schemes were applied for purification of Bcl1 and Bcl2 (Supplementary Table S1). Under optimal growth conditions, production of Bcl1 was ten times lower than Bcl2–0.7 mg L^−1^ and 7 mg L^−1^, respectively. The specific activity of the purified enzyme with ABTS was 76 and 147 U mg^−1^ of protein for Bcl1 and Bcl2, respectively.

**Figure 1:**
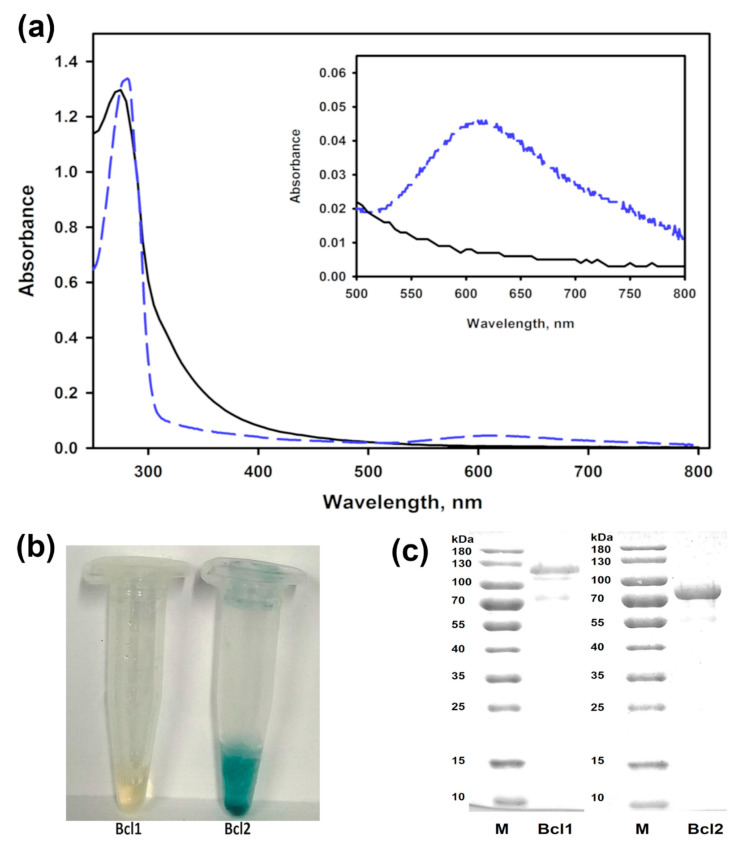
The properties of laccases Bcl1 and Bcl2. (**a**) The UV-VIS spectrum of the purified *Botrytis cinerea* 241 laccase Bcl1 (black solid line) and Bcl2 (blue dashed line). (**b**) The samples of the purified enzymes. (**c**) SDS-PAGE analysis of the purified proteins. M—molecular mass marker.

The solution of the purified Bcl1 had a light-yellow color and no peak near the 600 nm in the UV-VIS spectrum. A shoulder at around 330 nm in this spectrum was also detected for Bcl1 suggesting the presence of a T3 copper site. The laccase Bcl2 exhibited a typical blue color of copper oxidases and an absorbance peak at 610 nm (Figure 1).

The molecular mass determined by SDS-PAGE, was 110 ± 10 kDa and 81 ± 5 kDa for Bcl1 and Bcl2, accordingly. The molecular mass of Bcl1 determined by a size exclusion chromatography was 186 ± 15 kDa (Figure S1), showing that the Bcl1 was likely a homodimer. Deglycosylation of Bcl1 reduced the activity of enzyme by 50%, and the molecular mass from 110 kDa to 104 kDa (Figure S2).

### 3.2. The Identification of the Bcl1 Encoding Gene

For further analysis, the protein band corresponding to the Bcl1 laccase was cut from polyacrylamide gel and, after hydrolysis with trypsin, the formed peptides were analyzed by MS/MS. In total seven peptides were obtained and covered approximately 42% of sequence of the Bclcc7 laccase from *Botrytis cinerea* strain B05.10 (accession number NCBI XP_001551072.1). Based on the detected peptides and the homologous gene sequence, primers were synthesized and used for the cloning of the Bcl1 laccase encoding gene from *Botrytis cinerea* 241. Both the genomic DNA and first-strand cDNA reverse transcribed from mRNA were used for amplification of the desired gene. Two fragments consisted of 2167 bp and 2052 bp were obtained using genomic DNA and cDNA as matrices, respectively. An analysis of the nucleotide sequences allowed identification the ORF encoding a polypeptide of 684 amino acid residues. Comparison of DNA- and mRNA-based sequences of the *bcl1* gene indicated the presence of three exons and two introns of 54 and 59 bp in length (accession No. MT707622). Both intron splice junctions corresponded to the GT-AG rule.

**Figure 2:**
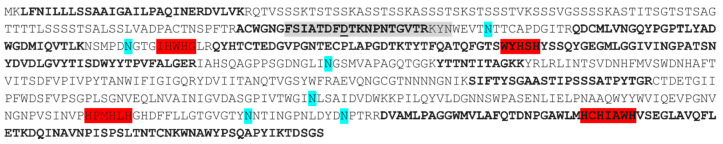
The amino acid sequence of the Bcl1 laccase. The peptides detected by the MS/MS are marked in bold; the amino acids involved in the coordination sites for copper of the type 1, 2 and 3 are highlighted in red; putative Asn-Xaa-Ser/Thr glycosylation sites are highlighted in blue; and the peptide of a flexible loop, homologous to the *Sclerotinia sclerotiorum* laccase motif containing Y65 [29], that is involved in binding of phenolic compounds, is highlighted in grey (the corresponding amino acid residue D132 in Bcl1 is underlined).

The analysis of amino acid sequence of the Bcl1 protein revealed three similar cupredoxin domains. Ten conserved His and one Cys, required to coordinate four copper atoms at the three typical Cu(II)-type centers, were presented in the deduced amino acid sequence of the cloned Bcl1 laccase (Figure 2).

**Figure 3:**
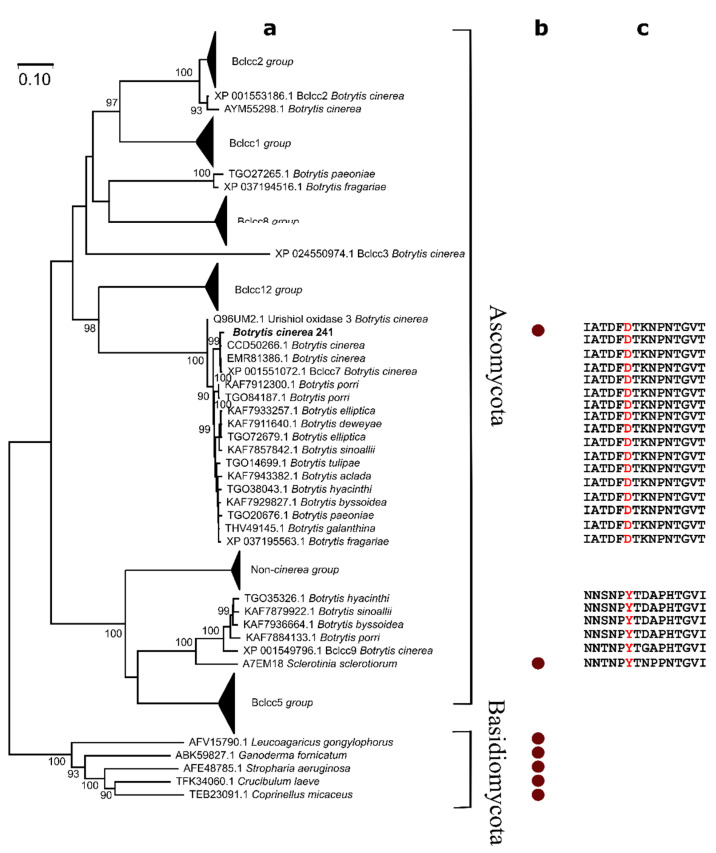
(**a**) The phylogenetic analysis of laccases encoded by *Botrytis* spp. The evolutionary history was inferred using the Neighbor-Joining method [60]. Scale bar: 10 substitutions site^−1^. The phylogenetic analysis was performed using MEGA X [51]. Bclcc numeration is based on laccases from *Botrytis cinerea* strain B05.10 (accession number NCBI XP_001551072.1). A yellow laccase from *Botrytis cinerea* 241 is marked by a bold text. The sequences used for phylogenetic analysis are listed in Table S2. (**b**) Known yellow laccases are included in the analysis, and are marked by circle, respectively. (**c**) Amino acid sequences of a flexible loop, which is homologous to the *Sclerotinia sclerotiorum* laccase motif containing Y65, that is involved in binding of phenolic compounds. Y65 and the corresponding amino acid residue D132 in Bcl1 are highlighted in red.

A phylogenetic analysis was carried out to study the relationship of the Bcl1 laccase and laccases from *Botrytis* genus as well as previously characterized yellow laccases. The analysis clearly differentiated two distinct groups containing the laccases from fungi of Basidiomycota and Ascomycota phyla (Figure 3). The internal arrangement within the *Botrytis* group consisted of 11 different branches. The sequence of the Bcl1 protein clustered with the sequence of Bclcc7 of *Botrytis cinerea* B05.10. In contrast, the yellow laccase from *Sclerotinia sclerotiorum* (the only one ascomycotal yellow laccase with a known sequence) formed an independent branch together with the Bclcc9 laccase from *Botrytis cinerea* B05.10 (Figure 3). In addition, it was found, that a sequence of a flexible loop analogous to the *Sclerotinia sclerotiorum* laccase motif involved in binding of phenolic compounds [29] was very conservative in the proteins forming the Bcl1 branch (Figure 3c). Moreover, this loop contained aspartic acid instead of the conserved tyrosine, found in the yellow laccase from *Sclerotinia sclerotiorum* and all laccases of the Bclcc9-related group of proteins (Figure 3c).

### 3.3. Catalytic Properties of the Laccase Bcl1

Bcl1 oxidized veratryl alcohol (a typical substrate for yellow laccases) (Figure S3) and most of the studied non-phenolic and phenolic compounds such as flavonoids, hydroxycinnamic and hydroxybenzoic acids. The kinetic parameters of Bcl1 are presented in Table 1.

**Figure 4:**
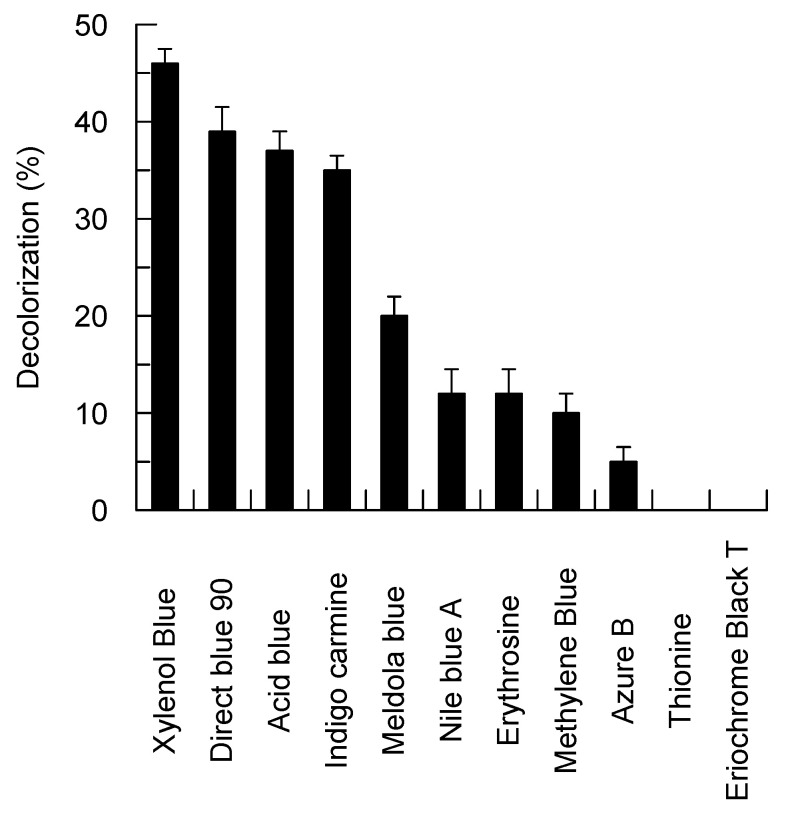
Decolorization of the synthetic dyes by the Bcl1 laccase.

In addition, Bcl1 was able to oxidize nine structurally different dyes including phenothiazines and indigoids without the presence of any redox mediator (Figure 4). However, Bcl1 did not oxidized vanillin, 2-naphthol, galangin, cinnamic, vanillic, *m*-, *o*-, *p*-coumaric acids, and tyrosine. Moreover, Bcl1 was not active towards hydrogen peroxide.

**Figure 5:**
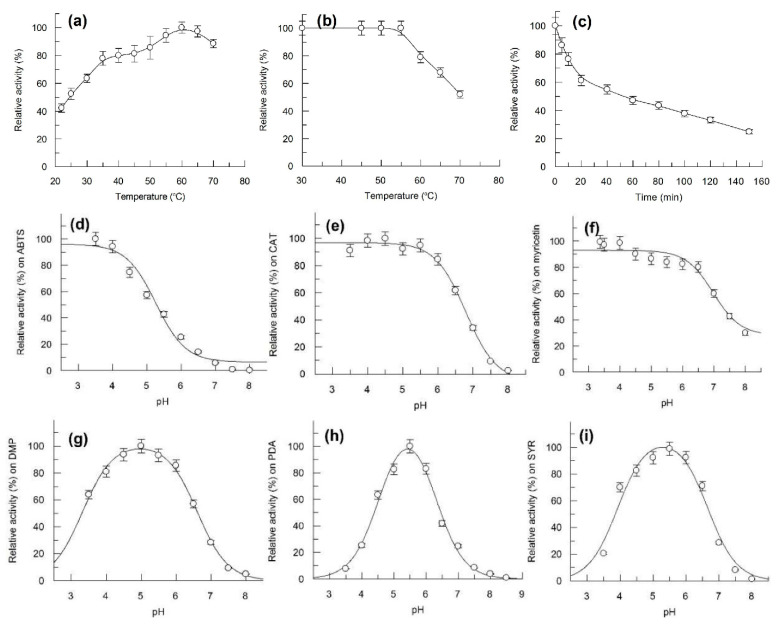
Effect of temperature on activity and stability of Bcl1 laccase and effect of pH on Bcl1 laccase activity. (**a**) activity of Bcl1 at different temperatures; (**b**) residual activity of Bcl1 incubated for 10 min at different temperature; (**c**) residual activity of Bcl1 at 60 °C, incubated for 0–140 min. (**d–i**) activity of Bcl1 at different pH; (**d**) ABTS, (**e**) catechol (CAT), (**f**) myricetin, (**g**) 2,6-dimethoxyphenol (DMP), (**h**) *p*-phenylenediamine (PDA) and (**i**) syringaldazine (SYR) were used as substrates in 60 mM Britton-Robinson buffer.

The optimum temperature for Bcl1 activity was identified as 60 °C (Figure 5a). The enzyme was stable at 30–55 °C, half of the activity was lost after incubation for 10 min and 60 min at 70 °C and at 60 °C, respectively (Figure 5b,c). However, approximately 60% of activity could be detected after 30 days of storage at 4 °C and 22 °C.

Bcl1 was stable between pH 3.0–7.0 and showed a full activity towards ABTS for 18 h (data not shown). Bcl1 was active at an acidic pH on ABTS, catechol, and myricetin, activity decreased with increasing pH. The enzyme activity decreased at pH values below 4 or above 6 on 2,6-dimethoxyphenol, *p*-phenylenediamine and syringaldazine. (Figure 5). No activity was detected above pH 8.0.

The Bcl1 laccase demonstrated a high tolerance towards ionic strength of buffer (I_50_–1.6 M NaCl) and showed slightly reduced activity in the presence of various cations (up to 25 mM) (Figure S4), but the enzymatic activity was completely inhibited by sodium azide.

## 4. Discussion

Depending on the growth conditions, *Botrytis cinerea* 241 strain produces two different laccases (Bcl1 and Bcl2). Production of more than one laccase by the same fungal strain is typical and has been reported previously [33,61]. Laccase isoenzymes have been found to originate from the same or different genes [62]. For example, in total, 17 laccase encoding genes have been identified in *Coprinopsis cinerea*, and at least nine of them are functional products [63]. An analysis of *Botrytis cinerea* B05.10 genome revealed 15 putative laccase genes [40]. One of the identified and purified enzymes in this work–Bcl1–shows a UV-VIS spectrum related to yellow laccases. To our best knowledge, only two yellow laccases from the Ascomycota phylum have been described previously–one from *Gaeumannomyces graminis* [64] and another from *Sclerotinia sclerotiorum* [29]. Furthermore, an amino acid sequence is available for the protein from *Sclerotinia sclerotiorum* only, hence, the Bcl1 protein has been chosen for detailed studies.

The molecular masses of the previously characterized blue laccases from *Botrytis* spp. varies from 60 kDa to 100 kDa [33,34,35,65]. The molecular mass of the Bcl1 protein determined by SDS-PAGE is 110 kDa, however, the theoretical mass based on amino acid sequence is 73.4 kDa only. By this feature, Bcl1 is the most similar to *Botrytis aclada* laccase [65], that migrates in the SDS-PAGE as 100 kDa protein, although the theoretical molecular mass is 61 kDa; in contrast to the monomeric *Botrytis aclada* laccase, the Bcl1 enzyme is a homodimer. It can be proposed that a ~37 kDa difference in molecular masses is due to glycosylation of Bcl1. The sequence analysis according [66] shows seven potential *N*-glycosylation sites (Asn–Xxx–Ser/Thr) in Bcl1, at positions 150, 194, 308, 339, 484, 565, and 577 of the deduced protein (Figure 2). However, deglycosylation of the purified protein reduces the molecular mass of the treated protein by ~6 kDa only (Figure S2). Hence, other reasons such as an unsuitable substrate specificity of the endoglycosidase H or an unknown post-synthetic modification take place.

The sequence of Bcl1 is highly similar (differs by five amino acid residues only) to the Bclcc7 protein from *Botrytis cinerea* B05.10. According to the published data, the *bclcc7* gene is functional and the corresponding laccase has been detected in the vineyards and botrytized wines [36,38,67], however, the Bclcc7 enzyme has not been purified and characterized previously. In addition, a phylogenetic analysis shows, that Bcl1 and *Sclerotinia sclerotiorum* laccase has a low similarity (38% of identical amino acids) and are located on two different phylogenetic branches (Figure 3). The *Sclerotinia sclerotiorum* laccase contains a tyrosine residue (Y65) that is covalently modified with a phenolic compound or substrate. It has been proposed that such modification is a basis for the yellow color of the enzyme [29]. However, the appropriate tyrosine is absent in the loop near the active center of Bcl1 according to the sequence analysis (Figure 2 and Figure 3) and the modelling of the protein structure (Figure S5). This suggests that other mechanisms responsible for absorption quenching at 600 nm may take place, and additional studies are needed to elucidate how the spectral properties are determined in the Bcl1 protein.

Bcl1 shows a wide substrate specificity and is active towards various naturally occurring and synthetic non-phenolic as well as phenolic substrates. The purified Bcl1 effectively oxidizes flavonoids, such as myricetin, fisetin and chlorogenic acid. Given that *Botrytis cinerea* is a plant pathogen, it can be assumed that Bcl1 is involved in the neutralization of plant defense mechanisms. The kinetic parameters of Bcl1 are similar to those of yellow laccases from *Trametes* sp. F1635 [24] and *Daedalea flavida* MTCC-145 [23]. The Bcl1 laccase tolerates high concentration of NaCl, a similar effect has been observed for laccases from *Paraconiothyrium variabile* [68] and *Botrytis aclada* [65]. From a practical point of view, such property provides strong advantages for various environmental applications. In addition, the Bcl1 laccase decolorizes different dyes without the presence of mediators. Even the hardly degradable dye Direct blue 90, whose molecule consists of eight aromatic rings, has been used by Bcl1 laccase as a substrate. The mechanism of laccase-catalyzed dye decomposition is different depending on dye structures [69,70,71,72]. For example, the anthraquinone-type dyes can be an enzyme substrate that is directly oxidized by laccase while decolorization of azo- (e.g., analogous to Direct blue 90) and indigo-type dyes requires involvement of some molecules–mediators [69]. In the latter case, the decolorization rate of the nonsubstrate dyes is limited by the concentration of mediating compounds rather than laccase activity in the solutions [72]. Both natural and synthetic compounds including ABTS, *p*-coumaric acid, 3-hydroxyanthranilate, 1-hydroxybenzotriazole, syringaldehyde, 2,2,6,6-tetramethylpiperidine-*N*-oxyl, and violuric acid can mediate decolorization of synthetic dyes by laccases [70,72]. It is known that yellow laccases can outperform blue laccases in the ability to oxidize some complex substrates without mediators, making them attractive biocatalysts [30,31] There have been reports of substrate activation for yellow laccases. Hence, Mot et al. [29] identified a biphasic behavior in the Michaelis–Menten saturation curve of the *Sclerotinia sclerotiorum* yellow laccase and associated a high-affinity phase (characterized by a lower Km) with the formation of an adduct of tyrosine Y65 residing in the flexible loop with the electron-donor substrate such as 2,3-dimethoxy-5-methyl-*p*-benzoquinone, ABTS, and guaiacol. It has been suggested that adduct formation with a substrate enhances the catalytic properties of the yellow laccase [29]. However, due to a lack of the appropriate tyrosine residue a different mechanism should be considered in the case of the Bcl1 laccase.

## 5. Conclusions

In conclusion, the yellow laccase Bcl1 described in this work is phylogenetically distinct from other known yellow laccases. High thermostability, high salt tolerance, broad substrate specificity, and the ability to decolorize dyes without the mediators suggest that the Bcl1 laccase is a potential enzyme for different industrial applications.

## Figures and Tables

**Table 1:** Kinetic parameters of the purified laccase Bcl1.

Substrate	Km, μM	kcat, s^−1^	kcat/Km, M^−1^s^−1^
Non-phenolic compounds			
Potassium hexacyanoferrate (II)	11,000 ± 1000	28.0 ± 1	2.5 ± 0.1 × 10^3^
*p*-Phenylenediamine	150.0 ± 20	15.0 ± 1	1.0 ± 0.02 × 10^5^
Promazine hydrochloride	60.0 ± 10	7.1 ± 0.3	1.2 ± 0.1 × 10^5^
*N,N’*-Dimethylamine-4-(4-morpholine)benzene	180.0 ± 46	31.0 ± 2	1.7 ± 0.3 × 10^5^
2-(10*H*-phenoxazin-10-yl)ethanol	40.0 ± 7	25.0 ± 2	6.3 ± 0.3 × 10^5^
ABTS	13.8 ± 0.2	29.0 ± 1	2.1 ± 0.1 × 10^6^
3-(10*H*-phenoxazin-10-yl)propanoic acid	6.0 ± 0.8	33.0 ± 1	5.5 ± 0.8 × 10^6^
Syringaldazine	0.7 ± 0.1	30.0 ± 2	4.3 ± 0.7 × 10^7^
Phenolic compounds			
Methyl syringate	nd ^1^	nd	3.3 ± 0.2 × 10^3^
Syringic acid	74.0 ± 12	3.4 ± 0.5	4.6 ± 0.5 × 10^4^
2,6-Dimethoxyphenol	9.0 ± 1	8.4 ± 0.1	9.3 ± 0.1 × 10^4^
Hydroquinone	220.0 ± 14	21.4 ± 0.3	9.7 ± 0.2 × 10^4^
Ferulic acid	51.0 ± 3	6.0 ± 0.5	1.2 ±0.05 × 10^5^
Catechol	70.0 ± 4	28.0 ± 1	4.0 ± 0.2 × 10^5^
Gallic acid	27.0 ± 8	20.0 ± 3	7.4 ± 2 × 10^5^
Caffeic acid	15.0 ± 1	13.0 ± 1	8.7 ± 2 × 10^5^
Synapic acid	60.0 ± 10	100.0 ± 10	1.7 ± 0.2 × 10^6^
Quercetin	3.7 ± 0.8	6.7 ± 0.4	1.8 ± 0.3 × 10^6^
Kaempferol	8.6 ± 0.1	17.0 ± 1	2.0 ± 0.4 × 10^6^
Chlorogenic acid	2.0 ± 0.4	10.0 ± 1	5.0 ± 1.0 × 10^6^
Fisetin	1.5 ± 0.1	7.8 ± 0.3	5.2 ± 0.9 × 10^6^
1-Naphthol	20.0 ± 7.0	440.0 ± 20.0	2.2 ± 0.1 × 10^7^
Myricetin	0.7 ± 0.1	17.0 ± 1.0	2.4 ± 0.4 × 10^7^

^1^—not determined. Km and Vmax constants cannot be determined because a linear dependence on the substrate concentration was obtained. That making it possible to calculate only one parameter (kcat/Km =Vo /[S] [E] = slope/[E]).

## Data Availability

Data are contained within the article or Supplementary Materials.

## Supplementary Materials

The following are available online at https://www.mdpi.com/2309-608X/7/2/143/s1, Figure S1: Analytical gel filtration chromatography of Bcl1 and Bcl2, Figure S2: Deglycosylation of Bcl1, Figure S3: Oxidation of veratryl alcohol by the Bcl1 and Bcl2 laccase, Figure S4: Effect of metal ions on the Bcl1 activity, Figure S5: Comparison of 3D models of the laccases from *Botrytis cinerea* 241 and *Sclerotinia sclerotiorum,* Table S1: Purification of laccases Bcl1 and Bcl2 from a 3 L of culture, Table S2: The list of amino acid sequences used for the phylogenetic analysis.

## References

[1] Baldrian P. (2006). Fungal laccases-occurrence and properties. FEMS Microbiol. Rev..

[2] Janusz G., Pawlik A., Świderska-Burek U., Polak J., Sulej J., Jarosz-Wilkołazka A., Paszczyński A. (2020). Laccase properties, physiological functions, and evolution. Int. J. Mol. Sci..

[3] Canas A.I., Camarero S. (2010). Laccases and their natural mediators: Biotechnological tools for sustainable eco-friendly processes. Biotechnol. Adv..

[4] Romero-Guido C., Baez A., Torres E. (2018). Dioxygen activation by laccases: Green chemistry for fine chemical synthesis. Catalysts.

[5] Zerva A., Simić S., Topakas E., Nikodinovic-Runic J. (2019). Applications of microbial laccases: Patent review of the past decade (2009–2019). Catalysts.

[6] Marcinkevičiene L., Vidžiunaite R., Tauraite D., Rutkiene R., Bachmatova I., Morkunas M., Razumiene J., Časaite V., Meškiene R., Kulys J. (2013). Characterization of laccase from *Coriolopsis byrsina* GRB13 and application of the enzyme for synthesis of redox mediators. Chemija.

[7] Laurinavičius V., Kurtinaitienė B., Liauksminas V., Jankauskaitė A., Šimkus R., Meškys R., Boguslavsky L., Skotheim T., Tanenbaum S. (2000). Reagentless biosensor based on PQQ-dependent glucose dehydrogenase and partially hydrolyzed polyarbutin. Talanta.

[8] Dagys M., Laurynenas A., Ratautas D., Kulys J., Vidžiunaite R., Talaikis M., Niaura G., Marcinkevičiene L., Meškys R., Shleev S. (2017). Oxygen electroreduction catalysed by laccase wired to gold nanoparticles via the trinuclear copper cluster. Energy Environ. Sci..

[9] Ratautas D., Ramonas E., Marcinkevičienė L., Meškys R., Kulys J. (2018). Wiring gold nanoparticles and redox enzymes: A self-sufficient nanocatalyst for the direct oxidation of carbohydrates with molecular oxygen. ChemCatChem.

[10] Munk L., Sitarz A.K., Kalyani D.C., Mikkelsen J.D., Meyer A.S. (2015). Can laccases catalyze bond cleavage in lignin?. Biotechnol. Adv..

[11] Arregui L., Ayala M., Gómez-Gil X., Gutiérrez-Soto G., Hernández-Luna C.E., de los Santos M.H., Levin L., Rojo-Domínguez A., Romero-Martínez D., Saparrat M.C.N. (2019). Laccases: Structure, function, and potential application in water bioremediation. Microb. Cell Fact..

[12] Bassanini I., Ferrandi E.E., Riva S., Monti D. (2021). Biocatalysis with laccases: An updated overview. Catalysts.

[13] Giardina P., Faraco V., Pezzella C., Piscitelli A., Vanhulle S., Sannia G. (2010). Laccases: A never-ending story. Cell. Mol. Life Sci..

[14] Solomon E., Heppner D., Johnston E., Ginsbach J., Cirera J., Qayyum M., Kieber-Emmons M., Kjaergaard C., Hadt R., Li T. (2014). Copper active sites in biology. Chem. Rev..

[15] Dwivedi U.N., Singh P., Pandey V.P., Kumar A. (2011). Structure-function relationship among bacterial, fungal and plant laccases. J. Mol. Catal. B Enzym..

[16] Rivera-Hoyos C.M., Morales-Álvarez E.D., Poutou-Piñales R.A., Pedroza-Rodríguez A.M., RodrÍguez-Vázquez R., Delgado-Boada J.M. (2013). Fungal laccases. Fungal Biol. Rev..

[17] Komori H., Higuchi Y. (2015). Structural insights into the O-2 reduction mechanism of multicopper oxidase. J. Biochem..

[18] Leontievsky A.A., Vares T., Lankinen P., Shergill J.K., Pozdnyakova N.N., Myasoedova N.M., Kalkkinen N., Golovleva L.A., Cammack R., Thurston C.F. (1997). Blue and yellow laccases of ligninolytic fungi. FEMS Microbiol. Lett..

[19] Daroch M., Houghton C.A., Moore J.K., Wilkinson M.C., Carnell A.J., Bates A.D., Iwanejko L.A. (2014). Glycosylated yellow laccases of the basidiomycete *Stropharia aeruginosa*. Enzyme Microb. Technol..

[20] Huang W.T., Tai R., Hseu R.S., Huang C.T. (2011). Overexpression and characterization of a thermostable, pH-stable and organic solvent-tolerant *Ganoderma fornicatum* laccase in *Pichia pastoris*. Process Biochem..

[21] Pozdnyakova N.N., Rodakiewicz-Nowak J., Turkovskaya O.V. (2004). Catalytic properties of yellow laccase from *Pleurotus ostreatus* D1. J. Mol. Catal. B Enzym..

[22] Ning Y.J., Wang S.S., Chen Q.J., Ling Z.R., Wang S.N., Wang W.P., Zhang G.Q., Zhu M.J. (2016). An extracellular yellow laccase with potent dye decolorizing ability from the fungus *Leucoagaricus naucinus* LAC-04. Int. J. Biol. Macromol..

[23] Sharma M., Chaurasia P.K., Yadav A., Yadav R.S.S., Yadava S., Yadav K.D.S. (2016). Purification and characterization of a thermally stable yellow laccase from *Daedalea flavida* MTCC-145 with higher catalytic performance towards selective synthesis of substituted benzaldehydes. Russ. J. Bioorg. Chem..

[24] Wang S.N., Chen Q.J., Zhu M.J., Xue F.Y., Li W.C., Zhao T.J., Li G.D., Zhang G.Q. (2018). An extracellular yellow laccase from white rot fungus *Trametes* sp. F1635 and its mediator systems for dye decolorization. Biochimie.

[25] Moţ A.C., Pârvu M., Damian G., Irimie F.D., Darula Z., Medzihradszky K.F., Brem B., Silaghi-Dumitrescu R. (2012). A “yellow” laccase with “blue” spectroscopic features, from *Sclerotinia sclerotiorum*. Process Biochem..

[26] Zhao D., Zhang X., Cui D., Zhao M. (2012). Characterisation of a novel white laccase from the deuteromycete fungus *Myrothecium verrucaria* NF-05 and its decolourisation of dyes. PLoS ONE.

[27] Palmieri G., Giardina P., Bianco C., Scaloni A., Capasso A., Sannia G. (1997). A novel white laccase from *Pleurotus ostreatus*. J. Biol. Chem..

[28] Mate D.M., Garcia-Ruiz E., Camarero S., Shubin V.V., Falk M., Shleev S., Ballesteros A.O., Alcalde M. (2013). Switching from blue to yellow: Altering the spectral properties of a high redox potential laccase by directed evolution. Biocatal. Biotransform..

[29] Mot A.C., Coman C., Hadade N., Damian G., Silaghi-Dumitrescu R., Heering H. (2020). “Yellow” laccase from *Sclerotinia sclerotiorum* is a blue laccase that enhances its substrate affinity by forming a reversible tyrosyl-product adduct. PLoS ONE.

[30] Murugesan K., Nam I.H., Kim Y.M., Chang Y.S. (2007). Decolorization of reactive dyes by a thermostable laccase produced by *Ganoderma lucidum* in solid state culture. Enzyme Microb. Technol..

[31] Murugesan K., Yang I.H., Kim Y.M., Jeon J.R., Chang Y.S. (2009). Enhanced transformation of malachite green by laccase of *Ganoderma lucidum* in the presence of natural phenolic compounds. Appl. Microbiol. Biotechnol..

[32] Mayer A.M., Marbach I., Marbach A., Sharon A. (1977). Amino acid composition and molecular weight of *Botrytis cinerea* laccase. Phytochemistry.

[33] Marbach I., Harel E., Mayer A.M. (1985). Pectin, a second inducer for laccase production by *Botrytis cinerea*. Phytochemistry.

[34] Zouari N., Romette J.L., Thomas D. (1987). Purification and properties of two laccase isoenzymes produced by *Botrytis cinerea*. Appl. Biochem. Biotechnol..

[35] Slomczynski D., Nakas J.P., Tanenbaum S.W. (1995). Production and characterization of laccase from *Botrytis cinerea* 61-34. Appl. Environ. Microbiol..

[36] Schouten A., Wagemakers L., Stefanato F.L., van der Kaaij R.M., van Kan J.A.L. (2002). Resveratrol acts as a natural profungicide and induces self-intoxication by a specific laccase. Mol. Microbiol..

[37] Claus H., Sabel A., König H., Rayess Y.E. (2014). Wine phenols and laccase: An ambivalent relationship. Wine, Phenolic Composition, Classification and Health Benefits.

[38] Quijada-Morin N., Garcia F., Lambert K., Walker A.S., Tiers L., Viaud M., Sauvage F.X., Hirtz C., Saucier C. (2018). Strain effect on extracellular laccase activities from *Botrytis cinerea*. Aust. J. Grape Wine Res..

[39] Amselem J., Cuomo C.A., van Kan J.A., Viaud M., Benito E.P., Couloux A., Coutinho P.M., de Vries R.P., Dyer P.S., Fillinger S. (2011). Genomic analysis of the necrotrophic fungal pathogens *Sclerotinia sclerotiorum* and *Botrytis cinerea*. PLoS Genet..

[40] Van Kan J.A.L., Stassen J.H.M., Mosbach A., Van der Lee T.A.J., Faino L., Farmer A.D., Papasotiriou D.G., Zhou S.G., Seidl M.F., Cottam E. (2017). A gapless genome sequence of the fungus *Botrytis cinerea*. Mol. Plant Pathol..

[41] Bar Nun N., Tal Lev A., Harel E., Mayer A.M. (1988). Repression of laccase formation in *Botrytis cinerea* and its possible relation to phytopathogenicity. Phytochemistry.

[42] Mayer A.M., Staples R.C. (2002). Laccase: New functions for an old enzyme. Phytochemistry.

[43] Mayer A.M. (2006). Polyphenol oxidases in plants and fungi: Going places? A review. Phytochemistry.

[44] Kulys J., Vidziunaite R., Janciene R., Palaima A. (2006). Spectroelectrochemical study of N-substituted phenoxazines as electrochemical labels of biomolecules. Electroanalysis.

[45] Lowry O.H., Rosebrough N.J., Farr L.A., Randall R.J. (1951). Protein measurement with the Folin phenol reagent. J. Biol. Chem..

[46] Hellman U., Wernstedt C., Góñez J., Heldin C.H. (1995). Improvement of an “In-Gel” digestion procedure for the micropreparation of internal protein fragments for amino acid sequencing. Anal. Biochem..

[47] Kutanovas S., Stankeviciute J., Urbelis G., Tauraite D., Rutkiene R., Meskys R. (2013). Identification and characterization of a tetramethylpyrazine catabolic pathway in *Rhodococcus jostii* TMP1. Appl. Environ. Microbiol..

[48] Handy S.M., Demir E., Hutchins D.A., Portune K.J., Whereat E.B., Hare C.E., Rose J.M., Warner M., Farestad M., Cary S.C. (2008). Using quantitative real-time PCR to study competition and community dynamics among Delaware Inland Bays harmful algae in field and laboratory studies. Harmful Algae.

[49] Rédou V., Navarri M., Meslet-Cladière L., Barbier G., Burgaud G. (2015). Species richness and adaptation of marine fungi from deep-subseafloor sediments. Appl. Environ. Microbiol..

[50] Altschul S.F., Gish W., Miller W., Myers E.W., Lipman D.J. (1990). Basic local alignment search tool. J. Mol. Biol..

[51] Kumar S., Stecher G., Li M., Knyaz C., Tamura K. (2018). MEGA X: Molecular Evolutionary Genetics Analysis across computing platforms. Mol. Biol. Evol..

[52] Chen C.-C., Hwang J.-K., Yang J.-M. (2009). (PS)^2^-v2: Template-based protein structure prediction server. BMC Bioinform..

[53] Pettersen E.F., Goddard T.D., Huang C.C., Couch G.S., Greenblatt D.M., Meng E.C., Ferrin T.E. (2004). UCSF Chimera--a visualization system for exploratory research and analysis. J. Comput. Chem..

[54] Horowitz N.H., Fling M., Horn G. (1970). Tyrosinase (*Neurospora crassa*). Meth. Enzymol..

[55] Heinzkill M., Bech L., Halkier T., Schneider P., Anke T. (1998). Characterization of laccases and peroxidases from wood-rotting fungi (family *Coprinaceae*). Appl. Environ. Microbiol..

[56] Sahay R., Yadav R.S.S., Yadava S., Yadav K.D.S. (2012). A laccase of *Fomes durissimus* MTCC-1173 and its role in the conversion of methylbenzene to benzaldehyde. Appl. Biochem. Biotechnol..

[57] Radveikienė I., Pilotaitė I., Dainytė R., Vidžiūnaitė R. (2020). Biosynthesis, purification, characterization and immobilization of laccase from *Lithothelium* sp.. Chemija.

[58] Michałowska-Kaczmarczyk A.M., Michałowski T. (2015). Dynamic buffer capacity in acid-base systems. J. Solution Chem..

[59] Abd El-Rahim W.M., Moawad H., Abdel Azeiz A.Z., Sadowsky M.J. (2017). Optimization of conditions for decolorization of azo-based textile dyes by multiple fungal species. J. Biotechnol..

[60] Saitou N., Nei M. (1987). The neighbor-joining method: A new method for reconstructing phylogenetic trees. Mol. Biol. Evol..

[61] Gigi O., Marbach I., Mayer A.M. (1980). Induction of laccase formation in *Botrytis*. Phytochemistry.

[62] Archibald F.S., Bourbonnais R., Jurasek L., Paice M.G., Reid I.D. (1997). Kraft pulp bleaching and delignification by *Trametes versicolor*. J. Biotechnol..

[63] Kilaru S., Hoegger P.J., Kües U. (2006). The laccase multi-gene family in *Coprinopsis cinerea* has seventeen different members that divide into two distinct subfamilies. Curr. Genet..

[64] Edens W.A., Goins T.Q., Dooley D., Henson J.M. (1999). Purification and characterization of a secreted laccase of *Gaeumannomyces graminis* var. *tritici*. Appl. Environ. Microbiol..

[65] Kittl R., Mueangtoom K., Gonaus C., Khazaneh S.T., Sygmund C., Haltrich D., Ludwig R. (2012). A chloride tolerant laccase from the plant pathogen ascomycete *Botrytis aclada* expressed at high levels in *Pichia pastoris*. J. Biotechnol..

[66] Gavel Y., von Heijne G. (1990). Sequence differences between glycosylated and non-glycosylated Asn-X-Thr/Ser acceptor sites: Implications for protein engineering. Protein Eng. Des. Sel..

[67] Ployon S., Attina A., Vialaret J., Walker A.S., Hirtz C., Saucier C. (2020). Laccases 2 & 3 as biomarkers of *Botrytis cinerea* infection in sweet white wines. Food Chem..

[68] Forootanfar H., Faramarzi M.A., Shahverdi A.R., Yazdi M.T. (2011). Purification and biochemical characterization of extracellular laccase from the ascomycete *Paraconiothyrium variabile*. Bioresour. Technol..

[69] Wong Y., Yu J. (1999). Laccase-catalyzed decolorization of synthetic dyes. Water Res..

[70] Majeau J.A., Brar S.K., Tyagi R.D. (2010). Laccases for removal of recalcitrant and emerging pollutants. Bioresour. Technol..

[71] Bilal M., Rasheed T., Nabeel F., Iqbal H.M.N., Zhao Y. (2019). Hazardous contaminants in the environment and their laccase-assisted degradation–A review. J. Environ. Manag..

[72] Claus H., Faber G., König H. (2002). Redox-mediated decolorization of synthetic dyes by fungal laccases. Appl. Microbiol. Biotechnol..

