# Supplementary material for: Characterization of a Yellow Laccase from Botrytis cinerea 241
Source: J Fungi (Basel). 2021 Feb 17;7(2):143. doi: 10.3390/jof7020143 (PMC7922139; doi:10.3390/jof7020143)
Supplement: Supplementary file 1 [file jof-07-00143-s001.pdf]

Supplementary material

Characterization of a yellow laccase from *Botrytis cinerea* 241

Ingrida Radveikienė[1,*], Regina Vidžiūnaitė[1], Rita Meškienė[2], Rolandas Meškys[2], Vida Časaitė[2,*]

[1] Department of Bioanalysis, Institute of Biochemistry, Life Sciences Center, Vilnius University, Sauletekio av. 7, Vilnius 10257, Lithuania; R. V.: regina.vidziunaite@bchi.vu.lt.

[2] Department of Molecular Microbiology and Biotechnology, Institute of Biochemistry, Life Sciences Center, Vilnius University, Sauletekio ave. 7, Vilnius 10257, Lithuania; Ri.M.: rita.meskiene@bchi.vu.lt, Ro.M.: rolandas.meskys@bchi.vu.lt.

* Correspondence: I.R.: ingrida.jurkeviciute@gmc.vu.lt, V.C.: vida.casaite@bchi.vu.lt

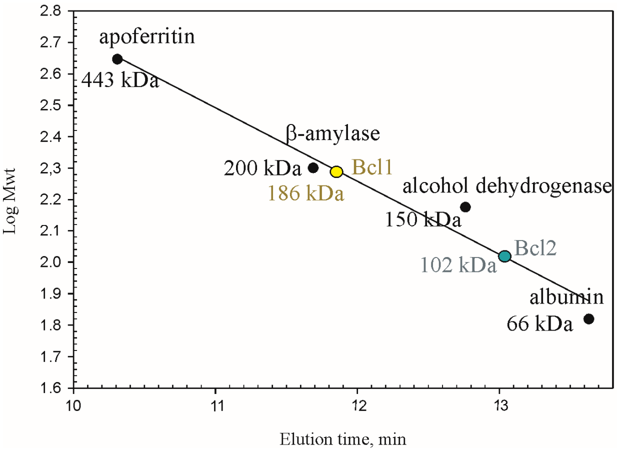

Figure S1. Analytical gel filtration chromatography of Bcl1 and Bcl2. The calibration curve used to estimate the native molecular mass based on the elution position during analytical gel filtration is indicated.

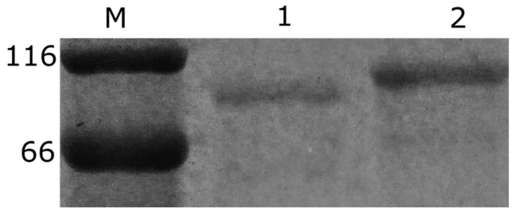

Figure S2. Deglycosylation of Bcl1. SDS-PAGE analysis of proteins after (1) and before (2) deglycosylation.

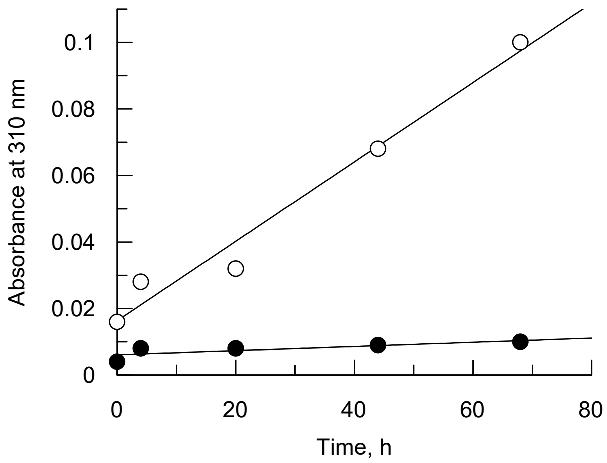

Figure S3. Oxidation of veratryl alcohol by the Bcl1 (empty circle, ○) and Bcl2 (full circle, ●) laccase.

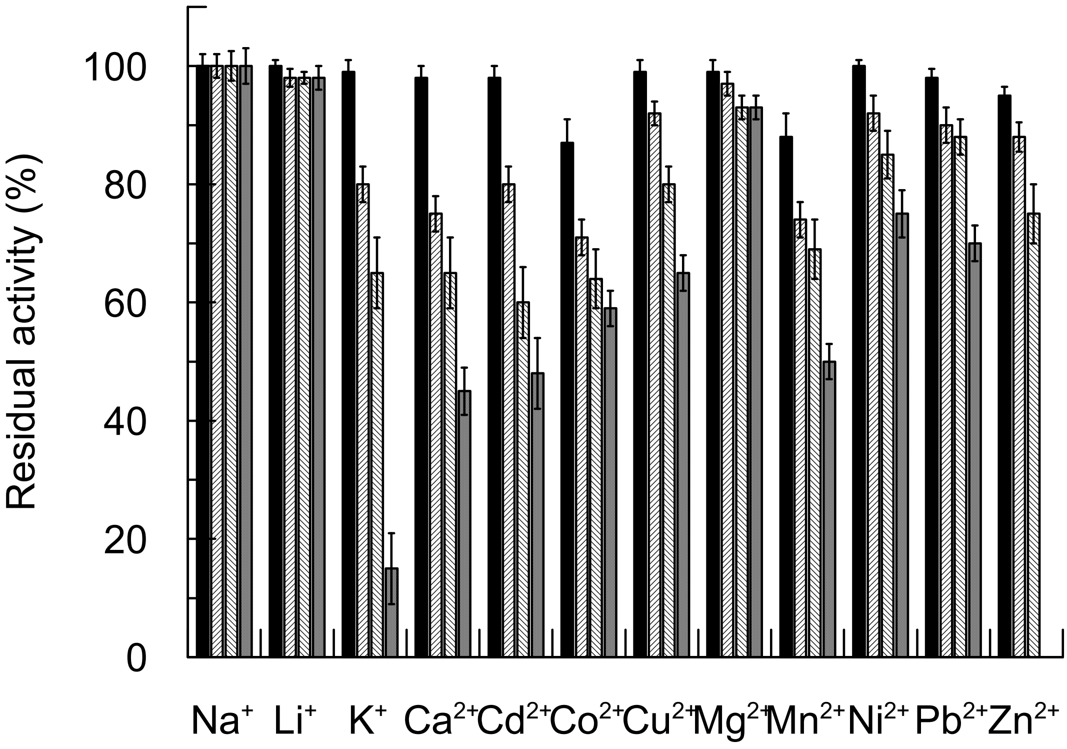

Figure S4. Effect of metal ions on the Bcl1 activity. Four different concentrations of metal ions were tested – in each set from left to right: 1, 5, 10 and 25 mM. N=3.

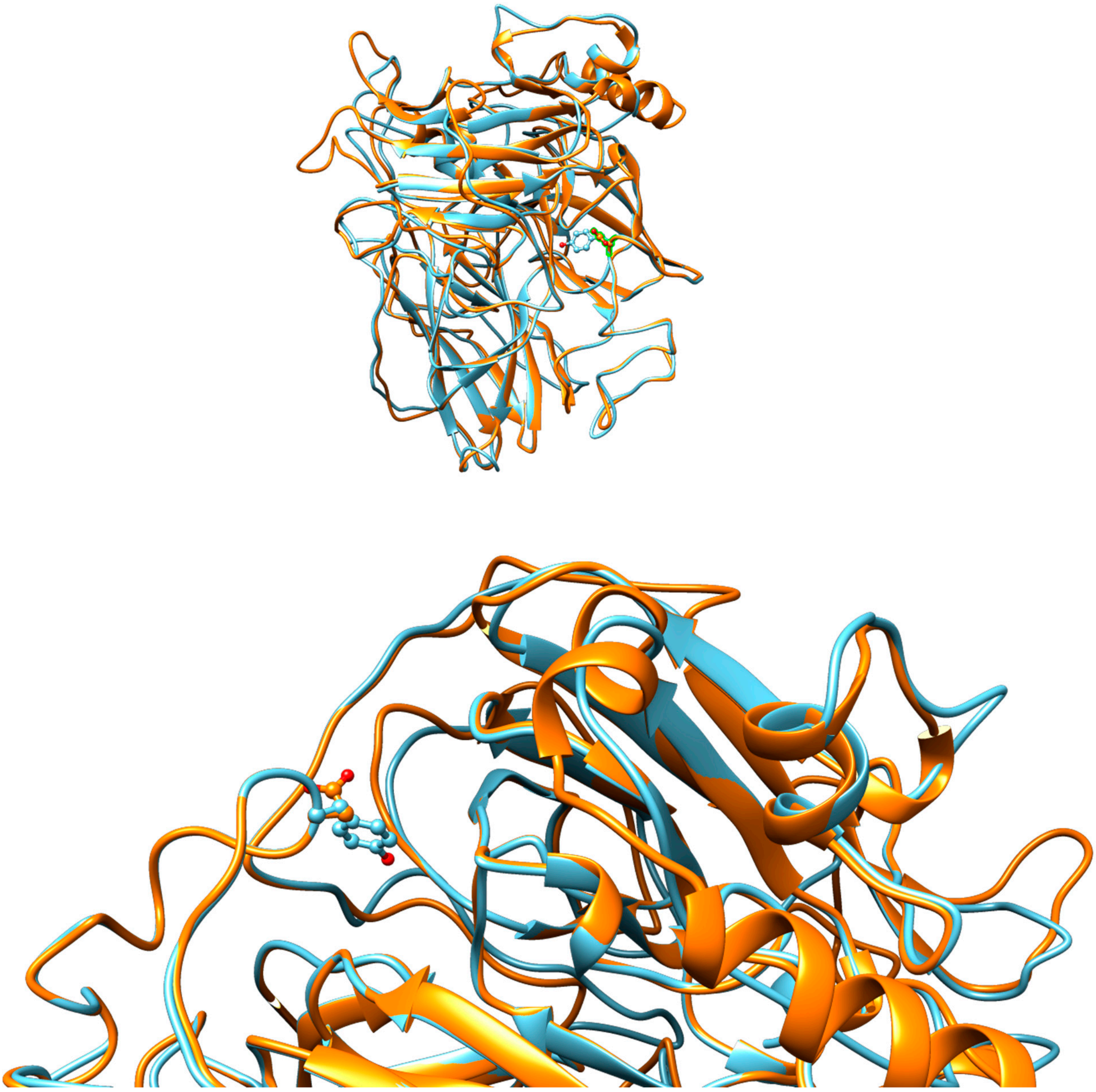

Figure S5. Comparison of 3D models of the laccases from *Botrytis cinerea* 241 (Bcl1, orange) and *Sclerotinia sclerotiorum* (blue). Y65, which is involved in binding of phenolic compounds, in a flexible loop of the laccase from *Sclerotinia sclerotiorum* and D132 in Bcl1 are shown.

Table 1. Purification of laccases Bcl1 and Bcl2 from a 3 L of culture.

Purification step	Protein, mg	Total activity, U	Specific activity, U/mg	Yield, %	Purification (fold)
			Bcl1		
Culture liquid	6800	506	0.074	100	1
CM Toyopearl 650 M chromatography	29	292	10	57.7	135
Source PHE 15 chromatography	2.17	165	76	32.6	1027
			Bcl2		
Culture liquid	3900	12621	3.2	100	1
Clarification with DEAE	3400	12600	3.7	100	1.1
Ultrafiltration	785	10200	12.9	81	3.9
PHE FF Sepharose chromatography	130	7658	59	60.7	18.2
Super Q Toyopearl 650M chromatography	33	4208	127	33	39.2
Source PHE 15 chromatography	26.3	3876	147	30.7	45.4

Table 2. The list of amino acid sequences used for the phylogenetic analysis.

> MT707622 Botrytis cinerea 241
MQASAVDGPLFNILLLSSAAIGAILPAQINERDVLVKRQTVSSSKTSTSSKASSTSSKASSSTSKSTSSST
VKSSSVGSSSSKASTITSGTSTSAGTTTTLSSSSTSALSSLVADPACTNSPFTRACWGNGFSIATDFDTKN
PNTGVTRKYNWEVTNTTCAPDGITRQDCMLVNGQYPGPTLYADWGDMIQVTLKNSMPDNGTGIHWHGLRQY
HTCTEDGVPGNTECPLAPGDTKTYTFQATQFGTSWYHSHYSSQYGEGMLGGIVINGPATSNYDVDLGVYTI
SDWYYTPVFALGERIAHSQAGPPSGDNGLINGSMVAPAGQTGGKYTTNTITAGKKYRLRLINTSVDNHFMV
SWDNHAFTVITSDFVPIVPYTANWIFIGIGQRYDVIITANQTVGSYWFRAEVQNGCGTNNNNGNIKSIFTY
SGAASTIPSSSATPYTGRCTDETGIIPFWDSFVPSGPLSGNVEQLNVAINIGVDASGPIVTWGINLSAIDV
DWKKPILQYVLDGNNSWPASENLIELPNAAQWYYWVIQEVPGNVNGNPVSINVPHPMHLHGHDFFLLGTGV
GTYNNTINGPNLDYDNPTRRDVAMLPAGGWMVLAFQTDNPGAWLMHCHIAWHVSEGLAVQFLETKDQINAV
NPISPSLTNTCNKWNAWYPSQAPYIKTDSGV
>AFV15790.1 laccase [Leucoagaricus gongylophorus]
MLLVTPRFAFLLTLAAVVVATTLGPMTTLTIANKDISPDGFSRSATVVNGIHPGPVIAVTKGDRLRVNVV
NQLTDSVQERGTSVHWHGILQKGTSPMDGTAGVTQCPIAPNSSFQYSFSADVAGTYWYHSHLGNQYCDGL
RGALIVYDKNDPHKDLYDKTTIITLTEWYHIPAPTIQGIAFADSTLINGLGRYPGGPKTNLAVVNVQKGK
RYRFRIFAMSCEPNFTFSVDGHNLTVIEADSKETECKTVNRVQLFAGQRYSAVLNANQPVDNYWIRALPN
TGGNGLNTTFAGGLNSAILRYKGAPVADPTTSQQTQNDKLLETDLHPLNHPVAPGRSTPDGADYVFNVTM
GFDEEAFTWTMNGVQFTAPDVPVLLQILSGAHTAQELLPKGSFLVVERNKTVQINFPSGLIGGPHPFHLH
GHSFNVVRSADSGRFNFMNPVQRDTVNVGNTEGDFVSIRFRTDNPGPWILHCHIDFHLVNGLALVIAEAT
EDVSPLNPPLSDNYNQLCPTWNAFSAEEKNLSN
>ABK59827.1 laccase [Ganoderma fornicatum]
MARLQSLLPYFSLLLAASAYAAIGPVANLTISDAEIAPDGFTRAAVVVNGVSPGPLIKGNKGDRFQINVV
NQLTNHTMSKTTSIHWHGLFQEGTNWADGPAFVTQCPIASGNSFLYDFRVPDQAGTFWYHSHLSTQYCDG
LRGPLVVYDPHDPLAHMYDVDDDSTVITLTEWYHTAAQLGRRFPVGDANSTLINGLGRSTATPTADLAVV
NVTQGKRYRFRLVSMACDPSFNFSIDGHDLTVIEADGVETQPVTVSTITIFAAQRYSFVLTANQTIDNYW
IRANPAFGDVGFAGGLNSAILRYDGAAPIEPVTSQQSTQILLNETDLHPYVPKKTPGKPKKGGVDLALNM
VFGFNTTNFFINNATFVPPTVPVLLQILSGAQSAQDLLPAGSVYTLPKNASIEITFPANANAAGSPHPFH
LHGHTFAVVRSAGSTAYNYDNPVWRDTVSTGLASANDNVTIRFQTDNPGPWFLHCHIDFHLNAGFAVVLA
EDVPDVAYANPVPQEWKNLCPTYDALSPNDQ
>AFE48785.1 yellow laccase [Stropharia aeruginosa]
MQVQALLRFLEFSFVVVGAYAAIGPSTNLFIENKFIQPDGFNRSTVLAGETADSVSFPGPLIVGNTGDVF
TINVIDELVDQTMLTSTSIHWHGLFQKGSDWADGPVGVTQCPIKPGNSFQYQFSVPGQAGTFWYHSHDST
QYCDGLRGALVVYDPNDPYKPQYDFDDDSTVITLADWYHAPATLLGMTPRAPTPLATLINGKGRYAEDPT
AELAVISVIPNKRYRFRLVSISCDPNFEFTIDNHSMMIIEVDGQNIQPLTVDSITISAGQRYSFILQANK
QVSNYWIRSLPNSGPPGFTNGVNSAILRYVGAPIADPTTVKTVSDPLQEWRLQPLINPAAPGVPILGAAD
KNIYLNITFNGTLFFVNNASFVPPTKPVLLQILSGAHSVHELLPAGSIYELPPNSVIELSMPGGSAGSPH
PIHLHGHTFSVIRSAGSKIYNYNNPVRRDVVSIGDSNDNVTIRFETDNAGPWLLHCHIDWHLDRGLDVVF
AEDIPDIAKQNPPIAWENLCRD
>TFK34060.1 yellow laccase [Crucibulum laeve]
MFISSSLAVLSFLSVTHAAIGPSASLHIENKNISPDGFSRSAVLAGASAGGGVFPGPLITANKGDTFHLN
VIDSLTDTTMLRATSIHWHGFFQKGSSWADGPAGVTQCPISPNHSFLYEFDSAGQAGTFWYHSHHSTQYC

DGLRGAIVVYDRVNDPHRFRYLIDDDSTVITLADWYHTPAPSAGLVPTADATLINGKGRYPGGPTVPLAV
IRVLPLVRYRFRLVSISCDPNYVFSIDGHKLKIIEVDGVNVQPYDVDSIQIFAGQRYSFVLNANRPIGNY
WIRAQPNIGPQGFVGGVNSAILRYWGAPAQDPTTTSTGTVAMLETGLHPLANPGAPGSPTQGGADVAINL
AIAFSFTDLKFTINGATFQQPTIPVLLQILSGAQTAQALLPPGSVYTLPPNKVIELTIPGGTIGSPHPFH
LHGHTFDVVRSAGSSVYNYANPVRRDVVSTGLAGDNVTIRFTTDNPGPWILHCHIDWHLDIGLAVVFAED
TASISTEDPPTSWDNLCPIYDAQPPEEF
>TEB23091.1 yellow laccase [Coprinellus micaceus]
MLKFKLAFASVAQLAAVALAAILPIGNLHIANKVIEPDGFSRSAVLAGSRASTLTFPGPLIVNVKDGPFF
LNVVDALTDATMLRSTSIHWHGFFQAGSAWADGPAGVTQCPIAPGHSFLYKFRAKGQAGTFWYHSHHLSQ
YCDGLRGAIVVYDLHDPHRNRYDVDNPDTVITLADWYHVPAPSAGIVPTPDSTLINGKGRYAGGPVVPLT
KIVVKRNKRYRFRLVSISCDPNYVFSIDSHTMTIIEVDGINVQPLTVDSIQIFAGQRYSFVLTTNQPVAN
YWIRANPNLGTRGFAGGLNSAILRYSGAPDADPTSTQTPNSNPLVEANLHPLSNAAAPGTPTLGAADVTL
NLNIVFDFQALKFRVNGAIFNEPSVPVLLQILSGASTAQDLLPAGSVYVLPRNKVIEVSMPGGSTGSPHP
IHLHGHAFSVVRSAGSSTYNFANPVRRDVVSIGNTGDNVTIRFKTDNPGPWIMHCHIDWHLVLGLSVVFA
EDVPSIASVNLPPPAWDQLCPIYDALPAGTR
>A7EM18 [Sclerotinia sclerotiorum]
MPLDKLVTALGDIASLATLLFGTFVSAQNNYSALPVKYLLTSNPVTPLPQGFPWADKTAN
NTNPYTNPPNTGVIRSYDFTVQRGKASPDGYERDVILVNGVFPGPLIEANWGDTIQVTVH
NEITGPEEGTALHWHGLLQKQSQWFDGVPGVQQCPIPPGGSFTYTFLADLYGTSWWHSHY
SAQYNAGILGPMIIHGPATVPYDIGPILLTDWYHPDYFSLVEDVTSGDLDRVLVLSDSNL
IQGKGFFNCSLKAAGDSATCNDKNFVAPTFKLTPGKRHRLRLINAGTEGTQKFAIDGHTM
TVIANDFVPILPYSTEVVTLGVGQRADVIVQALPLEKSGSYWMRSTLAGCSLASNPAAKA
VAYYGSTPNLNPITIASGSFNNSVANVCANDPLTSTTPWFPITPDPNPPTTQHIDIAFGP
NSTGNYQWTMNNSSFRANYNHPVLLLSNQGNNSYPDSPEWNVYNFGSNSSFRIYITNSSP
VVHPMHLHGHNMFVLNEGVGTWDGHTVVRPINPQRRDVQMLQASGYMVLQVTADNPGVWP
LHCHIAFHVSSGLYVNVVERPADIANLKIPSIMAQTY
>CCD50266.1 hypothetical protein BofuT4_P092250.1 [Botrytis cinerea T4]
MKLFNILLLSSAAIGAILPAQINERDVLVKRQTVSSSKTSTSSKASSTSSKASSSTSKSTSSSTVKSSSVGSSSSKASTI
TSGTSTSAGTTTTLSSSSTSALSSLVADPACTNSPFTRACWGNGFSIATDFDTKNPNTGVTRKYNWEVTNTTCAPDGVTR
QDCMLVNGQYPGPTLYADWGDMIQVTLKNSMPDNGTGIHWHGLRQYHTCTEDGVPGITECPLAPGDTKTYTFQATQFGTS
WYHSHYSSQYGEGMLGGIVINGPATSNYDVDLGVYTISDWYYTPVFALGERIAHSQAGPPSGDNGLINGSMVAPAGQTGG
KYTTNTITAGKKYRLRLINTSVDNHFMVSLDNHAFTVITSDFVPIVPYTANWIFIGIGQRYDVIITANQTVGSYWFRAEV
QNGCGTNNNNGNIKSIFTYSGAASTTPSSSATPYTGRCTDETGIIPFWDSFVPSGPLSGNVEQLNVAINIGVDASGPIVT
WGINLSAIDVDWKKPILQYVLDGNNSWPASENLIELPNAAQWYYWVIQEVPGNVNGNPVSINVPHPMHLHGHDFFLLGTG
VGTYNNTINGPNLDYDNPTRRDVAMLPAGGWMVLAFQTDNPGAWLMHCHIAWHVSEGLAVQFLETKDQINAVNPISPSLT
NTCNKWNAWYPSQAPYIKTDSGL
>EMR81386.1 putative laccase protein [Botrytis cinerea BcDW1]
MKLFNILLLSSAAIGAILPAQINERDVLVKRQTVSSSKTSTSSKASSTSSKASSSTSKSSSSSTVKSSSVGSSSSKASTI
TSGTSTSAGTTTTLSSSSTSALSSLVADPACTNSPFTRACWGNGFSIATDFDTKNPNTGVTRKYNWEVTNTTCAPDGVTR
QDCMLVNGQYPGPTLYADWGDMIQVTLKNSMPDNGTGIHWHGLRQYHTCTEDGVPGITECPLAPGDTKTYTFQATQFGTS
WYHSHYSSQYGEGMLGGIVINGPATSNYDVDLGVYTISDWYYTPVFALGERIAHSQAGPPSGDNGLINGSMVAPAGQTGG
KYTTNTITAGKKYRLRLINTSVDNHFMVSLDNHAFTVITSDFVPIVPYTANWIFIGIGQRYDVIITANQTVGSYWFRAEV
QNGCGTNNNNGNIKSIFTYSGAASTTPSSSATPYTGRCTDETGIIPFWDSFVPSGPLSGNVEQLNVAINIGVDASGPIVT
WGINLSAIDVDWKKPILQYVLDGNNSWPASENLIELPNAAQWYYWVIQEVPGNVNGNPVSINVPHPMHLHGHDFFLLGTG
VGTYNNTINGPNLDYDNPTRRDVAMLPAGGWMVLAFQTDNPGAWLMHCHIAWHVSEGLAVQFLETKDQINAVNPISPSLT
NTCNKWNAWYPSQAPYIKTDSGL
>XP_001551072.1 Bclcc7 [Botrytis cinerea B05.10]
MKLFNILLLSSAAIGAILPAQINERDVLVKRQTVSSSKTSTSSKASSTSSKASSSTSKSSSSSTVKSSSVGSSSSKASTI
TSGTSTSAGTTTTLSSSSTSALSSLVADPACTNSPFTRACWGNGFSIATDFDTKNPNTGVTRKYNWEVTNTTCAPDGVTR
QDCMLVNGQYPGPTLYADWGDMIQVTLKNSMPDNGTGIHWHGLRQYHTCTEDGVPGITECPLAPGDTKTYTFQATQFGTS
WYHSHYSSQYGEGMLGGIVINGPATSNYDVDLGVYTISDWYYTPVFALGERIAHSQAGPPSGDNGLINGSMVAPAGQTGG
KYTTNTITAGKKYRLRLINTSVDNHFMVSLDNHAFTVITSDFVPIVPYTANWIFIGIGQRYDVIITANQTVGSYWFRAEV
QNGCGTNNNNGNIKSIFTYSGAASTTPSSSATPYTGRCTDETGIIPFWDSFVPSGPLSGNVEQLNVAINIGVDASGPIVT
WGINLSAIDVDWKKPILQYVLDGNNSWPASENLIELPNAAQWYYWVIQEVPGNVNGNPVSINVPHPMHLHGHDFFLLGTG
VGTYNNTINGPSLDYDNPTRRDVAMLPAGGWMVLAFQTDNPGAWLMHCHIAWHVSEGLAVQFLETKDQINAVNPISPSLT
NTCNKWNAWYPSQAPYIKTDSGL
>THV49145.1 hypothetical protein BGAL_0209g00140 [Botrytis galanthina]
MKLLNILLLSSAAIGAILPAQINERDGLVRRQTVSSSKTSTSSKASSTSSKTSSGTSKSSSSSTLKSSSVGSSSSKVSTT
TSGTSTSVGTTTTLSSSSTSALSSLVADPACTNSPFTRACWGNGFSIATDFDTKNPNTGVTRKYNWEVTNTTCAPDGVTR
QNCMLVNGQYPGPTLYADWGDMIQVTLKNSMPDNGTGIHWHGLRQYHTCTEDGVPGITECPLAPGDTKTYTFQATQFGTS
WYHSHYSAQYGEGMLGGIVINGPATSNYDVDLGVYTISDWYYTPVFALGERIAHSQAGPPSGDNGLINGSMVAPAGQTGG

KYTTNTITAGKKYRLRLINTSVDNHFMVSLDNHAFTVITSDFVPIVPYTTNWIFIGIGQRYDVIITANQTVGSYWFRAEV
QNGCGTNNNNGNIKSIFTYSGAASTTPSSSATPYTGRCTDETGLIPFWDSFVPSGPLSGNVDQLNVAINIGVDASGPIVT
WGINLSAIDVDWKKPILQYVLDGNNSWPASENLIELPNAAQWYYWIIQEVPGNVNGNPVSINVPHPMHLHGHDFFLLGTG
VGTYNNTINAPNLDYDNPTRRDVAMLPAGGWMVLAFQTDNPGAWLMHCHIAWHVSEGLAVQFLETKNQINAVNPISPALT
NTCNKWNAWYPSQAPYLKTDSGL
>TGO38043.1 hypothetical protein BHYA_0083g00220 [Botrytis hyacinthi]
MKLLNILLLSSAAIGAILPAQINERDGLVKRQTVSSSKTSTSSKASSTSSKTSSGTSKSSSSSTLKSSSVGSSSSKASTT
TSGTSTSVGTTTTLSSSSTSALSSLVADPACTNSPFTRACWGNGFSIATDFDTKNPNTGVTRKYNWEVTNTTCAPDGVTR
QDCMLVNGQYPGPTLYADWGDMIQVTLKNSMPDNGTGIHWHGLRQYHTCTEDGVPGITECPLAPGDTKTYTFQATQFGTS
WYHSHYSSQYGEGMLGGIVINGPATSNYDVDLGVYTISDWYYTPVFALGERIAHSQAGPPSGDNGLINGSMVAPAGRTGG
KYTTNTITAGKKYRLRLINTSVDNHFMVSLDNHAFTVITSDFVPIVPYTTNWIFIGIGQRYDVIITANQTVGSYWFRAEV
QNGCGTNNNNGNIKSIFTYSGAASTTPSSSATPYTGRCTDETGIIPFWDSFVPSGPLSGNVDQLDVAINIGVDASGPIVT
WGINLSAIDVDWKKPILQYVLDGNNSWPASANLIELPNAAQWYYWIIQEVPGNVNGNPVSINVPHPMHLHGHDFFLLGTG
VGTYNNTINAPNLDYDNPTRRDVAMLPAGGWMVLAFQTDNPGAWLMHCHIAWHVSEGLAVQFLETKNLINAVNPISPALT
NTCNKWNAWYPSQAPYLKTDSGL
>KAF7857842.1 hypothetical protein EAF02_011209 [Botrytis sinoallii]
MKLLNILLLSSAAIGAILPAQLNERDGLVKRQTVSSSKTSTSSKASGTSSKTSSGTSKSSSSSTLKSSSVGSSSSKASTT
TSGTSTTVGTTTTLSSSSSSALSSLVADPACTNSPFTRACWGNGFSIATDFDTKNPNTGVTRKYNWEVTNTTCAPDGVTR
QNCMLVNGQYPGPTLYADWGDMIQVTLKNSMPDNGTGIHWHGLRQYHTCTEDGVPGITECPLAPGDTKTYTFQATQFGTS
WYHSHYSSQYGEGMLGGIVINGPATSNYDVDLGVYTITDWYYTPVFALGERIAHSQAGPPSGDNGLINGSMVAPAGQSGG
KYTTNTITAGKKYRLRLINTSVDNHFMVSLDNHAFTVITSDFVPIVPYTTNWIFIGIGQRYDVIITANQTVGSYWFRAEV
QNGCGTNNNNGNIKSIFTYSGAASTTPSSSATPYTGRCTDETGIIPFWDSFVPSGPLSGNVDQLNVAVNIGVDASGPIVT
WGINLSAIDVDWKKPILQYVLDGNNSWPASENLIELPNAAQWYYWIIQEVPGNVNGNPVSINVPHPMHLHGHDFFLLGTG
IGTYNNTLDSPNLDYDNPTRRDVAMLPAGGWMVLAFQTDNPGAWLMHCHIAWHVSEGLAVQFLETKNQINAVNPISPALT
NTCNKWNAWYPSQAPYVKTDSGL
>TGO14699.1 hypothetical protein BTUL_0049g00080 [Botrytis tulipae]
MKLLNILLLSSAAIGAILPAQINERDGLVKRQTVSSSKTSTSSKASSTSSKTSSGTSKSSSSSTLKSSSAGSSSSKVSTT
TSGTSTSVGTTTTLSSSSTSALSSLVADPACTNSPFTRACWGNGFSIATDFDTKNPNTGITRKYNWEVTNTTCAPDGVTR
QNCMLVNGQYPGPTLYADWGDMIQVTLKNSMPDNGTGIHWHGLRQYHTCTEDGVPGITECPLAPGDTKTYTFQATQFGTS
WYHSHYSSQYGEGLLGGIVINGPATSNYDVDLGVYTISDWYYTPVFALGERIAHSQAGPPSGDNGLINGSMVAPAGRTGG
KYTTNTINAGKKYRLRLINTSVDNHFMVSLDNHAFTVITSDFVPIVPYTTNWIFIGIGQRYDVIITANQTVGSYWFRAEV
QNGCGTNNNNGNIKSIFTYSGAASTTPSSSATPYTGRCTDETGLIPFWDSFVPSGPLSGNVDQLNVAINIGVDASGPIVT
WGINLSAIDVDWKKPILQYVLDGNNSWPASENLIELPNAAQWYYWVIQEVPGNVNGNPVPINVPHPMHLHGHDFFLLGTG
VGTYNNTINAPNLDYDNPTRRDVAMLPAGGWMVLAFQTDNPGAWLMHCHIAWHVSEGLAVQFLETKNQINAVNPISPALT
NTCNKWNAWYPSQAPYLKSDSGL
>KAF7929827.1 hypothetical protein EAE97_009424 [Botrytis byssoidea]
MKLLNILLLSSAAIGAILPAQINERDGLVKRQTVSSSKTSTSSKASSTSSKASSTSSKTSSGTSKSSSSSTLKSSSAGSS
SSKVSTTTSGTSTSVGTTTTLSSSSTSALSSLVADPACTNSPFTRACWGNGFSIATDFDTKNPNTGVTRKYNWEVTNTTC
APDGVTRQNCMLVNGQYPGPTLYADWGDMIQVTLKNSMPDNGTGIHWHGLRQYHTCTEDGVPGITECPLAPGDTKTYTFQ
ATQFGTSWYHSHYSAQYGEGMLGGIVINGPATSNYDVDLGVYTISDWYYTPVFVLGERIAHSQAGPPSGDNGLINGSMVA
PAGRTGGKYTTNTITAGKKYRLRLINTSVDNHFMVSLDNHAFTVITSDFVPIVPYTTNWIFIGIGQRYDVIITANQTVGS
YWFRAEVQNGCGTNNNNGNIKSIFTYSGAASTTPSSSATPYTGRCTDETGLIPFWDSFVPSGPLSGNVDQLNVAINIGVD
ASGPIVTWGINLSAIDVDWKKPILQYVLDGNNSWPASENLIELPNAAQWYYWIIQEVPGNVNGNPVSINVPHPMHLHGHD
FFLLGTGVGTYNNTINAPNLDYDNPTRRDVAMLPAGGWMVLAFQTDNPGAWLMHCHIAWHVSEGLAVQFLETKNQINAVN
PISPALTNTCNKWNAWYPSQAPYLKTDSGL
>TGO20676.1 hypothetical protein BPAE_0275g00070 [Botrytis paeoniae]
MKFLNILLLSSAAIGAILPAQINERDGLVKRQTVSSSKTSTSSKTSSGASKSSSSSTLKSSSVGSSTSKVSTTTSGTSTS
VGTTTTLSSSSTSALSSLVADPACTNSPFTRACWGNGFSIATDFDTKNPNTGVTRKYNWEVTNTTCAPDGVTRQNCMLVN
GQYPGPTLYADWGDMIQVTLKNSMPDNGTGIHWHGLRQYHTCTEDGVPGITECPLAPGDTKTYTFQATQFGTSWYHSHYS
AQYGEGMLGGIVINGPATSNYDVDLGVYTISDWYYTPVFALGERIAHSQAGPPSGDNGLINGSMVAPAGLTGGKYTTNTI
SAGKKYRLRLINTSVDNHFMVSLDNHAFTVITSDFVPIVPYTTNWIFIGIGQRYDVIITANQTVGSYWFRAEVQNGCGTN
NNNGNIKSIFTYSGAASTTPSSSATPYTGRCTDETGIIPFWDSFVPSGPLSGNVDQLNVAINIGVDASGPIVTWGINLSA
IDVDWKKPILQYVLDGNNSWPASENLIELPNAAQWYYWIIQEVPGNVNGNPVSINVPHPMHLHGHDFFLLGTGVGTYNNT
INAPNLDYDNPTRRDVAMLPAGGWMVLAFQTDNPGAWLMHCHIAWHVSEGLAVQFLETKNQINAVNPISPALTNTCNKWN
AWYPSQAPYLKTDSGL
>KAF7943382.1 hypothetical protein EAE96_011309 [Botrytis aclada]
MKFLNILLLSSATIGAILPAQINERDGLVRRQTVSSSKTSTSSKVSSTSSKTSSGISKSSSSSTLKSSSSVGSSSSNAST
TTSGTSTSVGTTTTLSSSSTSALSSLVADPACTNSPFTRACWGNGFSIATDFDTKNPNTGVTRKYNWEVTNTTCAPDGVT
RQNCMLVNGQYPGPTLYANWGDMIQVTLKNSMPDNGTGIHWHGLRQYHTCTEDGVPGITECPLAPGDTKTYTFQATQFGT
SWYHSHYSAQYGEGMVGGIVINGPATSNYDVDLGVYTISDWYYTPVFALGERIAHSQAGPPSGDNGLINGSMVAPAGQTG
GKYTTNTITAGKKYRLRLINTSVDNHFMVSLDNHAFTVITSDFVPIVPYTTNWIFIGIGQRYDVIITANQTVGSYWFRAE

VQNGCGTNNNNGNIKSIFTYSGAASTTPSSSATPYTGRCTDETGLIPFWDSFVPSGPLSGNVDQLNVAINIGVDASGPIV
TWGINLSAIDVDWKKPILQYVLDGNNSWPASENLIELPNAAQWYYWIIQEVPGNVNGNPVSINVPHPMHLHGHDFFLLGT
GVGTYNNTINAPNLDYDNPTRRDVAMLPAGGWMVLAFQTDNPGAWLMHCHIAWHVSEGLAVQFLETKNQINAVNPISPAL
TNTCNKWNAWYPSQAPYLKTDSGL
>KAF7933257.1 hypothetical protein EAE99_003142 [Botrytis elliptica]
MKLLNILLLSSAAIGAILPAQINERDGLVKRQTVSSSKTSTSSKASGTSSKASSTSSKTSSGTSKSSSSSTLKPSSVGSS
SSKASTTTSGTSTSVGTTTTLSSSSTSALSSLVADPACTNSPFTRACWGNGFSIATDFDTKNPNTGVTRKYNWEVTNTTC
APDGVTRQNCMLINGQYPGPTLYADWGDMIQVTLKNSMPDNGTGIHWHGLRQYHTCTEDGVPGITECPLAPGDTKTYTFQ
ATQFGTSWYHSHYSSQYGEGMLGGIVINGPATSNYDVDLGVYTITDWYYTPVFALGERIAHSQAGPPSGDNGLINGSMVA
PAGQSGGKYTTNTITAGKKYRLRLINTSVDNHFMVSLDNHAFTVITSDFVPIVPYTTNWIFIGIGQRYDVIITANQTVGS
YWFRAEVQNGCGTNNNNGNIKSIFTYSGAASTTPSSSATPYTGRCTDETGIIPFWDSFVPSGPLSGNVDQLDVAVNIGVD
ASGPIVTWGINLSAIDVDWKKPILQYVLDGNNSWPASENLIELPNAAQWYYWIIQEVPGNVNGNPVSINVPHPMHLHGHD
FFLLGTGIGTYNNTLDSPNLDYDNPTRRDVAMLPAGGWMVLAFQTDNPGAWLMHCHIAWHVSEGLAVQFLETKNQINAVN
PISPALTNTCNKWNAWYPSQAPYIKTDSGL
>TGO72679.1 hypothetical protein BELL_0429g00110 [Botrytis elliptica]
MKLLNILLLSSATIGAILPAQINERDGLVKRQTVSSSKTSTSSKASGTSSKASSTSSKTSSGTSKSSSSSTLKSSSVGSS
SSKASTTTSGTSTSVGTTTTLSSSSTSALSSLVADPACTNSPFTRACWGNGFSIATDFDTKNPNTGVTRKYNWEVTNTTC
APDGVTRQNCMLINGQYPGPTLYADWGDMIQVTLKNSMPDNGTGIHWHGLRQYHTCTEDGVPGITECPLAPGDTKTYTFQ
ATQFGTSWYHSHYSSQYGEGMLGGIVINGPATSNYDVDLGVYTITDWYYTPVFALGERIAHSQAGPPSGDNGLINGSMVA
PAGQSGGKYTTNTITAGKKYRLRLINTSVDNHFMVSLDNHAFTVITSDFVPIVPYTTNWIFIGIGQRYDVIITANQTVGS
YWFRAEVQNGCGTNNNNGNIKSIFTYSGAASTTPSSSATPYTGRCTDETGIIPFWDSFVPSGPLSGNVDQLDVAVNIGVD
ASGPIVTWGINLSAIDVDWKKPILQYVLDGNNSWPASENLIELPNAAQWYYWIIQEVPGNVNGNPVSINVPHPMHLHGHD
FFLLGTGIGTYNNTLDSPNLDYDNPTRRDVAMLPAGGWMVLAFQTDNPGAWLMHCHIAWHVSEGLAVQFLETKNQINAVN
PISPALTNTCNKWNAWYPSQAPYIKTDSGL
>KAF7911640.1 hypothetical protein EAE98_011905 [Botrytis deweyae]
MKLLNILLLSSAAIGAILPAQINERDGLVKRQTVSSSKTSTSSKASGTSSKASSTSSKTSSGTSKSSSSSTLKSSSVGSS
SSKASTTTSGTSTSVGTTTTLSSSSTSALSSLVADPACTNSPFTRACWGNGFSIATDFDTKNPNTGVTRKYNWEVTNTTC
APDGVTRQNCMLINGQYPGPTLYADWGDMIQVTLKNSMPDNGTGIHWHGLRQYHTCTEDGVPGITECPLAPGDTKTYTFQ
ATQFGTSWYHSHYSSQYGEGMLGGIVINGPATSNYDVDLGVYTITDWYYTPVFALGERIAHSQAGPPPGDNGLINGSMVA
PAGQSGGKYTTNTITAGKKYRLRLINTSVDNHFMVSLDNHAFTVITSDFVPIVPYTTNWIFIGIGQRYDVIITANQTVGS
YWFRAEVQNGCGTNNNNGNIKSIFTYSGAASTTPSSSATPYTGRCTDETGIIPFWDSFVPSGPLSGNVDQLDVAVNIGVD
ASGPIVTWGINLSAIDVDWKKPILQYVLDGNNSWPASENLIELPNAAQWYYWIIQEVPGNVNGNPVSINVPHPMHLHGHD
FFLLGTGIGTYNNTLDSPNLDYDNPTRRDVAMLPAGGWMVLAFQTDNPGAWLMHCHIAWHVSEGLAVQFLETKNQINAVN
PISPALTNTCNKWNAWYPSQAPYIKTDSGL
>XP_037195563.1 putative laccase protein [Botrytis fragariae]
MKLLNILLLSSAAIGAILPAQINERDGLVNRQTVSSSKTSTSSKVSSTSSKASSTSSKTSSGTSKSSSSSTLKSSSVGSS
SSKVSTTTSGTSTSVGTTTTLSSSSTSALSSLVADPACTNSPFTRACWGNGFSIATDFDTKNPNTGVTRKYNWEVTNTTC
APDGVTRQNCMLVNGQYPGPTLYADWGDMIQVTLKNSMPDNGTGIHWHGLRQYHTCTEDGVPGITECPLAPGDTKTYTFQ
ATQFGTSWYHSHYSAQYGEGMLGGIVINGPATSNYDVDLGVYTISDWYYTPVFALGERIAHSQAGPPSGDNGLINGSMVA
PAGQTGGKYTTNTITAGKKYRLRLINTSVDNHFMVSLDNHAFTVITSDFVPIVPYTTNWIFIGIGQRYDVIITANQTVGS
YWFRAEVQNGCGTNNNNGNIKSIFTYSGAGSTTPSSSATPYTGRCTDETGIIPFWDSFVPSDPLSGNVDQLNVAINIGVD
ASGPIVTWGINLSAIDVDWKKPILQYVLDGNNSWPASENLIELPNAAQWYYWIIQEVPGNVNGNPVSINVPHPMHLHGHD
FFLLGTGVGTYNNTINSPNLDYDNPTRRDVAMLPAGGWMVLAFQTDNPGAWLMHCHIAWHVSEGLAVQFLETKNQINAVN
PISPALTNTCNKWNAWYPSQAPYLKTDSGL
>KAF7912300.1 hypothetical protein EAF01_001321 [Botrytis porri]
MKLLNILLLSSAAIGAILPSQLDERDGLVRRQTVSSSKTSTSSKASNTSSKASSTSSKTSSGTSKSSSSSTLQSSSVGSS
SSKSSTTTSGTSTSVGTTTTLSSSSTSALSSLVADPACTNSPFTRACWGNGFSIATDFDTKNPNTGVTRKYNWEVTNTTC
APDGVTRQNCMLVNGQYPGPTLYADWGDMIQVTLKNSMPDNGTGIHWHGLRQYHTCTEDGVPGITECPLAPGDTKTYTFQ
ATQFGTSWYHSHYSAQYGEGMLGGIVINGPATSNYDVDLGVYTISDWYYTPVFALGERIAHSQAGPPSGDNGLINGSMVA
PAGATGGKYTTNTITSGKKYRLRLINTSVDNHFMVSLDNHAFTVITSDFVPIVPYTTNWIFIGIGQRYDVIITANQTVGS
YWFRAEVQNGCGTNNNNGNIKSIFTYSGAASATPSSSATPYTGRCTDETGLIPFWDSFVPSGPLSGNVDQLNVAIDIGVD
ASGPIVTWGINLSAIDVDWKKPILQYVLDGNNSWPASENLIELPNAAQWYYWIIQEVPGNVNGNPVSINVPHPMHLHGHD
FFLLGTGVGTYNNTINAPNLDYDNPTRRDVAMLPAGGWMVLAFQTDNPGAWLMHCHIAWHVSEGLAVQFLETKNQINAVN
PISPALTNTCNKWNAWYPSQAPYLKTDSGL
>TGO84187.1 hypothetical protein BPOR_0537g00020 [Botrytis porri]
MKLLNILLLSSAAIGAILPSQLDERDGLVRRQTVSSSKTSTSSKASNTSSKASSTSSKTSSGTSKSSSSSTLQSSSVGSS
SSKSSTTTSGTSTSVGTTTTLSSSSTSALSSLVADPACTNSPFTRACWGNGFSIATDFDTKNPNTGVTRKYNWEVTNTTC
APDGVTRQNCMLVNGQYPGPTLYADWGDMIQVTLKNSMPDNGTGIHWHGLRQYHTCTEDGVPGITECPLAPGDTKTYTFQ
ATQFGTSWYHSHYSAQYGEGMLGGIVINGPATSNYDVDLGVYTISDWYYTPVFALGERIAHSQAGPPSGDNGLINGSMVA
PAGATGGKYTTNTITSGKKYRLRLINTSVDNHFMVSLDNHAFTVITSDFVPIVPYTTNWIFIGIGQRYDVIITANQTVGS
YWFRAEVQNGCGTNNNNGNIKSIFTYSGAASATPSSSATPYTGRCTDETGLIPFWDSFVPSGPLSGNVDQLNVAIDIGVD

ASGPIVTWGINLSAIDVDWKKPILQYVLDGNNSWPASENLIELPNAAQWYYWIIQEVPGNVNGNPVSINVPHPMHLHGHD
FFLLGTGVGTYNNTINAPNLDYDNPTRRDVAMLPAGGWMVLAFQTDNPGAWLMHCHIAWHVSEGLAVQFLETKNQINAVN
PISQALTNTCNKWNAWYPSQAPYLKTDSGL
>Q96UM2.1 RecName: Full=Laccase-3; AltName: Full=Benzenediol:oxygen oxidoreductase 3; AltName: Full=Diphenol oxidase 3; AltName: Full=Urishiol oxidase 3 [Botrytis cinerea]
PGPTLYADWGDMIQVTLKNSMPDNGTGIHWHGLRQYHTCTEDGVPGITECPLAPGDTKTYTFQATQFGTSWYHSHYSSQY
GEGMLGGIVINGPATSNYDVDLGVYTISDWYYTPVFALGERIAHSQAGPPSGDNGLINGSMVAPAGQTGGKYTTNTITAG
KKYRLRLINTSVDNHFMVSLDNHAFTVITSDFVPIVPYTANWIFIGIGQRYDVIITANQTVGSYWFRAEVQNGCGTNNNN
GNIKSIFTYSGAASTTPSSSATPYTGRCTDETGIIPFWDSFVPSGPLSGNVEQLNVAINIGVDASGPIVTWGINLSAIDV
DWKKPILQYVLDGNNSWPASENLIELPNAAQWYYWVIQEVPGNVNGNPVSINVPHPMHLHGHDFFLLGTGVGTYNNTING
PSLDYDNPTRRDVAMLPAGGWMVLAFQTDNPGAWLMHCHIAWHVSEGLAVQFQG
>KAF7916227.1 hypothetical protein EAE98_010812 [Botrytis deweyae]
MSFTLLLCIAILIPACLPSALPSKVSSNNKRISTPTCLNAASSRNCWNGNYSIDTNSEEAWPNTGVVVAYTLTITNQTMS
PDGTPRWMLVVNGTYPGPTITANWGDTLEITVVNQIMTDGVSIHWHGIVQKNTNTMDGVNGITECPIPPGSSKVYKFLAT
QHGTSWYHSHHAAQYGDGVLGSIVINGPASLNYDYDLGPLPITDFYYKSTYEEGLVSVSKGPPKADNGLINGTNRNPHKT
AGVYNQVSGLVPGAKYRLRIINMSIDNHFQVTLDSHSFQVIQTDFVPIQPYTTNTIFIAIGQRYDVIITADQTPSNYWFR
AIVPKPPTQKGFGCGQNANNGSINAIFSYQGVELSEPNSKSFLIPQSCEDETQLVSWNEKSVPENEFVWPTAQELIVTGP
GSNSLPKAPAPYVWSINDTYMEIEWDKPTLQYVAQGNKNYNVHQSIIELPDANVWTFWIIKNAAAIPHPIHLHGHDFYIL
GQENGVNFTDKSSLNFANPARRDVAMLPALGYLVIAFVTDNPGAWLLHCHIAFHVGEGLALQFLERELEIAQRVDLSTVD
AGCKRWDTWYNTERVDKAWNTEFDSGI
>THV54741.1 hypothetical protein BGAL_0021g00440 [Botrytis galanthina]
MYFTLLLCIAILIPSCLPSAIPSQALSAIRRTPFSNCLNAASSRNCWNGNYSIDTDSEEAWPNTGVVVAYTLTITNQTMS
PDGTPRWMLVVNGTYPGPTITANWGDTLEITVVNQIITDGVSIHWHGVVQKNTNTMDGVNGITECPIPPGSSKVYKFLAT
QHGTSWYHSHHAAQYGDGVLGSIVINGPASLNYDYDLGPLPITDFYYRSAYEEGLLSVSKGPPKADNGLINGTNRNPHKT
AGVYNRVSGLVPGAKYRLRIINTSIDNHFQVTLDSHFFQVIQTDFVPIQPYTTNTIFIAIGQRYDVIITANQTPSNYWFR
AIVPKPPTQKGFGCGQNANNGSINAIFSYQGIELSKPTSKSFLIPQSCEDETQLVSWHEKAVPETEFVWPTAQELIVTGP
GSNSLPKAPAPYVWSINDTYMEIEWDNPTLQYVAQGNKNYNVHQSIIELPDANVWTFWIIKNAAAIPHPIHLHGHDFYIL
GQENGANFTDKSSLNFANPARRDVAMLPALGYLVIAFVTDNPGAWLLHCHIAFHVGEGLALQFLERESEIAQRVDLSTVD
AGCKRWDTWYNTERVDNAWNTEFDSGI
>KAF7891547.1 hypothetical protein EAF02_001872 [Botrytis sinoallii]
MSFTLLLCIIILIPACLPSALPSKVSSNNKRTSTSTCLNAASSRNCWNGNYSIDTDSEEAWPNTGVVVAYTLTITNQTMS
PDGTPRWMLVVNGTYPGPTITANWGDTLEITVINQIITDGVSIHWHGIVQKNTNTMDGVNGITECPIPPGSSKVYKFLAT
QHGTSWYHSHHAAQYGDGVLGSIVINGPASLNYDYDLGPLPITDFYYKSTYEEGLLSVSKGPPKADNGLINGTNRNPHKT
AGVYNQISGLVPGAKYRLRIINTSIDNHFQVTLDSHSFQVIQTDFVPIQPYTTNNIFIATGQRYDVIITADQTPSNYWFR
AIVPKPPAQKGFGCGQNANNGSINAIFSYQGVELSEPNSKSFLIPQSCEDETQLVSWNEKSVPENEFVWPTAQELIVTGP
GSNSLPKAPAPYVWSINDTYMEIEWDKPTLQYVAQGNKNYNVHRSIIELPDANVWTFWIIKNAAAIPHPIHLHGHDFYIL
GQENGVNFTDKTSLNFANPARRDVAMLPALGYLVIAFVTDNPGAWLLHCHIAFHVGEGLALQFLERESEITQRVDLSTVD
AGCKRWDTWYNTERVDNAWNTEFDSGI
>XP_024552410.1 Bclcc12 [Botrytis cinerea B05.10]
MFFTVMLSIAILIRTCLPSAIPSQALSNNKRTSASTCLNAASSRNCWNGNYSINTDTEEAWPDTGVVVAYTLVITNQTMS
PDGTPRWMLVVNGTYPGPTITASESAPMWPPSSSAMSAYLFIDWGDTLEITVVNQIITDGVSMHWHGIVQKNTNTMDGVN
GITECPIPPGSSKLYKFLATQHGTSWYHSHHAAQYGDGVLGSIVVNGPASLNYDHDLGPLPITDFYYNSTYEEGLLSVSR
GPPKADNGLINGTNRNPQKTAGVYNQVSGLVPGAKYRLRIINTSIDNHFQVTLDSHYFQVIQTDFVPIQPYTTKTIFIAI
GQRYDVIITADQTPSNYWFRAIVPNPPTQKGFGCGQNANNGSINAIFSYQGVESSEPTSKSFLMPQSCEDETQLVSWHEK
AVPEDEFVWPTSQELIVTGPGSNSLSKAPAPYIWSINDTYMKIEWDKPTLQYVIQGNKSYNVHQSIIELPDANVWTFWII
KNAAAIPHPIHLHGHDFYILGQENGINFTDKSSLNFANPARRDVAMLPALGYLVIAFVTDNPGAWLLHCHIAFHVGEGLA
LQFLERESEIAQRVDLSTVDTGCKMWNAWYNTERVDNAWNTEFDSGI
>XP_037188192.1 putative laccase precursor protein [Botrytis fragariae]
MSGALSLLAELDKETNKAPSRKNSQAKPRTSRSTANSRSSSVQSRGPSQTQRPTSAPGPSPVIPPQNITPRRTTQSTNVT
PRTSFELGTERSRSQGPDSRRVSFAEAPRPQTRTLMIPSKENMPSSGFPYNIKLNKYGVSEHEWSQFTKEIIETLPPSKS
FSWLWKRGKITAIIKRDLQYESELKSALRQWNKGFRRRGFQVYLEIPVEKDLTDDEVSGDTKEEKKQAKKDAKKFRFIIG
AGNSSSSSVYSRTSLAESVSREDKAKPAPISREEEDQLNQKFPQAQTNGTGTATSTTEGGHDEDESSIEKAPILSPSTDT
VDHAPGSTQGIGPTERRDLYTEICILDENLTITSGGFMGLLLLWPLIPRIALPTFVMELALSNIKRTSTSNCLNTASSRN
CWNGNYSIDTDSEEAWPDTGVVVAYTLTITNQTMSPDGTPRWMLVVNGTYPGPTITANWGDTLEITVANQIITDGVSIHW
HGIVQKNTNTMDGVNGITECPIPPGSSKIYKFLATQHGTSWYHSHHAAQYGDGVLGSIVINGPASLNYDHDLGPLPITDF
YYKSTYEEGLLSVSKGPPKADNGLINGTNRNPHKTAGVYNQASGLVPGAKYRLRIINTSIDNHFQVTLDSHSFQVIQTDF
VPIQPYTTNTVFIAIGQRYDVIITADQTPSNYWFRAIVPKPPTQKGFGCGQNANNGSINAIFSYQGVELSEPTSNSFLIP
QSCEDETQLVSWHEKAVPENEFVWPTAQELVVTGPGSSSLPKAPAPYIWSINDTYMEIEWDKPTLQYVTQGNKNYNVHQS
IIELPDANVWTFWIIKNAAAVPHPIHLHGHDFYILGQENSVNFTDKSTLNFANPARRDVAMLPALGYLVIAFVTDNPGAW
LLHCHIAFHVGEGLALQFLERESEIAQRVDLSAVDAECKTWDAWYNTERVDNAWNTEFDSGV
>KAF7941588.1 hypothetical protein EAE97_006425 [Botrytis byssoidea]

MYFTLLLCIAILIPSCLPSAIPSQVLSNNKRASASTCLNSASSRNCWNGKYSIDTDSEEAWPNTGVVVAYTLIIKNQTMS
PDGTPRWMLVVNGTYPGPTITASEFFPLWPPLPSAMLAHLFTDWGDTLEITVVNQIITDGVSIHWHGIVQKNTNTMDGVN
GITECPIPPGSSKVYKFLATQHGTSWYHSHHAAQYGDGVLGSIVINGPASLNYDYDLGPLPITDFYYKSTYEEGLLSVSK
GPPKADNGLINGTNRNPHKTAGAYNQVSGLVPGAKYRLRIINTSIDNHFQVTLDSHSFQVIQTDFVPIQPYTTNTIFIAI
GQRYDVIITADQTPSNYWFRAIVPKPPIQKGFGCGQNANNGSIKAIFSYQGVESSEPDSKSFLIPQSCEDETHLVSWHEK
SVPENEFLWPTAQELIVTGPGSNSVPKVPAPYVWSINDTYMEIEWDKPTLQYVAQGNKNYNVHQSIIELPDANVWTFWII
KNAAAIPHPIHLHGHDFYILGQENGVNFTDKSSLNFAKPARRDVAMLPALGYLVIAFVTDNPGAWLLHCHIAFHVGEGLA
LQFLERESEIAQRVNLSTVDAGCKRWDTWYSIERVDNAWNTEFDSGI
>TGO16908.1 hypothetical protein BTUL_0022g00090 [Botrytis tulipae]
MYFTLLLCIAILVPSCLPSAIPSQVLTNNKRTSTSTCLKAASSRNCWNGNYSIDTDSEEAWLNTGVVVAYTLTITNQTMS
PDGTPRWMLVANGTYPGPTITSSEFFPMWPPFPSAMLAHLFTDWGDTLEITVVNQIITDGVSIHWHGIVQKNTNTMDGVN
GITEYPIPPGSSKVYKFLATQHGTSWYHSHHAAQYGDGVLGSIVINGPASLNYDYDLGPLPITDCYYKSTYDEGLLSVSK
GPPKADNGLINGTNRNPHKTAGVYNQVSGLVPGAKYRLRIINTSMDNHFQVTLDSHSFQVIQTDFVPIQPYTTNTIFIAI
GQRYNVIITADQTPSNYWFRAIVPKPPTQKGFGCGQNANNGSINAIFSYQGVESSEPDSKSFLIPQSCEDETQLVSWHEK
SVPENEFAWPTAQELIVTGPGSNSLPKAPAPYVWSINDTYMEIEWDKPTLQYVAQGNNNYNVDQSIIELPDANVWTFWII
KNAAAIPHPIHLYGHDFYILGQENGVNFTDKSSLNFANPARRDVAMLPALRYLVIAFVTDNPGAWLLHCHIAFHVGEGLA
LQFLERESEIAQRVDLSTVDVGCKRWDTWYSTRRVDNAWSTDFDSGI
>TGO29266.1 hypothetical protein BPAE_0017g00740 [Botrytis paeoniae]
MFFTVLLCIAILIPSCLPSAIPSQALSNNKRTSTSNCLNAASSRDCWNGNYSIDTDSEEAWPNTGVVVAYTLTITNQTMS
PDGTPRWMLVVNGTYPGPTITASEFFPMWPPFPSATLAHLFIDWGDTLEITVVNQIITDGVSMHWHGIVQNNTNTMDGVN
GITECPIPPGSSKIYKFLATQHGTSWYHSHHAAQYGDGVLGSIVINGPTSLNYDYDLGPLPITDFYYKSTYEEGLLAISK
GPPIADNGLINGTNRNPHKTAGAYNQVSGLLPGAEYRLRIINTSIDNHFQVTLDSHSFQVIQTDFVPIQPYTTSTIFIAI
GQRYDVIITTDQTPSNYWFRAIIPKPPTQKGFGCGQNANNGSINAIFSYQGVGLLEPTSKSFVIPQNCEDETQLVSWHEK
AVPEDEFVWPTAQELVVTGPGSNSLPKAPTPYIWNINDTYMEIEWDKPMLQYVAQGNKDYNVHQNIIELPDANIWTFWII
KNAAAIPHPIHLHGHDFYILGQENSVNFTDKSTLNFANPARRDVAMLLALGYLVIAFVTDNPGAWLLHCHIAFHVGDGLA
LQFLERESEIAQRGDISIVDAGCQVWDAWYNTERVDNAWNTEFDSGI
>EMR90689.1 putative laccase precursor protein [Botrytis cinerea BcDW1]
MSPDGTPRWMLVVNGTYPGPTITASESAPMWPPSSSAMSAYLFIDWGDTLEITVVNQIITDGVSMHWHGIVQKNTNTMDG
VNGITECPIPPGSSKLYKFLATQHGTSWYHSHHAAQYGDGVLGSIVVNGPASLNYDHDLGPLPITDFYYNSTYEEGLLSV
SRGPPKADNGLINGTNRNPQKTAGVYNQVSGLVPGAKYRLRIINTSIDSHFQVTLDSHYFQVIQTDFVPIQPYTTKTIFI
AIGQRYDVIITADQTPSNYWFRAIVPNPPTQKGFGCGQNANNGSINAIFSYQGVESSEPTSKSFLMPQSCEDETQLVSWH
EKAVPEDEFVWPTSQELIVTGPGSNSLSKAPAPYIWSINDTYMKIEWDKPTLQYVIQGNKSYNVHQSIIELPDANVWTFW
IIKNAAAIPHPIHLHGHDFYILGQENGINFTDKSSLNFANPARRDVAMLPALGYLVIAFVTDNPGAWLLHCHIAFHVGEG
LALQFLERESEIAQRVDLSTVDTGCKMWNAWYNTERVDNAWNTEFDSGI
>TGO27265.1 hypothetical protein BPAE_0045g00640 [Botrytis paeoniae]
MRLPVFLRVLILVPFVFAAQKHGEPSLTKRCVNSADDRSCWGDYDISTNYYNEGPDTGVIREYWWDIVNTTAAPDGIERV
ILSVNGSVPGPTIIADWGDTIVIHVKNSMPDYGTGIHFHGIRQNYTNQMDGVPSITQCPIAPETSFTYTWKATQYGSSWY
HSHFYVQAWDGIFGGIQINGPATANYDEDLGTLTLSDWSHVTAEVEAVAALAAPPTLDNGLINGTNTYNNSGTIVGSRFE
TIFKSGTRYRIRLVNTAADTHFKFSIDNHTMEVIAADFVPIVPYTSDAIAISMGQRYDIIVTANAAVDNYWMRAVAQTVC
SKNVNADNIYGIIRYDSTSINDPTSVRWANASTDELCEDELMSSLIPYVPIVASDSPAEEDEFSVIINKTNGVIWQMGTH
SFLNQWDYPTLLQAYEGNDTWAPEQEVYQLPEANVWVYWIIETTNAQSHPMHLHGHDFWVLGQGTGTYDSTTANLTLVNA
PRRDVVLLPGSGWVVMAFYTDNPGAWIMHCHIAWHTSEGLAVQILERESELVDLLDGDLLNSTCAAWNSYVSADNVIQPD
SGI
>THV56020.1 hypothetical protein BGAL_0001g00820 [Botrytis galanthina]
MKNSFFSSLAKFASLSLALALPTAEIIPSALEERQSCANTATTRSCWGQYSASTNSYTTVPQTGVTREYWLVVQNTTLSA
DGVSRPTLNFNGTIPGPQITADWGDDVIVHVTNKLTNNGTSIHWHGIRQLNNAQYDGVPGITQCPIAPGDTLTYKFHADN
YGSSWYHSHFILQYGDGLFGPLVINGPATANYDVDLGMLFLNDWNHVPVQSLWDKAKTGAPPTLLTGLMNGTNTYNGAGK
KFQTTFTPGKKYRIRVVNTAVDGHFQFSIDGHSFQVIAMDFVPIVPYNATSILVSIAQRYDIIVTANAAVGNYWIRAGWQ
TACSGNTNAANITGILRYTGSSSTADPTTTSTVTASTSCLDEPLASLVPFVPINPVASSIMKTTLTTGGGQWLFNGSSLL
LNWTDPTLLTVLNSGNIWPTEYNVIPVESTTANKGWAVLAISGPNGPNHPIHLHGHDFWTLSQGTGAYTATTALNLVNPP
RRDVMTLPSGGHLVIAFQIDNPGSWLMHCHIAWHASEGLALQFVESESKILPTIGTADVSTFQDTCAAWNAWTPTEPFPQ
DDSGI
>KAF7949645.1 hypothetical protein EAE97_003154 [Botrytis byssoidea]
MKNSFFSSLAKFSSLSLALALPTAEVIPSALEERQSCANTATTRSCWGQYSASTNSYTTVPQTGVTREYWLVVQNSTLSA
DGVSRPTLNFNGTIPGPQITADWGDNVIVHVTNKLTNNGTSIHWHGIRQLNNAQYDGVPGITQCPIAPGDTLTYKFHADN
YGSSWYHSHFILQYGDGLFGPLVINGPATANYDVDLGMLFLNDWNHVPVQLFWDKAKTGAPPTLLTGLMNGTNTYNGAGK
KFQKTFTPGKKYRIRVINTAVDGHFQFSIDGHSFQVIAMDFVPIVPYNATSILVSIAQRYDIIVTANAAVGNYWIRAGWQ
TACSGNTNAANITGILRYTGSSSTADPTTTSTVTASTSCLDEPLTSLVPFVPINPVASSIMKTTLTTGGGQWLFNGSSLL
LNWTDPTLLTVLNSGNIWPTEYNVIPVESTTANQGWAVLAISGPNGPNHPIHLHGHDFWTLSQGTGAYTATTALNLVNPP
RRDVMTLPSGGHLVIAFQIDNPGSWLMHCHIAWHASEGLALQFVESESKILPTIGTADVSTFQDTCAAWNAWTPTEPFPQ
DDSGI

>KAF7938180.1 hypothetical protein EAE99_001852 [Botrytis elliptica]
MKNSFFSSLAKFASLSLALALPTAEVVPTALEERQSCANTATTRSCWGQYSASTNSYTTVPQTGVTREYWLVVQNTTLSA
DGVSRPTLNFNGTIPGPQITADWGDDVIVHVTNKLTNNGTSIHWHGIRQLNNAQYDGVPGITQCPIAPGDTLTYKFHADN
YGSSWYHSHFILQYGDGLFGPLVINGPATANYDVDLGMLFLNDWNHVPVQSLWDKAKTGAPPTLLTGLMNGTNTYNGAGK
KFQTTFTPGLKYRIRVVNTAVDGHFQFSIDGHSFQVIAMDFVPIVPYNATSILVSIAQRYDIIVTANAAVGNYWIRAGWQ
TACSGNTNAANITGILRYTGSSSTADPTTTSTVTASTSCLDEPLTSLVPFVPINPVASSIMKTTLTTAGGQWLFNGSSLL
LNWTDPTLLTVLNSGNIWPTEYNVIPVESTTANKGWAVLAISGPNGPNHPIHLHGHDFWTLSQGTGAYTATTALNLVNPP
RRDVMSLPSGGHLVIAFQIDNPGSWLMHCHIAWHASEGLALQFVESESKILPTIGTADVSTFQDTCAAWNAWTPTEPFPQ
DDSGI
>TGO31276.1 hypothetical protein BPAE_0001g01740 [Botrytis paeoniae]
MKNSFFSSLAKFASLSLALALPTAEVIPSALEERQSCANTATTRSCWGQYSASTNSYTTVPQTGVTREYWLVVQNTTLSA
DGVSRPTLNFNGTIPGPQITADWGDDVIVHVTNKLTNNGTSIHWHGIRQLNNAQYDGVPGITQCPIAPGDTFTYKFHADN
YGSSWYHSHFILQYGDGLFGPLVINGPATANYDVDLGMLFLNDWNHVPVQSLWDKAKTGAPPTLLTGLMNGTNTYNGAGK
KFQTTFTPGLKYRIRVVNTAVDGHFQFSIDGHSFQVIAMDFVPIVPYNATSILVSIAQRYDIIVTANAAVGNYWIRAGWQ
TACSGNTNAANITGILRYTGSSSTADPTTTSTITASTSCLDEPLTSLVPFVPINPVASSIMKTTLTTGGGQWLFNGSSLL
LNWTDPTLLTVLNSGNIWPTEYNVIPVESTTANNGWAVLAISGPNGPNHPIHLHGHDFWTLSQGTGAYTATTALNLVNPP
RRDVMTLPSGGHLVIAFQIDNPGSWLMHCHIAWHASEGLALQFVESESNILPTIGTADVSAFQDTCAAWNAWTPTEPFPQ
DDSGI
>XP_037197222.1 putative laccase 2 protein [Botrytis fragariae]
MHTSSLLTALLKIPPTEYYSVLAQWSSGKGLLCVRGGERPHVRFRDATFSLRSADMETKDEDIVVVSEVQLIFTRKEKDI
GCVIGDISADNSLAKFASLSLALALPTAEVIPSALEERQSCANTATTRSCWGQYSASTNSYTTVPQTGVTREYWLVVQNT
TLSADGVSRPTLNFNGTIPGPQITADWGDDVIVHVTNKLTNNGTSIHWHGIRQLNNAQYDGVPGITQCPIAPGDTLTYKF
HADNYGSSWYHSHFILQYGDGLFGPLVINGPATANYDVDLGMLFLNDWNHVPVQSLWDKAKTGAPPTLLTGLMNGTNTYN
GAGKKFQTTFTPGLKYRIRVVNTAVDGHFQFSIDGHSFQVIAMDFVPIVPYNATSILVSIAQRYDIIVTANAAVGNYWIR
AGWQTTCSGNTNAANITGILRYTGSSSTADPTTISTITASTSCLDEPLTNLVPFVPINPVASSIMKTTLTTAGGQWLFNG
SSLLLNWTDPTLLTVLNSGNIWPTDYNVIPVESTTANKGWAVLAISGPTGPNHPIHLHGHDFWTLSQGTGAYTATTALNL
VNPPRRDVMTLPSGGHLVIAFQIDNPGSWLMHCHIAWHASEGLALQFVESESKILPTIGTADVSTFQDTCAAWNAWTPTE
PFPQDDSGI
>TGO18838.1 hypothetical protein BTUL_0007g00560 [Botrytis tulipae]
MKNSFFSSLAKFASLSLALALPTAEVIPFALEERQSCANTATTRSCWGQYSASTNSYTTVPQTGVTREYWLVVQNTTLSA
DGVSRPTLNFNGTIPGPQITADWGDDIIVHVTNKLTNNGTSVHWHGIRQLNNAQYDGVPGITQCPIAPGDTLTYKFHADN
YGSSWYHSHFILQYGDGLFGPLVINGPTTANYDVDLGMLFLNDWNHVPVQSLWDKAKTGAPPTLLTGLMNGTNTYNGAGK
KFQTTFTPGKKYRIRVVNTAVDGHFQFSIDGHSFQVIAMDFVPIVPYNATSILVSIAQRYDIIVTANAAVGNYWIRAGWQ
TACSGNTNAANITGILRYTGSSSTADPTTTSTVTASTSCLDEPLTSLVPFVPINPVASSIMKTTLTTGGGQWLFNGSSLL
LNWTDPTLLTVLNSGNIWPTEYNVIPVESTTANQGWAVLAISGPNGPNHPIHLHGHDFWTLSQGTGAYTATTALNLVNPP
RRDVMTLPSGGHLVITFQIDNPGSWLMHCHIAWHASEGLALQFVESESKILPTIGTTDVSTFQDTCAAWNAWTPTEPFPQ
DDSGI
>KAF7928481.1 hypothetical protein EAE98_005537 [Botrytis deweyae]
MKNPFFSSLAKFASLSLALALPTAEVVPSALEERQSCANTATTRSCWGQYSASTNSYTTVPQTGVTREYWLVVQNTTLSA
DGVSRPTLNFNGTIPGPQITADWGDDVIVHVTNKLTNNGTSIHWHGIRQLNNAQYDGVPGITQCPIAPGDTLTYKFHADN
YGSSWYHSHFILQYGDGLFGPLVINGPATANYDVDLGMLFLNDWNHVTVQSLWDKAKTGAPPTLLTGLMNGTNTYNGAGK
KFQTTFTPGLKYRIRVVNTAVDGHFQFSIDGHSFQVIAMDFVPIVPYNATSILVSIAQRYDIIVTANAAVGNYWIRAGWQ
TACSGNTNAANITGILRYTGSSSTADPTTTSTVTASTSCLDEPLTSLVPFVPINPVASSIMKTTLTTAGGQWLFNGSSLL
LNWTDPTLLTVLNSGNIWPTEYNVIPVESTTANKGWAVLAISGPNGPNHPIHLHGHDFWTLSQGTGAYTATTALNLVNPP
RRDVMTLPSGGHLVIAFQIDNPGSWLMHCHIAWHASEGLALQFVESESKILPTIGTADVSTFQDTCAAWNAWTPTEPFPQ
DDSGI
>XP_024546494.1 Bclcc1 [Botrytis cinerea B05.10]
MKNSFFSSLAKFASLSLALALPTAEVIPSALEERQSCANTATTRSCWGQYSASTNSYTTVPKTGVTREYWLVVQNTTLSA
DGVSRPTLNFNGTIPGPQITADWGDDVIVHVTNKLTSNGTSIHWHGIRQLNNAQYDGVPGITQCPIAPGGTLTYKFHADN
YGSSWYHSHFILQYGDGLFGPLVINGPATANYDVDLGMLFLNDWNHVPVQSLWDKAKTGAPPTLLTGLMNGTNTYNGAGK
KFQTTFTPGLKYRIRVVNTAVDGHFQFSIDGHSFQVIAMDFVPIVPYNATSILVSIAQRYDIIVTANAAVGNYWIRAGWQ
TACSGNTNAANITGILRYTGSSSTADPTTTSTVTASTSCLDEPLASLVPFVPINPVASSIMKTTLTTGGGQWLFNGSSLL
LNWTDPTLLTVLNSGNIWPTEYNVIPIESTTANKGWAVLAISGPNGPNHPIHLHGHDFWTLSQGTGAYTATTALNLVNPP
RRDVMTLPTGGHLVIAFQIDNPGSWLMHCHIAWHASEGLALQFVESESSILPTIGTADVSTFQNTCAAWKAWTPTEPFPQ
DDSGI
>TGO33572.1 hypothetical protein BHYA_0239g00120 [Botrytis hyacinthi]
MKNSFFSSLAKFASLSLALALPTAEVIPSALEERQSCANTATTRSCWGQYSASTNSYTTVPQTGVTREYWLVVQNTTLSA
DGVSRPTLNFNGTIPGPQITADWGDNVIVHVTNKLTNNGTSIHWHGIRQLNNAQYDGVPGITQCPIAPGDTLTYKFHADN
YGSSWYHSHFILQYGDGLFGPLVINGPATANYDVDLGMLFLNDWNHVPVQSLWDKAKTGAPPTLLTGLMNGTNTYNGAGK
KFQTTFTPGKKYRIRVVNTAVDGHFQFSIDGHNFQVIAMDFVPIIPYNATSILVSIAQRYDIIVTANAAVGNYWIRAGWQ
TACSGNTNAANITGILRYTGSSSTADPTTTSTVTASTSCMDEPLTSLVPFVPINPVASSIMKTKLTTGGGQWLFNGSSLL

LNWTDPTLLTVLNSGNIWPTEYNVIPVESTTANKGWAALAISGPNGPNHPIHLHGHDFWTLSQGTGTYTATTALNLVNPP
RRDVMTLPSGGHLVIAFQIDNPGSWLMHCHIAWHASEGLALQFVESESKILPTIGTADVSTFQDTCAAWNAWTPTEPFPQ
DDSGI
>XP_037194516.1 putative multicopper oxidase protein [Botrytis fragariae]
MHLPVLLRVLILVPFVLAAPKYGESSLTKRCVNSADDRSCWGDYDISTNYYNDGPDTGVVREYWWDIVNTTAAPDGIERV
ILSVNGSVPGPTIIADWGDTIVIHVKNSMPNYGTGIHFHGIRQNYTNQMDGVPSITQCPIAPGTSFTYTWKATQYGSSWY
HSHFYVQAWDGIFGGIQINGPATANYDEDLGTLTLSDWSHVTAEVEAVAALAAPPTLDNGLINGTNTYNNSGTIVGSRFE
TTFKSGTRYRIRLVNTAADTHFKFSIDNHTMEVIAADFVPIVPYTSDTIAISMGQRYDIIVTANAAVDNYWMRAVAQTVC
SKNVNADNIYGIVRYDSTSTDDPTSVRWANASTDELCEDELMTSLVPYVPIVASDSPAEEDEFSVIINKTNGVIWQMGTH
SFLNQWDYPTLLQSYEGNDTWAPEQEVYQLPVANVWVYWIIETTNAQSHPMHLHGHDFWVLGQGTGTYDSTTANLTLVNA
PRRDVVLLPGSGWVVMAFYTDNPGAWIMHCHIAWHTSEGLAVQILERESELVDLLDGDLLNSTCAAWNSYASADNVIQPD
SDAIPGKGESNLTFGLELEYIFATVDADKPDPHPEDPREVDGKKFPDKEAINRDILKKLTGVGIPAVITSSDMTDEESIT
CWILKEDTTVGDNTLRPAENKSKIYHRNGMEMTSPPYYYTEPARNAIRKVLKTVRENYRVCVDETAGLHVHVGNSFNGFH
FLILQHILAIAYTYERQTELIFSSDRVSGEWCPPFSKGRFGMANPDLTRVEVVEKILGYTDNESLIEDFGESLTAGRLAF
NLEGLKTPYQDGIRTIEFRHHQGSLDPGAILNWIHVCVKLVEKACFAKHDELLAQLRQDIAKPIGFGEGDLSTIDFLMWL
GCPAQAYYYCANMVIDKNTFEQRIKDDTARQEKSLKWARARLIRLAKDAEEHEARVAARLGKDGSKESEPTTSNSESADE
NEADQDEGGNDGSGDSGNTPSNPNEDES
>KAF7893007.1 hypothetical protein EAF02_000545 [Botrytis sinoallii]
MENSFFSSFAKFASLSLALALPTAEVVPSALEERQSCANTATTRSCWGQYSASTNSYTTVPQTGVTREYWLVVQNTTLSA
DGVSRPTLNFNGTIPGPQITADWGDDVIVHVTNKLTNNGTSIHWHGIRQLNNAQYDGVPGITQCPIAPGDTLTYKFHADN
YGSSWYHSHFILQYGDGLFGPLVINGPATANYDVDLGMLFLNDWNHDKAKTGAPPTLLTDLMNGTNTYNGAGKKFQTTFT
PGSKYRIRVVNTAVDGHFQFSIDGHSFQVIAMDFVPIVPYNATSILVSIAQRYDIIVTANAAVGNYWIRAGWQTACSGNT
NAANITGILRYTGSSSTADPTTTSTVTASTSCLDEPLTSLVSFVPINPVASSIMKTTLTTAGGQWLFNGSSLLLNWTDPT
LLTVLNSGNIWPTEYNVIPVESTTANKGWAVLAISRPNGPNHPIHLHGHDFWTISQGTGAYTATTALNLVNPPRRDVMTL
PSGGHLVIAFQIDNPGSWLMHCHIAWHASEGLALQFVESESKILPTIGTADVSTFQDTCAAWNAWTPTEPFPQDDSGI
>Q12570.3 RecName: Full=Laccase-1; AltName: Full=Benzenediol:oxygen oxidoreductase 1; AltName: Full=Diphenol oxidase 1; AltName: Full=Urishiol oxidase 1; Flags: Precursor [Botrytis cinerea]
MKNSFFSSLAKFASLSLAFALPTAEVIPSALEERQSCANTATTRSCWGQYSASTNSYTTVPKTGYWLVVQNTTLSADGVS
RPTLNFNGTIPGPQITADWGDDVIVHVTNKLTSNGTSIHWHGIRQLNNAQYDGVPGITQCPIAPGGTLTYKFHADNYGSS
WYHSHFILQYGDGLFGPLVINGPATANYDVDLGMLFLNDWNHVPVQSLWDKAKTGAPPTLLTGLMNGTNTYNGAGKKFQT
TFTPGLKYRIRVVNTAVDGHFQFSIDGHSFQVIAMDFVPIVPYNATSILVSIAQRYDIIVTANAAVGNYWIRAGWQTACS
GNTNAANITGILRYTGSSSTADPTTTSTVTASTSCLDEPLASLVPFVPINPVASSIMKTTLTTGGGQWLFNGSSLLLNWT
DPTLLTVLNSGNIWPTEYNVIPIESTTANKGWAVLAISGPNGPNHPIHLHGHDFWTLSQGTGAYTATTALNLVNPPRRDV
MTLPTGGHLVIAFQIDNPGSWLMHCHIAWHASEGLALQFVESESSILPTIGTADVSTFQNTCAAWKAWTPTEPFPQDDSG
I
>TGO29104.1 hypothetical protein BPAE_0019g00800 [Botrytis paeoniae]
MRLHILLALCLSFCQIQASPLFGWSFNPIATLQTDLLKIINQVECLVSTKVSIIPSILTTGNTNAVTSNKCSTTSGKTTS
AAAVQTSLSSLQSVASSKWSSLQSLLSTTTSLPPILTTSLTVTTAKPTSTSSVKSTSTTANTVSSTTKSSSITSTTSLVS
SSTASSTSKTSSSAVASGTPCAGNTAADRSKWCDYSTSTDYYNEVPNTGVTREYWFNVQDGVASPDGFSRYVQAINGSIP
GPTIIADWGDNIVVHVTNSLSTNGSSIHFHGIHQKNTNQNDGVPSVTQCPIAYGDTYTYRWRATQYGSSWYHSHVGLQAW
EGVAGGIIINGPATANYDEDKGTLMLSDWGHETVDELYEKVQTTGPQSMTTGLINGTNVFGADGASNQTGSRFSTSVESG
KSYRFRLVNSAIDTHFKFSVDSHTMTVMAMDFVPIVPYQTEILNIAIGQRYDVVITANQASVASDFFIRAIPQSSCSEND
NSDNIRGVLHYGSSTGLPTTTGYTFTDECVDEPVSSLVPYLPKTVSAASKSPEEAVTVAQNSQNLFKWYLNNSTFLSEWE
EPTLLMVQNNITTFGTPDNLVAVPNANEWIYLVIHSALPVPHPIHLHGHDFFVVSQQATTFDTTTAISTYNLNNPPRRDV
ATLPGGGYLVLAFETDNPGAWLMHCHIGWHTSMGFAMQFLERASEIPKLLDTTQLQDTCTAWNGHAVIQEDSGV
>TGO32268.1 hypothetical protein BHYA_0333g00040 [Botrytis hyacinthi]
MRLHILLALCLSFCQIQASPLFGWSFNPIATLQADLLKIINQVECLASTKVSYIPSLLTFGNTNAVTSNKCSTTSGKTTS
AAAVQTSLSSLQSVASSKWSSLQSLLSTTTSLPPIFTSSLAVTTAKPTSTSSATSTSTTANTVSSTAKSSTITGTTSPAS
SSTASSTSKTSSSAVASGTPCAGNTAADRSKWCDYSTSTDYYNEVPNTGVTREYWFNVQDGVASPDGVSRYVQTINGSIP
GPTIIADWGDNVVVHVTNSLSTNGSTIHFHGIHQKNTNQNDGVPSVTQCPIAYGNTYTYRWRATQYGSSWYHSHVGLQAW
EGVAGGIIINGPATANYDEDKGTLMLSDWGHQTVDELYEKVQTVGPQSMTTGLINGTNVFGADGASNQTGSRFSTSVESG
KSYRFRLVNSAIDSQFKFSVDSHTMTVMAMDFVPIVPYQTNILNIAIGQRYDVVITANQASVASDFFIRAIPQSSCSEND
NSDNIRGVLHYGSSTGLPTTTGYTFTDECVDEPVSSLVPYLPKTVSAASKSPEEAVTVAQNSQKLFKWYLNNSTFLSEWK
DPTLLMVQNNITAFGTPDNLVEVPNANEWIYLVIHSALPVPHPIHLHGHDFFVVSQQATAFDATTAVSTYNLNNPPRRDV
ATLPGGGYLVLAFETDNPGAWLMHCHIGWHTSMGFAMQFLERASEIPKLLDTTQLQDTCTAWTGHAVIQEDSGV
>XP_001547254.1 Bclcc8 [Botrytis cinerea B05.10]
MRLHILLALCLSFCQIHASPLFGWSFNPIATLQADLLKIINQAECLVATKVSIIPSILTFGNSNPVTSNKCSTTSGKTTS
SAAIQTSLSSLQSVASSKWSSLQSLLSTTTSLPPILTTSFTATTAKPTTTSSVKSTSTTANTVSSTAKPSSITSTASSTA
SSTSKTSSSAVASGTPCAGNTAADRSKWCNYSTSTDYYNEVPDTGVTREYWFNVQDGVASPDGISRYVQTINGSIPGPTI
IADWGDNVVVHVTNSLTTNGSSIHFHGIHQKNTNQNDGVPSVTQCPIAYGDTYTYRWRATQYGSSWYHSHVGLQAWEGVT
GGIIINGPATANYDEDKGTLMLSDWGHETVDELYQKVQTVGPQSMTTGLINGTNVFGADGASNQTGSRFSTSVQSGKSYR

FRLVNSAIDSQFKFSVDSHTMTVMAMDFVPIVPYQTEILNIAIGQRYDVVITANQASVASDFFIRAIPQSSCSENDNSDN
IRGVLHYGSSTGLPTTTGYTFTDECVDEPVASLVPYLPKTVSSASKSPEEAVTVAQNSQKLFKWYLNNSTFLSEWEDPTL
LMVQNNVTAFGTPDNLVEVPNANEWIYLVIHSALPVPHPIHLHGHDFFVVSQQSTTFDATTAVSTYNLNNPPRRDVATLP
GGGYLVLAFETDNPGAWLMHCHIGWHTSMGFAMQFLERASEIPKLLDTTQMLDTCTAWNGHAIIQEDSGV
>KAF7892221.1 hypothetical protein EAF01_010301 [Botrytis porri]
MKYFTVFTALTVFFAQASASAIPAVRSPLTPRQNTTASCANSATSRSCWGEYSIDTNWYDVTPTGVTREYWLSVENSTIK
PDGYTRSAMTFNGTVPGPAIIADWGDNLIIHVTNNLEYNGTSIHWHGIRQLGSLEYDGVPGVTQCPIAPGDTLTYKFQVT
QYGTTWYHSHFSLQYGDGLFGPLIINGPATADYDEDAGVIFLQDWAHESVFEIWDSARIGAPPALENTLMNGTNTFDCSA
STDPNCVGGGKKFELTFVEGTKYRLRLINVGIDSHFEFAIDGHTLTVIATDLVPIVPYTTETLLIGIGQRYDVIVEANAA
AGNYWIRGNWGNACSANNEAANATGILRYDSSSTADPTSVGTTPRGTCEDEPVASLVPHLALNVGDYSLVDEQVSFAFTN
YFTWTINSSSLLLDWSSPTTLKIFNNETIFPTDYNVVPLEQTNNEEWVVYVIEDLTGFGIWHPIHLHGHDFFIVAQEADV
FNADKSPAKFNLVNPPRRDVAALPGNGYLAIAFKLDNPGSWLLHCHIAWHASEGFAMQFVESQSSIAVGMTDTAIFENTC
ANWNAYTPTQLFAEDDSGI
>XP_001553186.1 Bclcc2 [Botrytis cinerea B05.10]
MKYSTVFTALTALFAQASATAIPAVRSPLAPRQSTTASCANSATSRSCWGEYSIDTNWYDVTPNTGVTREYWLSVENSTI
TPDGYTRSAMTFNGTVPGPAITADWGDNLIIHVTNNLQHNGTSIHWHGIRQLGSLEYDGVPGVTQCPIAPGDTLTYKFQA
TQYGTTWYHSHFSLQYADGLFGPLIINGPATADYDEDVGAIFLQDWAHKSVFEIWDSARQGAPPALENTLMNGTNIYDCS
ASTDANCVGGGKKFELTFVEGTKYRLRLINVGIDSHFEFAIDNHTLTVIANDLVPIVPYTTDTLLIGIGQRYDVIVEANA
AADNYWIRGNWGTTCSSNSEAANATGILRYDSSSTVDPTSVGVTPRGTCADEPVASLVPHLALDVGGYSLVDEQVSFAFT
NYFTWTINSSSLLLDWSSPTTLKIFNNETIFPTDYNVVALNQTDANEEWVVYVIEDLTGFGIWHPIHLHGHDFYVVAQET
DVFSATKSPANFNLVNPPRRDVAALPGNGYLAIAFKLDNPGSWLLHCHIAWHASEGLAMQFVESQSSIAIGMSDTDIFED
TCANWNAYTPTELFAEDDSGI
>TGO86421.1 hypothetical protein BPOR_0305g00070 [Botrytis porri]
MKYFTVFTALTVFFAQASASAIPAVRSPLTPRQNTTASCANSATSRSCWGEYSIDTNWYDVTPTGVTREYWLSVENSTIK
PDGYTRSAMTFNGTVPGPAIIADWGDNLIIHVTNNLEYNGTSIHWHGIRQLGSIEYDGVPGVTQCPIAPGDTLTYKFQVT
QYGTTWYHSHFSLQYGDGLFGPLIINGPATADYDEDAGVIFLQDWAHESVFEIWDSARIGAPPALENTLMNGTNTFDCSA
STDPNCVGGGKKFELTFVEGTKYRLRLINVGIDSHFEFAIDGHTLTVIATDLVPIVPYTTETLLIGIGQRYDVIVEANAA
AGNYWIRGNWGNACSANNEAANATGILRYDSSSTADPTSVGTTPRGTCEDEPVASLVPHLALNVGDYSLVDEQVSFAFTN
YFTWTINSSSLLLDWSSPTTLKIFNNETIFPTDYNVVPLEQTNNEEWVVYVIEDLTGFGIWHPIHLHGHDFFIVAQEADV
FNADKSPAKFNLVNPPRRDVAALPGNGYLAIAFKLDNPGSWLLHCHIAWHASEGFAMQFVESQSSIAVGMTDTAIFENTC
ANWNAYTPTQLFAEDDSGI
>AFC76164.1 laccase [Botrytis aclada]
MKYFTVFTALTALFAQASASAIPAVRSTLTPRQNTTASCANSATSRSCWGEYSIDTNWYDVTPTGVTREYWLSVENSTIT
PDGYTRSAMTFNGTVPGPAIIADWGDNLIIHVTNNLEHNGTSIHWHGIRQLGSLEYDGVPGVTQCPIAPGDTLTYKFQVT
QYGTTWYHSHFSLQYGDGLFGPLIINGPATADYDEDVGVIFLQDWAHESVFEIWDTARLGAPPALENTLMNGTNTFDCSA
STDPNCVGGGKKFELTFVEGTKYRLRLINVGIDSHFEFAIDNHTLTVIANDLVPIVPYTTDTLLIGIGQRYDVIVEANAA
ADNYWIRGNWGTTCSTNNEAANATGILRYDSSSIANPTSVGTTPRGTCEDEPVASLVPHLALDVGGYSLVDEQVSSAFTN
YFTWTINSSSLLLDWSSPTTLKIFNNETIFPTEYNVVALEQTNANEEWVVYVIEDLTGFGIWHPIHLHGHDFFIVAQETD
VFNSDESPAKFNLVNPPRRDVAALPGNGYLAIAFKLDNPGSWLLHCHIAWHASEGLAMQFVESQSSIAVKMTDTAIFEDT
CANWNAYTPTQLFAEDDSGI
>KAF7924682.1 hypothetical protein EAE97_010633 [Botrytis byssoidea]
MRLHILLALCLSFCQIQASPLFGWSFNPIATLQADLLKIINQVECLASTKVSYIPSLLTFGNTNAVTSNKCSTTSGKTTS
AAAVQTSLSSLQSVASSKWSSLQSLLSTTTSLPPIFTSSITVTTANPTSISSATSTSTTANTVSSTAKSSSITSTTSPVS
SSTASSTSKTSSSAVASGTPCAGNTAADRSKWCDYSTSTDYYNEVPNTGVTREYWFNVQDGVASPDGVSRYVQTINGSIP
GPTIIADWGDNVVVHVTNSLATNGSTIHFHGIHQKNTNQNDGVPSVTQCPIAYGDTYTYRWRATQYGSSWYHSHVGLQAW
EGVAGGIIINGPATANYDEDKGTLMLSDWGHVTVDELYQKIQTIGPQSMATGLINGTNVFGADGASNQTGSRFSTSVESG
KSYRFRLVNSAIDSQFKFSVDSHTMTVMAMDFVPIVPYQTEILNIAIGQRYDVVITANQASVASDFFIRAIPQSSCSEND
NSDNIRGVLHYGSSTGLPTTTGYTFTDECVDEPVSSLIPYLPKTVSAASKSPEEAVTIAQNSQKLFKWYLNNSTFLSEWE
DPTLLMVQNNITAFGTPDNLVEVPNANEWIYLVIHSALPVPHPIHLHGHDFFVVSQQATTFDAATAVSTYNLNNPPRRDV
ATLPGGGYLVLAFETDNPGAWLMHCHIGWHTSMGFAMQFLERASEIPKLLDTKQLQDTCTAWTGHAVIQEDSGV
>3V9E_A Structure of the L499M mutant of the laccase from B.aclada [Botrytis aclada]
MKYFTVFTALTALFAQASASAIPAVRSTLTPRQNTTASCANSATSRSCWGEYSIDTNWYDVTPTGVTREYWLSVENSTIT
PDGYTRSAMTFNGTVPGPAIIADWGDNLIIHVTNNLEHNGTSIHWHGIRQLGSLEYDGVPGVTQCPIAPGDTLTYKFQVT
QYGTTWYHSHFSLQYGDGLFGPLIINGPATADYDEDVGVIFLQDWAHESVFEIWDTARLGAPPALENTLMNGTNTFDCSA
STDPNCVGGGKKFELTFVEGTKYRLRLINVGIDSHFEFAIDNHTLTVIANDLVPIVPYTTDTLLIGIGQRYDVIVEANAA
ADNYWIRGNWGTTCSTNNEAANATGILRYDSSSIANPTSVGTTPRGTCEDEPVASLVPHLALDVGGYSLVDEQVSSAFTN
YFTWTINSSSLLLDWSSPTTLKIFNNETIFPTEYNVVALEQTNANEEWVVYVIEDLTGFGIWHPIHLHGHDFFIVAQETD
VFNSDESPAKFNLVNPPRRDVAALPGNGYLAIAFKLDNPGSWLLHCHIAWHASEGMAMQFVESQSSIAVKMTDTAIFEDT
CANWNAYTPTQLFAEDDSGI
>THV50112.1 hypothetical protein BGAL_0164g00030 [Botrytis galanthina]
MRLHILLALCLSFCQIQASPLFGWSFNPIATLQADLLKIINQVECLASTKVSYIPSLLTFGNTNAVTSNKCSTTSGKTTS

AAAVQTSLSSLQSVASSKWSSLQSLLSTTTSLPPIFTSSLTVTTAKPTSTSSVASTSTTANTMSPTAKSSSITSTTSPVS
SSTASSTSKTSSSAVASGTPCAGNTAADRSKWCDYSTSTDYYNEVPNTGVTREYWFNVQDGVASPDGVSRYVQTINGSIP
GPTIIADWGDNVVVHVTNSLATNGSTIHFHGIHQKNTNQNDGVPSVTQCPIAYGDTYTYRWRATQYGSSWYHSHVGLQAW
EGVAGGIIINGPATANYDEDKGTLMLSDWGHVTVDELYQKIQTIGPQSMATGLINGTNVFGADGASNQTGSRFSTSVESG
KSYRFRLVNSAIDSQFKFSVDSHTMTVMAMDFVPIVPYQTEILNIAIGQRYDVVITANQASVASDFFIRAIPQSSCSEND
NSDNIRGVLHYGSSTGLPTTTGYTFTDECVDEPVSSLVPYLPKTVSAASKSPEEAVTIAQNSQKLFKWYLNNSTFLSEWE
DPTLLMVQNNITAFGTPDNLVEVPNANEWIYLVIHSALPVPHPIHLHGHDFFVVSQQATTFDAATAVSTYNLNNPPRRDV
ATLPGGGYLVLAFETDNPGAWLMHCHIGWHTSMGFAMQFLERASEIPKLLDTTQLQDTCTAWTGHAVIQEDSGV
>XP_037193984.1 putative extracellular dihydrogeodin oxidase protein [Botrytis fragariae]
MKYSTLFTALTAFFAQASASAIPAVRSPLTPRQNTTASCANSATSRSCWGEYSIDTNWYDVTPTGVTREYWLSVENSTIA
PDGYTRSAMTFNGTVPGPAIIADWGDNLVIHVTNNLEYNGTSIHWHGIRQLGSLEYDGVPGVTQCPIAPGDTLTYKFQVT
QYGTTWYHSHFSLQYGDGLFGPLIINGPATADYDEDVGAIFLQDWAHESVFEIWDSAKLGGPPALENNLMNGTNIFDCSA
STDPNCVGGGKKFELTFVEGTKYRLRLINVGIDSHFEFAIDGHTLTVIANDLVPIVPYTTDTLLIGIGQRYDVIVEANAA
ADNYWIRGNWGTACSANNEAANATGILRYDSSSTADPTSVGTTPRGTCEDEPVASLVPHLALDVGGYSLVDEQLSFAFTN
YFTWTINSSSLLLDWSSPTTLKIFNNETIFPTDYNVVPLEQTSANEEWVVYVIEDLTGFGIWHPIHLHGHDFYIVAQEAD
VFDPDTSPAEFNLVNPPRRDVAALPGNGYLAIAFKLDNPGSWLLHCHIAWHASEGLAMQFVESQSSIAVGMSDTAIFEDT
CANWNAYTPTQLFAEDDSGI
>KAF7947057.1 hypothetical protein EAE97_004306 [Botrytis byssoidea]
MKCSTIFTALTAFFAQASASAIPAVRSPLTPRQNTTTSCANSATSRSCWGEYSIDTNWYDVTPTGVTREYWLSVENSTIT
PDGYTRSAMTFNGTVPGPAIIADWGDNLIIHVTNNLEHNGTSIHWHGIRQLGSLEYDGVPGVTQCPIAPGDTLTYKFQAT
QYGTTWYHSHFSLQYGDGLFGPLIINGPATADYDEDVGVIFLQDWAHESVFEIWDSARLGAPPALENTLMNGTNTFDCSA
STDPNCVGGGKKFELTFVEGTKYRLRLINVGIDSHFEFAIDGHTLTVIANDLVPIVPYTTDTILIGIGQRYDVIVEANAA
ADNYWIRGNWGTTCSGNMEAANATGILRYDSSSTADPTSVETTPRGTCEDEPVASLVPHLALDVGGYSLVDEQMSFAFTN
YFTWTINSSSLLLDWSSPTTLKIFNNETIFPTEYNVVPLEQTNANEEWVVYVIEDLTGFGIWHPIHLHGHDFFIVAQEAD
VFNPDKSPAKFNLVNPPRRDVAALPGNGYLAIAFKLDNPGSWLLHCHIAWHASEGLAMQFVESQSSIAVGMSDTAIFENT
CANWNAYTPTQLFAEDDSGI
>KAF7914060.1 hypothetical protein EAF01_000466 [Botrytis porri]
MRLHILLALCLSLCQIQASPLFGWSFNPIATLQADLLKIINQVECLVSTKVSIIPSILTSGNTNAVTSNKCSTTSGKTTS
AAAIQTSLSSLQSVASSKWSSLQSLLSTTTSLPPILTTSLVTSTAKPTITSSVQSTSTTANTVSSTAKSSSITSTTSPVS
SSTSPSTSKTSSSAVASGTPCAGNTAADRSKWCDYSTSTDYYNEVPNTGVTREYWFNVQDGVASPDGFSRYVQTINGSIP
GPTIIADWGDNVVVHVTNSLSANGSSIHFHGIHQKNTNQNDGVPSVTQCPIAYGTTYTYRWRATQYGSSWYHSHVGLQAW
EGVTGGIIINGPATANYDEDKGILMLSDWGHQTVDELYEKVQTIGPQSMTTGLINGTNVFGEDGASNQTGSRFATSVESG
KSYRFRLVNSAIDTQFKFSVDSHTMTVMAMDFVPIVPYQTEILNIAIGQRYDVVITANQASVASDFFIRAIPQSSCSEND
NSNNIRGVLHYGSSTGLPTTTGYTFTDECVDEPVSSLVPYLPKTVSAASQSPEEAVTVAQNSQKLFKWYLNNSTFLSEWN
DPTLLMVENNITAFGTPDNLVAIPNANEWIYIVIHSALPVPHPIHLHGHDFFVVSQQATTFDAATAVSTYNLNNPPRRDV
ATLPGGGYLVLAFETDNPGAWLMHCHIGWHTSMGFAMQFLERASEIPKLLDTTQLQDTCAAWTGHAVIQEDSGV
>TGO30877.1 hypothetical protein BPAE_0003g01630 [Botrytis paeoniae]
MKYSTVFTVLTAFFAQASASAIPAVRSPLTPRQNTTASCANSATSRSCWGEYSIDTNWYDVTPTGVTREYWLSVENSTIT
PDGYTRSAMTFNGTVPGPAIIADWGDNLIIHVTNNLENNGTSIHWHGIRQLGSLEYDGVPGVTQCPIAPGDTLTYKFQVT
QYGTTWYHSHFSLQYGDGLFGPLIINGPATADYDEDVGVIFLQDWAHESVFEIWDSARLGAPPALENTLMNGTNTFDCSA
STDPNCVGGGKKFELTFVEGTKYRLRLINVGIDSHFEFAIDGHTLTVIANDLVPIVPYTTDTLLIGIGQRYDVIVEANAA
ADNYWIRGNWGTACSGNTEAANATGILRYDSSSTVDPTSVGTTPRGTCEDEPVASLVPHLALDVGGYSLVDEQMSFAFTN
YFTWTINSSSLLLDWSSPTTLKIFNNEKIFPTEYNVVPLEQTNANEEWVVYVIEDLTGFGIWHPIHLHGHDFFIVAQEAD
VFNPEKSPAKFNLVNPPRRDVAALPGNGYLAIAFKLDNPGSWLLHCHIAWHASEGLAMQFVESQSSIAVGMSDTAIFEDT
CANWNAYTPTQLFAEDDSGI
>TGO11347.1 hypothetical protein BTUL_0112g00370 [Botrytis tulipae]
MRLHILLALCLSFCQIQASPLFGWSFNPIATLQADLLEIVNQVECLASTKISYIPSLLTFGNTNTVTPNKCSPTSGKTTS
AAAVQTSLSSLQSVASSKWSSLQSLLSTTTSLPPILTSSLTVTTAKPTSTSSATSTSTTANTVSSTAKSSSITSTTSPVS
SSTASSTSKTSSSAVASGTPCAGNTAADRSKWCDYSTSTDYYNEVPNTGVTREYWFNVQDGVASPDGVSRYVQTINGSIP
GPTIIADWGDNVVVHVTNSLATNGSTIHFHGIHQKNTNQNDGVPSVTQCPIAYGDTYTYRWRATQYGSSWYHSHVGLQAW
EGVAGGIIINGPATANYDEDKGTLMLSDWGHVTVDELYQKIQTIGPQSMATGLINGTNVFGADGASSQTGSRFSTSVESG
KSYRFRLVNSAIDSQFKFSVDSHTMTVMAMDFVPIVPYQTEILNIAIGQRYDVVITANQASVASDFFIRAIPQSSCSEND
NSDNIRGVLHYGSSIGLPKTTGYTFIDECVDEPVSSLVPYLPKTVSAASKSPEEAVTVAQNSQKLFKWYLNNSTFLSEWK
DPTLLMVQNNITAFGTPDNLVVLPNANEWIYLVIHSALPVPHPIHLHGHDFFVVSQQATAFDAATAVSTYNLNNPPRRDV
ATLPGGGYLVLAFETDNPGAWLMHCHIGWHTSMGFAMQFLERASEIPKLLDTKQLQDTCTAWTGHTVIQEDSGV
>KAF7857155.1 hypothetical protein EAF02_011388 [Botrytis sinoallii]
MRLHILLALCLSFCQIQASPLFGWFFNPIATLQADLLNIINQAECLVSTKVSYIPSILTSGNTNAVTSNKCSTTSGKTTS
AAAVQSSLSSLQSVASSKWSSLQSLLSTTTSLPPILTTSLTTSTAKPTSTSSVTSTSTTANTVSSTAKPSSITSTTSPVS
SSTASSTSKTSSSAVASGTPCAGNTAADRSKWCDYSTSTDYYNEVPDTGVTREYWFNVQDGVASPDGFSRYVQTINGSIP
GPTIIADWGDDVVVHVTNSLATNGSTIHFHGIHQKNTNQNDGVPSVTQCPIAYGNTYTYRWRATQYGSSWYHSHVGLQAW
EGVAGGIIINGPATANYDEDKGTLMLSDWGHETVDELYQKVQTVGPQSMTTGLINGTNVFGADGASNQTGSRFSTSVESG

KSYRFRLVNSAIDSQFKFSVDSHTMTVMAMDFVPIVPYQTDILNIAIGQRYDVVITANQASVASDFFIRAIPQSSCSEND
NSDNIRGVLHYGSSTGLPTTTGYTFTDECVDEPVSSLVPYLPKTVSAASKSPEEAVTVAQNSQQLFKWYLNNSTFLSEWE
DPTLLMVQNNITAFGTPDNLVEVPNANEWIYLVIHSALPVPHPIHLHGHDFFVVSQQATTFDATTAISTYNLNNPPRRDV
ATLPGGGYLVLAFETDNPGAWLMHCHIGWHTSMGFAMQFLERASEIPKLLDTTQLQDTCAAWTGHAVIQEDSGV
>KAF7883499.1 hypothetical protein EAF02_005419 [Botrytis sinoallii]
MKYSTVFTALTAFFAQVSASAIPVVRSPLTPRQNTTASCANSATSRSCWGEYSIDTNWYDVTPTGVTREYWLSVENSTIT
PDGYTRSAMTFNGTVPGPAIIADWGDNLIIHVTNNLEYNGTSIHWHGIRQLGSLEYDGVPGVTQCPIAPGDTLTYKFQAT
QYGTTWYHSHFSLQYGDGLFGPLIINGPATADYDEDVGAIFLQDWAHESVFEIWDSARLGAPPALENTLMNGTNIFDCSA
STDPNCVGGGKKFELTFVEGTKYRLRLINVGIDSHFEFAIDGHTLTVIANDLVPIVPYTTDTLLIGIGQRYDVIVEANAA
ADNYWIRGNWGTTCSANMEAANATGILRYDSSSTADPTSVGTTPRGTCEDEPVASLVPHLALDVGAYSLVDEEVSFAFTN
YFTWTINSSSLLLDWSSPTTLKIFNNETIFPTEYNVVPLEQTNNEEWVVYVIEDLTGFGIWHPIHLHGHDFYIVAQEADV
FNPKTSPAKFNLVNPPRRDVAALPGNGYLAIAFKLDNPGSWLLHCHIAWHASEGLAMQFVESQSSIAVGMSDTAIFEDTC
ANWNAYTPTQLFAEDDSGI
>KAF7918470.1 hypothetical protein EAE98_009713 [Botrytis deweyae]
MRLHILLALCLSFCQIQASPLFGWFFNPIATLQADLLNIINQAECLVSTKVSYIPSILTSGNTNAVTSNKCSTTSGKTTS
AAAVQSSLSSLQSVASSKWSSLQSLLSTTTSLPPILTTSLTTSTAKPTSTSSVTSTSTTANTVSSTAKPSSITSTTSPVS
SSTASSTSKTSSSAVASGTPCAGNTAADRSKWCDYSTSTDYYNEVPDTGVTREYWFNVQDGVASPDGFSRYVQTINGSIP
GPTIIADWGDNIVVHVTNSLATNGSTIHFHGIHQKNTNQNDGVPSVTQCPIAYGNTYTYRWRATQYGSSWYHSHVGLQAW
EGVAGGIIINGPATANYDEDKGTLMLSDWGHQTVDELYQKVQTVGPQSMTTGLINGTNVFGADGASNQTGSRFSTSVESG
KSYRFRLVNSAIDSQFKFSVDSHTMTVMAMDFVPIVPYQTDILNIAIGQRYDVVITANQASVASDFFIRAIPQSSCSEND
NSDNIRGVLHYGSSTGLPTTTGYTFTDECVDEPVSSLVPYLPKTVSAASKSPEEAVTVAQNSQKLFKWYLNNSTFLSEWE
DPTLLMVQNNITAFGTPDNLVEVPNANEWIYLVIHSALPVPHPIHLHGHDFFVVSQQATTFDASTAISTYNLNNPPRRDV
ATLPGGGYLVLAFETDNPGAWLMHCHIGWHTSMGFAMQFLERASEIPKLLDTTQLQDTCAAWTGHAVIQEDSGV
>KAF7926490.1 hypothetical protein EAE99_005685 [Botrytis elliptica]
MRLHILLALCLSFCQIQASPLFGWFFNPIATLQADLLNIINQAECLVSTKVSYIPSILTSGNTNAVTSNKCSTTSGKTTS
AAAVQSSLSSLQSVASSKWSSLQSLLSTTTSLPPILTTSLTTSTAKPTSTSSVTSTSTTANTVSSTAKPSSITSTTSPVS
SSTASSTSKTSSSAVASGTPCAGNTAADRSKWCDYSTSTDYYNEVPDTGVTREYWFNVQDGVASPDGFSRYVQTINGSIP
GPTIIADWGDNVVVHVTNSLATNGSTIHFHGIHQKNTNQNDGVPAVTQCPIAYGNTYTYRWRATQYGSSWYHSHVGLQAW
EGVAGGIIINGPATANYDEDKGTLMLSDWGHQTVDELYQKVQTVGPQSMTTGLINGTNVFGADGASNQTGSRFSTSVESG
KSYRFRLVNSAIDSQFKFSVDSHTMTVMAMDFVPIVPYQTDILNIAIGQRYDVVITANQASVASDFFIRAIPQSSCSEND
NSDNIRGVLHYGSSTGLPTTTGYTFTDECVDEPVSSLVPYLPKTVSAASKSPEEAVTVAQNSQKLFKWYLNNSTFLSEWE
DPTLLMVQNNITAFGTPDNLVEVPNANEWIYLVIHSALPVPHPIHLHGHDFFVVSQQATTFDASTAISTYNLNNPPRRDV
ATLPGGGYLVLAFETDNPGAWLMHCHIGWHTSMGFAMQFLERASEIPKLLDTTQLQDTCAAWTGHAVIQEDSGV
>KAF7960387.1 hypothetical protein EAE96_000070 [Botrytis aclada]
MRLHILLALCLSICQIQATPLFGWSFNPIATLQADLLKIINQVECLASTKLSVIPSILTFGNTNAVTSNKCSTTAGKTTS
AAAVQTSLSSLQSVASSKWSSLQSLLSTTTSLPPILTSSLTVTTTKPTTTSSVQSTSITASTVSSTAKSSSITGTTSPVS
SSTASSTSKTSSSAVASGIPCAGNTAADRSKWCDYSTSTDYYNEVPDTGVTREYWFNVEDGVASPDGFSRYVQTINGSIP
GPTIIADWGDNIVVHVTNSLATNGSTIHFHGIHQKNTNQNDGVPSVTQCPIAYGSTYTYRWRATQYGSSWYHSHVGLQAW
EGVAGGIIINGPATANYDEDKGTLMLSDWGHETVDALYQKVQTVGSQSMTTGLINGTNVFGADGASNQTGSRFSTSVEAG
KSYRFRLVNSAIDSQFKFSVDSHTMTVMAIDFVPIVPYQTEILNIAIGQRYDVVITANKASVASDFFIRAMPQSSCSEND
NSDNIRGVLHYGSSIGLPTTTAYTFTDECVDEPASSLVPYLPKTVSAASKSPEEAVTIAQNSQKLFKWYLNNSTFLSEWE
DPTLLMVQNNITTFGTPDNLVAVPNANEWIYLVIHSALPVPHPIHLHGHDFFVVSQQTTTFDAATAVSTYNLNNPPRRDV
ATLPGGGYLVLAFETDNPGAWLMHCHIGWHSSMGFAMQFLERASEIPKLLDTTQLQDTCAAWNGHAVIQEDSGV
>TGO42259.1 hypothetical protein BHYA_0010g00190 [Botrytis hyacinthi]
MKYSTVFTALTALFAQASASAIPAARSPLTPRSPLTPRSPLTPRSPLTTRQNTTASCANSATSRSCWGEYSIDTNWYDVT
PTGVTREYWLSVENSTITPDGYTRSAMTFNGTVPGPAIIADWGDNLIIHVTNNLENNGTSIHWHGIRQLGSLEYDGVPGV
TQCPIAPGDTLTYKFQVTQYGTTWYHSHFSLQYGDGLFGPLIINGPATADYDEDVGAIFLQDWAHESVFEIWDSARLGAP
PALENTLMNGTNIFDCSASTDPNCVGGGKKFELTFVEGTKYRLRLINVGIDSHFEFAIDGHTLTVIANDLVPIVPYTTDT
LLIGIGQRYDVIVEANAAADNYWIRGNWGTACSGNLEAANATGILRYDSSSTADPTSVGTTPRGTCEDEPVASLVPHLAL
DVGGYSLVDEEVSFAFTNYFTWTINSSSLLLDWSSPTTLKIFNNETIFPTEYNVVPLEQTNANEEWVVYVIEDLTGFGIW
HPIHLHGHDFFVVAQESDVFNPDKSPAKFNLINPPRRDVAALPGNGYLAIAFKLDNPGSWLLHCHIAWHASEGLAMQFVE
SQSSIAVGMSDTAIFEDTCANWNAYTPTQLFAEDDSGI
>KAF7925358.1 hypothetical protein EAE99_006222 [Botrytis elliptica]
MKYSTVFTALTAFFAQASASAIPAVRSPLTPRQNTTASCANSATSRSCWGEYSIDTNWYDVTPTGVTREYWLSVENSTIT
PDGYTRSAMTFNGTVPGPAIIADWGDNLIIHVTNNLEYNGTSIHWHGIRQLGSLEYDGVPGVTQCPIAPGDTLTYKFQAT
QYGTTWYHSHFSLQYGDGLFGPLIINGPATADYDEDVGAIFLQDWAHESVFEIWDSARVGAPPALENTLMNGTNIFDCSA
STDPNCVGGGKKFELTFVEGTKYRLRLINVGIDSHFEFAIDGHTLTVIANDLVPIVPYTTDTLLIGIGQRYDVIVEANAA
ADNYWIRGNWGTTCSANMEAANATGILRYDSSSTADPTSVGTTPRGTCEDEPVASLVPHLALDVGAYSLVDEEVSFAFTN
YFTWTINSSSLLLDWSSPTTLKIFNNETIFPTEYNVVPLEQTNNEEWVVYVIEDLTGFGIWHPIHLHGHDFYIVAQEADV
FSPEKSPAKFNLVNPPRRDVAALPGNGYLAIAFKLDNPGSWLLHCHIAWHASEGLAMQFVESQSSIAVGMSDTAIFEDTC
ANWNAYTPTQLFAEDDSGI

>KAF7922626.1 hypothetical protein EAE98_008152 [Botrytis deweyae]
MKYSTVFTALTAFFAQASASAIPAVRSPLTPRQNTTASCANSATSRSCWGEYSIDTNWYDVTPTGVTREYWLSVENSTIT
PDGYTRSAMTFNGTVPGPAIIADWGDNLIIHVTNNLEYNGTSIHWHGIRQLGSLEYDGVPGVTQCPIAPGDTLTYKFQAT
QYGTTWYHSHFSLQYGDGLFGPLIINGPATADYDEDVGAIFLQDWAHESVFEIWDSARVGAPPALENTLMNGTNIFDCSA
STDPNCVGGGKKFELTFVEGTKYRLRLINVGIDSHFEFAIDGHTLTVIANDLVPIVPYTTDTLLIGIGQRYDVIVEANAA
ADNYWIRGNWGTTCSANMEAANATGILRYDSSSTADPTSVGTTPRGTCEDEPVASLVPHLALDVGAYSLVDEEVSFAFTN
YFTWTINSSSLLLDWSSPTTLKIFNNETIFPTEYNVVPLEQTNNDEWVVYVIEDLTGFGIWHPIHLHGHDFYIVAQEADV
FSPEKSPAKFNLVNPPRRDVAALPGNGYLAIAFKLDNPGSWLLHCHIAWHASEGLAMQFVESQSSIAVGMSDTAIFEDTC
ANWNAYTPTQLFAEDDSGI
>THV49336.1 hypothetical protein BGAL_0200g00140 [Botrytis galanthina]
MKYSTVFTALTAFFAQASASAIPAVRSLLTPRQNTTASCANSATSRSCWGEYSIDTNWYDVTPTGVTREYWLSVENSTIT
PDGYTRSAMTFNGTVPGPAIIADWGDNLIIHVTNNLEYNGTSIHWHGIRQLGSLEYDGVPGVTQCPIAPGDTLTYKFQAT
QYGTTWYHSHFSLQYADGLFGPLIINGPATADYDEDVGAIFLQDWAHESVFEIWDSAKLGAPPALENTLMNGTNIFDCSA
STDPNCVGGGKKFELTFVEGTKYRLRLINVGIDSHFEFAIDGHTLTVIANDLVPIVPYTTDTLLIGIGQRYDVIVEANAA
ADNYWIRGNWGTTCSGNLEAANATGILRYDSSSTADPTSVGTTPRGTCGDEPVASLVPHLALDVGGYSLVDEEVSFAFTN
YFTWTINSSSLLLDWSSPTTLKIFNNETIFPTEYNVVPLEQTNANEEWVVYVIEDLTGFGIWHPIHLHGHDFFVVAQESD
VFNPEKSPAKFNLINPPRRDVAALPGNGYLAIAFKLDNPGSWLLHCHIAWHASEGLAMQFVESQSSIAVGMSDTAIFENT
CANWNAYTPTQLFAEDDSGI
>AYM55298.1 laccase2, partial [Botrytis cinerea]
AIPAVRSPLAPRQSTTASCANSATSRSCWGEYSIDTNWYDVTPNTGVTREYWLSVENSTITPDGYTRSAMTFNGTVPGPA
ITADWGDNLIIHVTNNLQHNGTSIHWHGIRQLGSLEYDGVPGVTQCPIAPGDTLTYKFQATQYGTTWYHSHFSLQYADGL
FGPLIINGPATADYDEDVGAIFLQDWAHKSVFEIWDSARQGAPPALENTLMNGTNIYDCSASTDANCVGGGKKFELTFVE
GTKYRLRLINVGIDSHFEFAIDNHTLTVIANDLVPIVPYTTDTLLIGIGQRYDVIVEANAAADNYWIRGNWGTTCSSNSE
AANATGILRYDSSSTVDPTSVGVTPRGTCADEPVASLVPHLALDVGGYSLVDEQVSFAFTNYFTWTINSSSLLLDWSSPT
TLKIFNNETIFPTDYNVVALNQTDANEEWVVYVIEDLTGFGIWHPIHLHGHDFYVVAQETDVFSATKSPANFNLVNPPRR
DVAALPGNGYLAIAFKLDNPGSWLLHCHIAWHASEGLAMQFVESQSSIAIGMSDTDIFEDT
>TGO17305.1 hypothetical protein BTUL_0019g00960 [Botrytis tulipae]
MKYSTIFTALTAFFAQASASAIPAVRSPLTPRQNTTASCANSATSRSCWGEYSIDTNWYNVTPTGVTREYWLSVENSTIT
PDGYTRSAMTFNGTVPGPAIIADWGDNLIIHVTNNLEYNGTSIHWHGIRQLGSLEYDGVPGVTQCPIAPGDTLTYKFQAT
QYGTTWYHSHFSLQYGDGLFGPLIINGPATADYDEDAGVIFLQDWAHESVFEIWDTARLGAAPALENTLMNGTNTFDCSA
STDSNCVGGGKKFELTFVEGTKYRLRLINVGIEGHFEFAIDGHTLTVIANDLVPIVPYTTETLLIGIGQRYDVIVEANAA
ADNYWIRGNWGVTCSSNPEAANATGILRYDSSSTADPTSVGTTPRGTCEDEPVASLVPHLALDVGGYSLVDEQLSFAFTN
YFKWTINSSSLLLDWSSPTTLKIFNNETIFPTEYNVVPLEQTNANEEWVVYVIEDLTSLGVWHPIHLHGHDFFIVAQEAD
MFDPDESPAKFNLVNPPRRDVAALPANGYLAIAFKLDNPGSWLLHCHIAWHASEGLALQFVESQSSIAVGMSDTAIFENT
CANWNAYTPTQLFAEDDSGI
>KAF7909066.1 hypothetical protein EAE99_011547 [Botrytis elliptica]
MSFTLLLCIAILIPACLPSALPSKVSSNNKRISTPTCLNAASSRNCWNGNYSIDTNSEEAWPNTGVVVAYTLTITNQTMS
PDGTPRWMLVVNGTYPGPTITANWGDTLEITVVNQIITDGVSIHWHGIVQKNTNTMDGVNGITECPIPPGSSKVYKFLAT
QHGTSWYHSHHAAQYGDGVLVLGSIVINGPASLNYDYDLGPLPITDFYYKSTYEEGLLSVSKGPPKADNGLINGTNRNPH
KTAGVYNQVSGLVPGAKYRLRIINTSIDNHFQVTLDSHSFQVIQTDFVPIQPYTTNTIFIAIGQRYDVIITADQTPSNYW
FRAIVPKPPTQKGFGCGQNANNGSINAIFSYQGVELSEPNSKSFLIPQSCEDETQLVSWNEKSVPENEFVWPTAQELIVT
GPGSNSLPKAPAPYVWSINDTYMETEWDKPTLQYVAQGNKNYNVHQSIIELPDANVWTFWIIKNAAAIPHPIHLHGHDF
>XP_037197881.1 putative multicopper oxidase protein [Botrytis fragariae]
MRLHILLALCLSFCQIQAIPLFGWSFNPIATLQADLLKIINQVECLASTKVSIIPSILTSGNTNAVTSNKCSTTSGKTTS
AAAVQTSLSSLQSVASSKWSSLQSLLSTTTSLPPILTSSLTVTTAKPTSTSSAKSTSTTANTVSSTAQSSSITSKTSPVS
SSTASLTSKTSSSAVASGTPCAGNTAADRSKWCNYSTSTDYYNEVPNTGVTREYWFNVQDGVASPDGFSRYVQTINGSIP
GPTIIADWGDNIVVHVTNSLSTNGSTIHFHGIHQKNTNQNDGVPSVTQCPIAYGDTYTYRWRATQYGSSWYHSHVGLQAW
EGVAGGIIINGPATANYDEDKGTLMLSDWGHETVDELYEKVQTIGPQSMTTGLINGTNVFGADGASNQTGSRFSTSVESG
KSYRFRLVNSAIDTQFKFSVDSHTMTVMAMDFVPIVPYQTEILNIAIGQRYDVVITANQASVASDFFIRAIPQSSCSEND
NSDNIRGVLHYGSSTGLPTTTGYTFTDECVDEPVSSLVPYLPKTVSAASKSPEEAVTVAQNSQKLFKWYLNNSTFLSEWE
DPTLLMVQNNITTFGTPDNLVAVPNANEWIYLVIHSALPVPHPIHLHGHDFFVVSQQATTFDATTAISTYNLNNPHEETW
PHCQEVDISMGFAMQFLERASEIPKLLDTTQLQDTCTAWNGHAVIQEDSGV
>TGO35656.1 hypothetical protein BHYA_0151g00280 [Botrytis hyacinthi]
MYFTLLLCVAILIPSCLPSAIPSQVLSNNKRTSTSTCLNAASSRNCWNGNYSIDTDPEEAWPNTGVVVAYTLTITNQTMS
PDGTPRWMLVVNGTYPGPTITASELFPMWPPFPSAKLAHLFTDWGDTVQITVVNQIITDGVSIHWHGIVQKDTNTMDGVN
GITECPIPPGSSKVYKFLATQHATSWYHSHHAAQYGDGVLGSIVINGPASLNYDYDLGPLPITDFYYKSAYEEGLLSVSK
GPPKADNGLINGTNQNPHKIAGVYNQVSGLVPGAKYRLRIINTSIDNHFQVTLDSHSFQVIQTDFVPIQPYTTNTIFMAI
GQRYDIIITADQTPSNYWFRAIVPKPPTQKGFSCGQNANNGSINAIFSYQGVESSEPNSKSFLIPQSCEDETQQVSWHEK
SVPENEFVWPTAQELIVTGPGSNSLPKSPAPYVWSINDTYMEMEWDKPTLQYVAQGNKNYNVHQSIIELPDANVWDILDH
QKCCRDTTPYSSSWSRLLHFRTGEWCQFHRQI
>KAF7933110.1 hypothetical protein EAE97_008877 [Botrytis byssoidea]

MLFADFITQATTLLVASLSQLQTNGLSLWGTLDVPQLPLYNSTNAPQTSLQNTYGVIPNTGRTVYYDWTITRGYIAPDGV
NKSVILINGQFPGPLLDANWGDMIQVTVRNHIKDEGTTIHWHGLLQKGTPWMDGVPGISQCPISPGSQFTYRFRADQYGT
SWYHSHYSGQYGAGLFGPLVIYGPQNVHYDIDIGPVMLHDWYHADYYQVVERLFSVPANPLAANNNLINGKMDFNCSAIT
VNASCTANAGLSKFHFHPGKTHRLRLINPGTEGNQKFSIDGHLLTVIAVDFVPVVPYDTNVVTVGVGQRTDVIVKAVGKV
GSSYWMRSNFSQLCASSPSLQPNALAVVLYDGADPNSRPKSTAFPYNETNCLNDPLNTTTPYIKSTPPVTPQVTQTIDIN
YLINGSGMFLFTFGGSSFRADYNQPVLLTAATATTSTSNSGIYTNSTSLYPTTMNVHDFGTPATVRLIITNSVPVSHPMH
LHGHNFWILAEGVGTWDGTITNPSNPIRRDTFMMAAALRSGPKAYTVIEYVADNPGVWPFHCHVAGHVSTGLLINVLEQP
DVLANNSQALNSISPTCESWQKWTKSHLVDEIDSGE
>XP_024550974.1 Bclcc3 [Botrytis cinerea B05.10]
MVSHVFGRILCLVGSYLRFIDLSDYCFSEQSSIPADNTPTSRDCWGAYDIHTDYSNEWPTTNVVREYDLTASNMWIAPDG
VEVLGMVFNSQFPAPTIEADWGDTLRITVHNNLMNLNGTAVHWHGLRQLNSNWQDGVPGVSQCPITPFQSYTYEFQVTQY
GVSWYHSHFALQYPAGLYGPLKINGPTSQNYDIDIGPLLISDWFHQSPFSLFYEELCCNTPIPNSHLLQGQGVYYDETTG
LRSGSFYSLGVARGKKYKISLVNAGTSTQFTFWIDGHTFTVVGTDFVPIEGYETDTLNIAVGQRYDIIIKANAGCSHGTN
FWIHARDCNSPTQISTLGILRYDPTSTELPPDSHDNQLNFGCLEPPAKFLVPVVSKKVGKPINGLTPNQYLNVSLQPYPK
LDPDTHLLKWSLKNYPMYLNWSVPSLKLIQDNSDTTGVAFPQDYVPIFLDYPDNDWLYFLIEGMFNTTDPTIEVSNSSHP
IHLHGHDFSILAQSHTPFDISTFSPNLDNPPRRDTAMLPQNGYLVIGFEMNNPGAWLLHCHIAWHASSGMAVQFVESSGQ
LQSMIRKAGIMSSLEDQCDAWTSWYYDYNVPAEHTQDAESGV
>TGO22870.1 hypothetical protein BPAE_0152g00250 [Botrytis paeoniae]
MLFADFITQATTLLVASLSQLETNGLSLWGTLDVPQLPLYNSTNAPQTSLQNTYGAIPNTGRTVYYDWTISRGYIAPDGV
NKSVILINGQFPGPLLDANWGDMIQVTVRNYIKDEGTTIHWHGLLQSGTPWMDGVPGISQCPISPGSQFTYRFRADQYGT
SWYHSHYSGQYGAGLFGPLVIYGPQNVHYDVDIGPVMLHDWYHADYYQVVERLFSVPANPLAANNNLINGKMDFNCSAIT
VNASCTANAGLSKFQFYPGKTHRLRLINPGTEGNQKFSIDGHVLTVIAVDFVPVVPYDTNVVTIGVGQRTDVIVKAIGKV
GSSYWMRSNFSQLCASSPSLQPNALAVVLYDGANPNSRPNSTPFPYNETNCLNDPLNTTTPYVKSSPPVTPQVTQTIDIN
YVINGSGMFLFTFGGSSFRADYNQPVLLTAATATTSTSNSSIYTNSTSLYPTTMNVHDFGTPASVRLIITNSVPVSHPMH
LHGHNFWILAEGVGTWDGTITNPSNPIRRDTFMMAAALRTGSKAYTVIEYVADNPGVWPFHCHVSGHVSTGLLINVLEQP
NMLANNSQALKSISPTCESWQKWTKSHLVDEIDSGE
>THV45850.1 hypothetical protein BGAL_0444g00080 [Botrytis galanthina]
MLFADFITQATTLLVASLSQLQTNGLSLWGTLDVPQLPLYNSSNAPQTSLQNTYGAIPNTGRTVYCDWTITRGYIAPDGV
NKSVILINGQFPGPLLDANWGDMIQVTVRNHIRDEGTTIHWHGLLQNGTPWMDGVPGISQCPISPGSQFTYRFRADQYGT
SWYHSHYSGQYGAGLFGPLVIYGPQNVHYDIDIGPVMLHDWYHADYYQVVERLFSVPANPLAANNNLINGKMDFNCSAIT
VNASCTANAGLSKFHFHPGKTHRLRLINPGTEGNQKFSIDGHVLTVIAVDFVPVVPYDTNVVTVGVGQRTDVIVKAIGKV
GSSYWMRSNFSQLCASSASLQPNALAVVLYEGADPNSRPKSTPFPYNETNCLNDPLNTTTPYVKSTPPVKPQVTQTIDIN
YVINGSGMFLFTFGGSSFRADYNKPVLLTAATATTSTSNSGTYTNSTSLYPTTMNVHDFGTPASVRLIITNSVPVSHPMH
LHGHNFWILAEGVGTWDGTITNPSNPIRRDTFMMAAALRTGSKAYTVIEYVADNPGVWPFHCHVAGHVSTGLLINVLEQP
DVLANNSQALKSISPTCESWQKWTKSHLVNEIDSGE
>KAF7935780.1 hypothetical protein EAE99_002760 [Botrytis elliptica]
MLFADFITQATTLLVASLSQLQTNGLSLWGTLDAPQLPLYNLTNAPQTSLQNTYGAIPNTGRTVYYDWTITRGYIAPDGV
NKSVILINGQFPGPLLDANWGDMIQVTVRNHIKDEGTTIHWHGLLQNGTPWMDGVPGISQCPISPGSQFTYRFRADQYGT
SWYHSHYSGQYGAGLFGPLVIYGPQNVHYDIDIGPVMLHDWYHADYYQVVERLFSVPANPLAANNNLINGKMDFNCSAIT
VNASCTANAGLSKFHFHPGKTHRLRLINPGTEGNQKFSIDGHVLTVIAVDFVPVVPYDTNVVTVGVGQRTDVIVKAIGKV
GSSYWMRSNFSQLCASSPSLQPNALAVVLYDGANPNSRPKSTPFPYNETNCLNDPLNTTTPYVKSTPPVTPQVTQTIDIN
YVINGSGMFLFTFGGSSFRADYNQPVLLTAATAKTSTSNSGTYINSTSLYPTTMNVQDFGTPASVRLIITNSVPVSHPMH
LHGHNFWILAEGVGTWDGTITNPSNPIRRDTFMMAAALRTGPKAYTVIEYVADNPGVWPFHCHVAGHVSTGLLINVLEQP
GVLANNSQALKSILPTCESWQKWTKSHLVDEIDSGE
>KAF7930322.1 hypothetical protein EAE98_004723 [Botrytis deweyae]
MLFADFITQATTLLVASLSQLQTNGLSLWGTLDAPQLPLYNLTNAPQTSLQNTYGAIPNTGRTVYYDWTITRGYIAPDGV
NKSVILINGQFPGPLLDANWGDMIQVTVRNHIKDEGTTIHWHGLLQNGTPWMDGVPGISQCPISPGSQFTYRFRADQYGT
SWYHSHYSGQYGAGLFGPLVIYGPQNVHYDIDIGPVMLHDWYHADYYQVVERLFSVPANPLAANNNLINGKMDFNCSAIT
VNASCTANAGLSMFHFHPGKTHRLRLINPGTEGNQKFSIDGHVLTVIAVDFVPVVPYDTNVVTVGVGQRTDVIVKAIGKV
GSSYWMRSNFSQLCASSPSLQPNALAVVLYDGANPNSRPKSTPFPYNETNCLNDPLNTTTPYVKSTPPVTPQVTQTIDIN
YVINGSGMFLFTFGGSSFRADYNQPVLLTAATAKTSTSNSGTYTNSTSLYPTTMNVQDFGTPASVRLIITNSVPVSHPMH
LHGHNFWILAEGVGTWDGTITNPSNPIRRDTFMMAAALRTGPKAYTVIEYVADNPGVWPFHCHVAGHVSTGLLINVLEQP
GVLANNSQALKSILPTCESWQKWTKSHLVDEIDSGE
>KAF7881612.1 hypothetical protein EAF02_006300 [Botrytis sinoallii]
MLFADFITQATTLLVASLSQLQTNGLSLWGTLDAPQLPFYNLTNAPQTSLQNTYGAIPNTGRTVYYDWTITRGYIAPDGV
NKSVILINGQFPGPLLDANWGDMIQVTVRNHIKDEGTTIHWHGLLQNGTPWMDGVPGISQCPISPGSQFTYRFRADQYGT
SWYHSHYSGQYGAGLFGPLVIYGPHNVHYDIDIGPVMLHDWYHADYYQVVERLFSVPANPLAANNNLINGKMDFNCSAIT
VNASCTANAGLSKFHFHPGKTHRLRLINPGTEGNQKFSIDGHVLTVIAIDFVPVVPYDTNVVTVGVGQRTDVIVKAIGKV
GSSYWMRSNFSQLCASSPSLQPNALAVVLYDGANPNSRPKSTPFSYNETNCLNDPLNTTTPYVKSTPPVTPQVTQIIDIN
YVINGSGMFLFTFGGSSFRADYNQPVLLTAATATTSTSNSGTYTNSTSLYPTTMNVQDFGTPASVRLIITNSVPVSHPMH
LHGHNFWILAEGVGTWDGTITNPSNPIRRDTFMMAAALRTGSKAYTVIEYVADNPGVWPFHCHVAGHVSTGLLINVLEQP

DVLANNSQALKSISPTCESWQKWTKSHLVDEIDSGE
>KAF7955862.1 hypothetical protein EAE96_004784 [Botrytis aclada]
MLFADFITQATTLLVASLSQLQTNGLSLWGTLDVPQLPLYNSTNAPQTSLQNTYGAIPNTGRTVYHDWTISRGYIAPDGV
NKSVILINGQFPGPLLVANWGDMIQVTVRNHIKDEGTTIHWHGLLQNGTPWMDGVPGISQCPISPGSQFTYRFRADQYGT
SWYHSHYSGQYGAGLFGPLVIYGPQNVHYDIDIGPVMLHDWYHSDYYQVVERLFSVPANPLAANNNLINGKMDFNCSAIT
VNASCTANAGLSKFHFHPGKTHRLRLINPGTEGNQKFSIDGHVLTVIAVDFVPVVPYDTNVVTVGVGQRTDVVVKATGKV
GSSYWMRSNFSQLCASSPSLQPNALAVVLYDGANPNSRPKSTPFPYNETNCLNDPLNTTTPYVKSTPPVTPQVTQTIDIN
YVVNGSGMFLFTFGGSSFRADYNQPVLLTAVTATTSTSNSGTYTNSTSLYPTTMNVHDFGTPATVRLIITNSVPVSHPMH
LHGHNFWILAEGVGTWDGTITNPSNPIRRDTFMMAAALRTGSKAYTVIEYIADNPGVWPFHCHVSGHVSTGLLINVLEQP
DVLANNSQALKSISPTCESWQKWTKSHLVDEIDSGE
>TGO73487.1 hypothetical protein BELL_0357g00060 [Botrytis elliptica]
MLFADFITQATTLLVASLSQLQTNGLSLWGTLDAPQLPLYNLTNAPQTSLQNTYGAIPNTGRTVYYDWTITRGYIAPDGV
NKSVILINGQFPGPLLDANWGDMIQVTVRNHIKDEGTTIHWHGLLQNGTPWMDGVPGISQCPISPGSQFTYRFRVYQYGT
SWYHSHYSGQYGAGLFGPLVIYGPQNVHYDIDIGPVMLHDWYHADYYQVVERLFSVPANPLAANNNLINGKMDFNCSAIT
VNASCTANAGLSKFHFHPGKTHRLRLINPGTEGNQKFSIDGHVLTVIAVDFVPVVPYDTNVVTVGVGQRTDVIVKAIGKV
GSSYWMRSNFSQLCASSPSLQPNALAVVLYDGANPNSRPKSTPFPYNETNCLNDPLNTTTPYVKSTPPVTPQVTQTIDIN
YVINGSGMFLFTFGGSSFRADYNQPVLLTAATAKTSTSNSGTYINSTSLYPTTMNVQDFGTPASVRLIITNSVPVSHPMH
LHGHNFWILAEGVGTWDGTITNPSNPIRRDTFMMAAALRTGPKAYTVIEYVADNPGVWPFHCHVAGHVSTGLLINVLEQP
GVLANNSQALKSILPTCESWQKWTKSHLVDEIDSGE
>KAF7929387.1 hypothetical protein EAE98_005305 [Botrytis deweyae]
MTWFGLFAGIFTAGAGIINTLSQSATNGNSVWGTLQAPTFPPFLTDNPLPNGYPWGSLTASGNNPYTSAPNTGVIRSYDF
TVTRGTIAPDGYSKDVILVNGQFPGPAIEANWGDTFVITVHNQITGPEEGTAFHWHGLLQKGTQYMDGVPAVTQCPIAPG
ASFTYTFKADLYGSSWYHSHYSAQYAGGLVGPMIIHGPANAPYDIDLGPVFLTDYYHKDYFSIVKSVVESNGDAKPFSDN
NLINGKMNFDCSTKAAGDNTACSDNAGISRFKFTTGKTHRLRLINAGAEGMQRFSIDGHKMVVIANDFVPVKPYTTNVVT
LAVGQRTDVLVTANVGTSDSSFWMRSNISTTCSLSNQPNALAAIYYDGADTNSTPASTAWDVPDPGTCTNDELDTTEPYY
SLAAETSSTTQNLDIGSYVNDTGSFLWTLGGTSFRANYNNPILSLANQGNFTYPDEWNVRNFGSNTTIRVVVNNPTPASH
PMHLHGHNMQILHEGDGDWDGVTITRQANPQRRDVQLVRANGHLVWQITTDNPGVWPFHCHIAWHVSGGLYANILERPDD
IKNDAIVNSVQQTCTQWDAYSSTAVVDEIDSGL
>XP_037196983.1 putative extracellular dihydrogeodin oxidase laccase protein [Botrytis fragariae]
MAWFGLFAGIFTAGAGIINTLSQTATNGNSVWGTLQAPTFPPFLTDNPLPNGYPWGSLTASGNNPYTSAPHTGVIRSYNF
TVTRGTIAPDGYSKDVILVNGQFPGPAIEANWGDTFVITVHNQITGPEEGTAFHWHGLLQKGTQYMDGVPAVTQCPIAPG
ASFTYTFKADLYGSSWYHSHYSAQYAGGLVGPMIIHGPNNAPYDIDLGPVFLTDYYHKDYFSIVKSVVESNGDSKPLSDN
NLINGKMNFDCSTKAAGDNTACSDNAGISRFKFTTGKTHRLRLINAGAEGMQRFSIDGHKMVVIANDFVPVKPYTTNVVT
LAVGQRTDVLVTANVGTSDSSFWMRSNISTTCSLSNQPNALAAIYYDGADTNSTPASTAWDVPDPGTCTNDELDTTEPYY
SLAAETPSTTQNLDINFYVNDTGSFLWTLGGTSFRANYNNPILSLANQGNMTYPDEWNVRNFGSNTTIRVIVNNPTPASH
PMHLHGHNMQILHEGDGDWDGVTITRQSNPQRRDVQLVRANGHLVWQITTDNPGVWPFHCHIAWHVSGGLYANILERPDD
IKNDAIATSVEQTCTQWDAYSSTAVVDEIDSGL
>TGO17900.1 hypothetical protein BTUL_0014g00230 [Botrytis tulipae]
MLFADFITQASTLLVASLSQLQTNGLSLWGTLDVPQLPLYNLTNAPQTSLQNTYGAIPNTGRTVYYDWTITRGYIAPDGV
NKSVILINGQFPGPLLDANWGDMIQVTVRNHIKDEGTTIHWHGLLQKGTPWMDGVPGISQCPISPDSQFTYLFRADQYGT
SWYHSHYSGQYGAGLFGPLVIYGPQNVQYDIDIGPVMLHDWYHADYYQVVERLFSVPANPIAANNNLINGKMDFNCSAIT
VNASCTANAGLSKFHFHPGKTHRLRLINPGTEGNQKFSIDGHVLTVIAVDFVPVVPYDTKVVTVGVGQRTDVIVKAIGKV
GSSYWMRSNFSQLCASSPSLQPNALAVVLYDGADPNSRPKSTPFPYNETNCLNDPLNTTTPYVKSTPPVTPQVTQTIDIN
YLINGSGMFLFTFGGSSFRADYNQPVLLTAATATTSTSNSGTYTNSTSLYPTTMNVHDFGTPATVRLIITNSVPVSHPMH
LHGHNFWILAEGVGTWDGTITNPSNPIRRDTFMMAAAIRSGPKAYTVIEYVADNPGVWPFHCHVAGHVSTGLLINVLEQP
DVLANNSQALKSISPTCESWQKWTKSHLVDEIDSGE
>THV54155.1 hypothetical protein BGAL_0033g00270 [Botrytis galanthina]
MTWFGLFAGIFTAGAGIINTMSQTATNGNSVWGTLQAPAFPPFLTDNPLPNGYPWGSLTASGNNPYTSAPHTGVVRSYDF
TVTRGTISPDGYSKDVILVNGQFPGPAIEANWGDTFEITVHNQITGPEEGTAFHWHGLLQKGTQYMDGVPAVTQCPIAPG
ASFTYTFKADLYGSSWYHSHYSAQYAGGLIGPMIIHGPKNAPYDIDLGPVFLTDYYHKDYFSIVKSVVESNGDAKPFSDN
NLINGKMNFDCSTKAAGDNTACSDNAGISRFKFTTGKTHRLRLINAGAEGMQRFSIDGHKMVVIANDFVPVKPYTTNVVT
LAVGQRTDVLVTANVGTSDSSFWMRSNISTTCSLSNQPNALAAVYYDGADTNSTPASTAWDVPDPGTCTNDELDTTEPYY
SLAAEAASTTQNLDINSYVNETGTFLWTLGGTSFRANYNNPVLSLANQGNFTYPDEWNVRNFGSNTTIRIVVNNPTPASH
PMHLHGHNMQILHEGAGDWDGVTVTRQSNPQRRDVQLVRANGHLVWQITTDNPGVWPFHCHIAWHVSGGLYANILERPDD
IKNDAIATSALQTCTQWNAYSSTAVVDEIDSGL
>KAF7922903.1 hypothetical protein EAE99_007095 [Botrytis elliptica]
MTWFGLFAGIFTAGAGIINTLSQSATNGNSVWGTLQAPTFPPFLTDNPLPNGYPWGSLTASGNNPYTSAPNTGVIRSYDF
TVTRGTIAPDGYSKDVILVNGQFPGPAIEANWGDTFVITVHNQITGPEEGTAFHWHGLLQKGTQYMDGVPAVTQCPIAPG
ASFTYTFKADLYGSSWYHSHYSAQYAGGLVGPMIIHGPANAPYDIDLGPVFLTDYYHKDYFSIVKSVVESNGDAKPFSDN
NLINGKMNFDCSTKAAGDNTACSDNAGISRFKFTTGKTHRLRLINAGAEGMQRFSIDGHKMVVIANDFVPVKPYTTNVVT
LAVGQRTDVLVTANVGTSDSSFWMRSNISTTCSLSNQPNALAAIYYDGADTNSTPASTAWDVPDPGTCTNDELDTTEPYY

SLAAETSSTTQNLDIGSYVNDTGSFLWTLGGTSFRANYNNPILSLANQGNFTYPDEWNVRNFGSNTTIRVVVNNPTPASH
PMHLHGHNMQILHEGDGDWDGVTITRQANPQRRDVQLVRANGHLVWQITTDNPGVWPFHCHIAWHVSGGLYANILERPDD
IKNDAIVNSVQQICTQWDAYSSTAVVDEIDSGL
>KAF7893241.1 hypothetical protein EAF02_000779 [Botrytis sinoallii]
MSWFGLFAGIFTAGAGIINTLSQSATNGNSVWGTLQAPTFPPFLTDNPLPNGYPWGSLTASGNNPYTSAPHTGVIRSYDF
TVTRGTIAPDGYSKDVILVNGQFPGPAIEANWGDTFVITVHNQITGPEEGTAFHWHGLLQKGTQYMDGVPAVTQCPIAPG
ASFTYTFKADLYGSSWYHSHYSAQYAGGLVGPMIIHGPANAPYDIDLGPVFLTDYYHKDYFSIVKSVVESNGDAKPFSDN
NLINGKMNFDCSTKAAGDNTACSDNAGISRFKFTTGKTHRLRLINAGAEGMQRFSIDGHKMVVIANDFVPVKPYTTNVVT
LAVGQRTDVLVTANVGTSDSSFWMRSNISTTCSLSNQPNALAAIYYDGADTNSTPASTAWDVPDPGTCTNDELDTTEPYY
SLAAETSSTTQNLDIGSYVNDTGSFLWTLGGTSFRANYNNPILSLANQGNFTYPDEWNVRNFGSNTTIRVVVNNPTPASH
PMHLHGHNMQILHEGDGDWDGVTITRQANPQRRDVQLVRANGHLVWQITTDNPGVWPFHCHIAWHVSGGLYANILERPDD
IKNDAIVNSVQQTCTQWDAYSSTAVVDEIDSGL
>TGO25281.1 hypothetical protein BPAE_0083g00130 [Botrytis paeoniae]
MTWFGLFAGIFTAGAGIMNTLSQTATNGNSVWGTLQAPTYPPFLTDNPLPNGYPWGSLTAGGNNPYTSAPHTGVIRSYDF
TVTRGTIAPDGYSKDVILVNGQFPGPAIEANWGDTFVITVHNQITGPEEGTAFHWHGLLQKGTQYMDGVPAVTQCPIAPG
ASFTYTFKADLYGSSWYHSHYSAQYAGGLVGPMIIHGPENAPYDIDLGPVFLTDYYHKEYFSIVKSVVESNGDSKPFSDN
NLINGKMNFDCSTKAAGDNTACSDNAGISRFKFTTGKTHRLRLINAGAEGMQRFSIDGHKMVVIANDFVPVKPYTTNVVT
LAVGQRTDVLVTANVGTSDSSFWMRSNISTTCSLSNQPNALAAIYYDGADTNSTPASTAWDVPDPGTCTNDELDTTEPYY
SLAAETPSTTQNLDINFYVNETGSFLWTLGGTSFRANYNNPILSLANQGNFTYPDEWNVRNFGSNTTIRVIVNNPTPASH
PMHLHGHNMQILHEGDGDWDGVTITRQSNPQRRDVQLVRANGHLVWQITTDNPGVWPFHCHIAWHVSGGLYANILERPDD
IKNDAIVTSVEQTCTQWDAYSSTAVVDEIDSGL
>TGO13734.1 hypothetical protein BTUL_0064g00350 [Botrytis tulipae]
MTWFDLFAGIFTAGAGIINTMSQTATNGNSVWGTLQAPTFPPFLTDNPLPSGFPWGSLTAGGNNPYTSAPHTGVIRSYDF
TVTRGTISPDGYSKDVILVNGQFPGPAIEANWGDTFVITVHNQITGPEEGTAFHWHGLLQKETQYMDGVPAVTQCPIAPG
ASFTYTFKADLYGSSWYHSHYSAQYAGGLMGPMIIHGPKNAPYDIDLGPVFLTDYYHKDYFSIVESVVESNGNAKPFSDN
NLINGKMNFDCSTKAAGDNTACSDNAGISRFKFTTGKTHRLRLINAGAEGIQRFSIDGHKMVVIANDFVPVKPYTTNVVT
LAVGQRTDVLVTANVGTSDSSFWMRSNISTTCSLSNQPNALAAIYYDGADTNSTPASTAWDIPDLGICTNDDLDTTEPYY
TLAAETPSTTQNLDIGSYVNDTGTFLWTLGGTSFRANYNNPILSLANEGNFTYPDEWNVRNFGSNTTIRIVVNNPTPASH
PMHLHGHNMQILHEGDGDWDGVTVTRQANPQRRDVQLVRANGHLVWQITTDNPGVWPFHCHIAWHVSGGLYANILERPDD
IKNDAIATSALQTCTQWNAYSSTAVVDEIDSGL
>TGO33302.1 hypothetical protein BHYA_0254g00180 [Botrytis hyacinthi]
MTWFGLFAGIFTAGAGIINTMSQTATNGNSYWGTLQAPTFPPYLTDNPLPNGYPWGSLTASGNNPYTSAPQTGVIRSYDF
TVTRGTISPDGYSKDVILVNGQFPGPAIEANWGDTFVITVHNQITGPEEGTAFHWHGLLQKGTQYMDGVPAVTQCPIAPG
ASFTYTFKADLYGSSWYHSHYSAQYAGGLVGPMIIHGPKNAPYDIDLGPVFLTDYYHKDYFSIVKSVVESNGDAKPFSDN
NLINGKMNFDCSTKAAGDNTACSDNAGISRFKFTTGKTHRLRLINAGAEGMQRFSIDGHKMVVIANDFVPVKPYTTNVVT
LAVGQRTDVLVTADVGTSDSAFWMRSNISTTCSLSNQPNALAAIYYDGADTNSTPASTAWDVPDPGTCTNDELDTTEPYY
SLAAETPSTTQNLDINSYVNETGTFLWTLGGTSFRANYNNPILSLANEGNFTYPDEWNVRNFGSNTTIRIVVNNPTPASH
PMHLHGHNMQILHEGAGDWDGVTVTRQANPQRRDVQLVRANGHLVWQITTDNPGVWPFHCHIAWHVSGGLYANILERPDD
IKNDAIATSALQTCTQWNAYTSTAVVDEIDSGL
>KAF7894537.1 hypothetical protein EAF01_009988 [Botrytis porri]
MTWFGLFAGMMAAGAGMISTLSQTATNGNSVWGTFEAPTFPHFLTDNPLPNGFPWGSMTAGGNDPYTSAPHTGVVRSYDF
NVTRGTIAPDGYSKDVILVNGQFPGPAIEANWGDTFVIIVHNQITGPEEGTAFHWHGLLQKGTQYMDGVPAVTQCPIAPG
ASFTYTFKADLYGTSWYHSHYSAQYAGGLVGPMIIHGPQNAPYDIDLGPVFLTDYYHKDYFSIVKSVVESNGDSKPSSDN
NLINGKMNFDCSTKAEGDNTACTDNAGISRFKFTTGKTHRLRLINAGAEGMQRFSIDGHKMVVIANDFVPVKPYTTNVVT
LAVGQRTDVLVTANAESSDSAFWMRSNISTTCSLTKQPIALAAIYYDGADTDSTPASTAWDVPDPGTCTNDELDVTEPYY
SLAAETPSTTSNLDINFYVNETGSFLWTLGGTSFRANYNNPILNLANQGNFTYPDEWNVRNFGSNTTIRVIVNNPTPASH
PMHLHGHNMQILHEGDGDWDGVTITRQSNPQRRDVQLVRANGHLVWQITTDNPGVWPFHCHIAWHVSGGLYANILERPDD
IKNDAILTSVEQTCTQWGAYSSTAVVDEIDSGL
>XP_024546439.1 Bclcc5 [Botrytis cinerea B05.10]
MTFFGLFAGIFAAGAGFIGTLSQTATNGNSVWGTLQAPTYPPFLSNNPLPYGYPWGSMTAGGNNPYTSAPHTGAVRSYDF
TVTRGQISPDGYLRDVILINGQYPGPAIEANWGDTFVITVHNNITGPEEGTSFHWHGLLQKGTQYMDGVPAVTQCPIAPG
ASFTYNFKADLYGTSWYHSHYSAQYAGGLVGPMIIHGPKNAPYDIDLGPVFLSDYYHKDYFSIVKSVVESNGDGKPFSDN
NLINGKMNFDCSTKAEGDNTECSDNAGLSRFKFTTGKTHRLRLINAGAEGMQRFSIDGHKMVVIANDFVPVKPYTTNVVT
LAVGQRTDVLVTANAGSSDSSFWMRSNISTICSLANQPNALAAIYYDSADTNSTPASTAWDVPDPGTCTNDELDTTEPYY
SLSAETPSTTQNLDINSYVNETGTFLWTLGGTSFRANYNSPVLSLANQGNFTYPDEWNVRNFGSNTTIRIVVNNPTPASH
PMHLHGHNMQILHEGDGDWDGVTITRQSNPQRRDVQLVRANGHLVWQITTDNPGVWPFHCHIAWHVSGGLYANILERPDD
IKNDDIFTSTEKSCTAWNAYSSTAVVDEIDSGL
>KAF7959086.1 hypothetical protein EAE96_002603 [Botrytis aclada]
MTLFGLFAGIFTAGAGIINTLSQNATNGNSVWGTLDAPTFPPFLTDNSLPHGFPWGSLTAGGNNPYTSAPHTGVVRSYDF
TVTRGTIAPDGYSKDVILVNGQFPGPAIEANWGDTFVITVHNQITGPEEGTAFHWHGLLQKGTQYMDGVPAVTQCPIAPG
ASFTYTFKADLYGSSWYHSHYSAQYAGGLVGPMIIHGPKNAPYDIDLGPVFLTDYYHKDYFSIVKSVVESNGDSKPISDN

NLINGKMNFDCSTKAAGDNTACSDNAGISRFEFTTGKTHRLRLINAGAEGMQRFSIDGHKMVVISNDFVPVKPYTTNVVT
LAVGQRTDVLVTANVGSSDSSFWMRSNISTTCSLSNQPNALAAIYYDAADKDSTPTSTAWDVPDPGTCTNDELDTTEPYY
SLAAETPSTTQNLDINFYVNDTENFLWTLGGTSFRANYNNPILSLANQGNFTYPDEWNVRNFGSNTTIRVIVNNPTPASH
PMHLHGHNMQILHEGDGDWDGVTITRQANPQRRDVQLVRANGHLVWQITTDNPGVWPFHCHIAWHVSGGLYANILERPDD
IKNDAILNSMEQTCTQWDAYSSRAVVDEIDSGL
>KAF7949419.1 hypothetical protein EAE97_002928 [Botrytis byssoidea]
MTWFGLFAGIFTAGAGIINTMSQTATNGNSAWGTLQAPTFPPFLTDNPLPNGYPWGSLTAGGNNPYTSAPHTGVVRSYDF
TVTRGTISPDGYSKDVILVNGQFPGPAIEANWGDTFVITVHNQITGPEEGTAFHWHGLLQKETQYMDGVPAVTQCPIAPG
ASFTYTFKADLYGSSWYHSHYSAQYAGGLMGPMIIHGPKNAPYDIDLGPVFLTDYYHKDYFSIVKSVVESNGDAKPFSDN
NLINGKMNFDCSTKAAGDNTACSDNAGISRFKFTTGKTHRLRLINAGAEGIQRFSIDGHKMVVIANDFVPVKPYTTNVVT
LAVGQRTDVLVTANVGTSDSSFWMRSNISTTCSLSNQPNALAAIYYDGADTNSTPASTSWDIPDLAICTNDDLDTTEPYY
TLAAETPSTTQNLDIGSYVNDTGTFLWTLGGTSFRANYNNPILSLANEGNLTYPDEWNVRNFGSNTTIRIVVNNPTPASH
PMHLHGHNMQILHEGDGDWDGVTVTRQANPQRRDVQLVRANGHLVWQITTDNPGVWPFHCHIAWHVSGGLYANILERPDD
IKNDAIATSALQTCTQWNAYSSTAVVDEIDSGL
>QFF92556.1 extracellular dihydrogeodin oxidase/laccase-like protein isoform 2 [Botrytis fabae]
MTLFGLFAGIFTAGAGIINTLSQNATNGNSVWGTLDAPTFPPFLTDNSLPHGFPWGSLTAGGNNPYTSAPHTGVVRSYDF
TVTRGTISPDGYSKDVILVNGQFPGPAIEANWGDTFVITVHNQITGPEEGTAFHWLGLLQKETQYMDGVPAVTQCPIAPG
ASFTYTFKADLYGSSWYHSHYSAQYAGGLMGPMIIHGPKNAPYDIDLGPVFLTDYYHKDYFSIVKSVVESNGDSKPISDN
NLINGKMNFDCSTKAAGDNTACSDNAGISRFEFTTGKTHRLRLINAGAEGMQRFSIDGHKMVVISNDFVPVKPYTTNVVT
LAVGQRTDVLVTANVGSSDSSFWMRSNISTTCSLSNQPNALAAIYYDAADKDSTPTSTAWDVPDPGTCTNDELDTTEPYY
SLAAETPSTTQNLDINFYVNDTENFLWTLGGTSFRANYNNPILSLANQGNFTYPDEWNVRNFGSNTTIRIVVNNPTPASH
PMHLHGHNMQILHEGDGDWDGVTVTRQANPQRRDVQLVRANGHLVWQITTDNPGVWPFHCHIAWHVSGGLYANILERPDD
IKNDAIATSALQTCTQWNAYSSTAVVDEIDSGL
>QFF92555.1 extracellular dihydrogeodin oxidase/laccase-like protein isoform 1 [Botrytis fabae]
MTLFGLFAGIFTAGAGIINTLSQNATNGNSVWGTLDAPTFPPFLTDNSLPHGFPWGSLTAGGNNPYTSAPHTGVVRSYDF
TVTRGTISPDGYSKDVILVNGQFPGPAIEANWGDTFVITVHNQITGPEEGTAFHWLGLLQKETQYMDGVPAVTQCPIAPG
ASFTYTFKADLYGSSWYHSHYSAQYAGGLMGPMIIHGPKNAPYDIDLGPVFLTDYYHKDYFSIVKSVVESNGDAKPFSDN
NLINGKMNFDCSTKAAGDNTACSDNAGISRFKFTTGKTHRLRLINAGAEGIQRFSIDGHKMVVIANDFVPVKPYTTNVVT
LAVGQRTDVLVTANVGTSDSSFWMRSNISTTCSLSNQPNALAAIYYDGADTNSTPASTSWDIPVLAICTNDDLDTTEPYY
TLAAETPSTTQNLDIGSYVNDTGTFLWTLGGTSFRANYNNPILSLANEGNLTYPDEWNVRNFGSNTTIRIVVNNPTPASH
PMHLHGHNMQILHEGDGDWDGVTVTRQANPQRRDVQLVRANGHLVWQITTDNPGVWPFHCHIAWHVSGGLYANILERPDD
IKNDAIATSALQTCTQWNAYSSTAVVDEIDSGL
>XP_001549796.1 Bclcc9 [Botrytis cinerea B05.10]
MRGLGSLATLLFGSLVSAQNNYSALPVNFLKTSNPVTPLPQGYPWGNRTANNTNPYTGAPHTGVIRKYDFTVKRGQASPD
GYLRDVILVNDVFPGPLIEANWGDTIEVTVHNEIRGPEEGTALHWHGLLQKETQWFDGVPGVQQCPIVPGGTFTYSFLAD
LYGTSWWHSHYSAQYNAGIFGPMIIHGPPTVPYDIDMGPILLNDWYHPDYFSLVEDVMSTDLNRVLVSSDSNLIQGKGFF
DCSTKAAGDNATCNDENYMPPTFKFTAGLKHRLRLINAGTEGMQKFAIDGHNMTVIANDFVPIIPYTTQVVTLGVGQRTD
VIVEGLPTGTTGSFWMRSTIAGCSLAKNPEAKALVYYDLDPNVNSTTTASATFNDSVANVCANDPLNETTPWYPITPDPN
PPTTEQIDVGFGQNASGNWLWTMNNSSFRANFNEPILLLSNQGNNSYPDSPQWNVYNFGDNSSIRIYITNPTAVSHPMHL
HGHNMFVLNEGLGLWDGKTVVRPSNPQRRDVQTLQAGGYMVLQITADNPGVWPLHCHIAFHVSSGLYVSVLERPADIAKL
NIPSIMAQTCRDWALFTNTTVVDQIDSGL
>KAF7936664.1 hypothetical protein EAE97_008030 [Botrytis byssoidea]
MRGLASLATLLFGSLVSAQNNYSNLPINFLKTSNPVTPLPQGFPWGNRTANNSNPYTDAPHTGVIRRYDFTVQRGQASPD
GYQRDVILVNGVFPGPLIEANWGDTIEVTVHNEIRGPEEGTALHWHGLLQKESQWFDGVPGVQQCPIPPGGTFTYSFLAD
LYGSSWWHSHYSAQYNAGIFGPMIIHGPPTVPYDYDLGPVLLNDWYHPDYYSLVEDSMSTDLNRVLVSSDSNLIQGKGFF
DCSTKAAGDNTTCNDENYMPATFKLTAGSKHRLRLINAGTEGMQKFAIDGHNMTVIANDFVPIIPYTTQVITLGVGQRTD
VIVEGLPTETTGSYWMRSTIAGCSLFKNPEAKALVYYGLEPNVNSTTTASTIFTDSVANVCANDPLNETIPWYPITPDPN
PPTTEQIDIGFGQNASGNWLWSMNNSSFRANYNEPILLLSNQGNNSYPDSPEWNVYNFGDNSSIRIYITNPTTVSHPMHL
HGHNMFVLNEGVGLWDGQTVVRPSNPQRRDVQTLQAGGYMVLQITADNPGVWPLHCHIAFHVGSGLYVSVLERPADIAKL
RIPSIMAQTCRDWAAFTNTTVVDQIDSGL
>KAF7884133.1 hypothetical protein EAF01_011556 [Botrytis porri]
MRGLASLATLLFGSLISAQNNHLTLPINFLKTSNPVTPLPQGFPWGNRTANNSNPYTDAPHTGVIRRYDFTIQRGQASPD
GYQRNVILVNGVFPGPLIEANWGDKIEVTVHNEIRGPEEGTALHWHGLLQKESQWFDGVPGVQQCPIPPGGTFTYSFLAD
LYGTSWWHSHYSAQYNAGIFGPMIIHGPPTVPYDNDLGPILLNDWYHPDYYSLVEDSMSTDPNRVFVSSDSNLIQGKGFF
DCSAKAAGDNATCNDDNYMPPTFKLTAGAKHRLRLINAGTEGMQKFAIDGHNMTVIANDFVPIIPYTTQVVTLGVGQRTD
VIVEGLPTETSGSYWMRSTIAGCSLFKNPEAKALVYYGLEPNVNSTTTASTIFTDSVANVCANDPLNETTPWYPITPDPN
PPTTEQIDIGFGKNASGNWLWSMNNSSFHANYNEPILLLSNQGNNSYPDSPEWNVYNFGDNSSIRIYITNPTTVSHPMHL
HGHNMFVLNEGVGAWDGQTVVRPSNPQRRDVQTLQAGGYMVLQITADNPGVWPLHCHIAFHVGSGLYVSVLERPADIAKL
RIPSIMAQTCRDWAAFTNTTVVDQIDSGL
>TGO35326.1 hypothetical protein BHYA_0162g00140 [Botrytis hyacinthi]
MRGLASLATLLFGSLVSAQNNYSTLPINFLKTINPVTPLPQGFPWGNRTANNSNPYTDAPHTGVIRRYDFTVQRGRASPD

GYQRDVILVNGVFPGPLIEANWGDTIEVTVHNEIRGPEEGTALHWHGLLQKESQWFDGVPGVQQCPIPPGGTFTYSFLAD
LYGSSWWHSHYSAQYNAGIFGPMIIHGPPTVPYDYDLGPILLNDWYHPDYYSLVEDSMSTDLNRVLVSSDSNLIQGKGFF
DCSTKAAGDNTTCNDENYMPPTFKLTAGSKHRLRLINAGSEGMQKFTIDGHNMTVIANDFVPIIPYTTQVVTLGVGQRTD
VIVEGLPTETTGSYWMRSTIAGCSLFKNPEAKALVYYGLEPNVNSTTTASTIFIDSVANVCANDPLNETIPWYPITPDPN
PPTMEQIDIGFGQNASGNWLWSMNNSSFRANYNEPILLLSNQGNNSYPDSPEWNVYNFGDNSSIRIYITNPTTVSHPMHL
HGHNMFVLNEGVGLWDGQTVVRPSNPQRRDVQTLQAGGYMVLQITADNPGVWPLHCHIAFHVGSGLYVSVLERPADIAKL
RIPSIMAQTCRDWAAFTNTTVVDQIDSGL
>KAF7879922.1 hypothetical protein EAF02_007559 [Botrytis sinoallii]
MRGLASLATLLFGSLVSAQNNYSNLPINFLKTSNPVTPLPQGFPWGNRTANNSNPYTDAPHTGVIRRYDFTVQRGQASPD
GYQRDVILVNGVFPGPLIEANWGDTIEVTVHNEIRGPEEGTALHWHGLLQKESQWFDGVPGVQQCPIPPGGTFTYSFLAD
LYGSSWWHSHYSAQYNAGIFGPMIIHGPPTVPYDYDLGPILLNDWYHPDYYSLVEDSMSTDLNRVLISSDSNLIQGKGFF
DCSTKAAGDNATCNVENYMPSTFKLTAGSKHRLRLINAGTEGMQKFAIDGHNMTVIANDFVPIIPYTTQVVTLGVGQRTD
VIVEGIPTETTGSYWMRSTIAGCSLFKNPEAKALVYYGLEPNVNSTTTASTIFNDSVANVCANDPLSETTPWYPITPDPN
PPTTEQIDIGFGQNASGNWLWSMNNSSFRANYNEPILLLSNQGNNSYPDSPEWNVYNFGDNSSIRIYITNPSTVSHPMHL
HGHNMFVLNEGVGLWDGRTVVRPSNPQRRDVQTLQAGGYMVLQITADNPGVWPLHCHIAFHVGSGLYVSVLERPADIAKL
RIPSIMAQTCRDWAAFTNTTVVDQIDSGL
